# Mapping review of pain management programmes and psychological therapies for community-dwelling older people living with pain

**DOI:** 10.1007/s41999-023-00871-1

**Published:** 2023-10-18

**Authors:** Natalie Lam, John Green, Sarah Hallas, Anne Forster, Thomas F. Crocker, Deirdre Andre, Alison Ellwood, Andrew Clegg, Lesley Brown

**Affiliations:** 1 Academic Unit for Ageing and Stroke Research (University of Leeds), Bradford Institute for Health Research, Bradford Royal Infirmary, Bradford Teaching Hospitals NHS Foundation Trust, Duckworth Lane, Bradford, BD9 6RJ UK; 2 University of Leeds, Leeds, UK; 3 Born in Bradford, Bradford Institute for Health Research, Bradford Teaching Hospitals NHS Foundation Trust, Bradford, UK; 4 Department of Health Sciences, University of York, York, UK

**Keywords:** Chronic pain, Aging, Frailty, Pain management program, Psychological therapy, Mapping review

## Abstract

**Purpose:**

Persistent pain is common in older people and people living with frailty. Pain or the impact of pain on everyday life is potentially modifiable. We sought to map research evidence and information from randomised controlled trials (RCTs) of pain management programmes and psychological therapies targeting community-dwelling older people, and explore appropriate strategies and interventions for managing or reducing the negative impact of pain for older people, particularly those with frailty.

**Method:**

A mapping review of pain management programmes and psychological therapies for community-dwelling older people living with chronic pain. We searched for systematic reviews of randomised controlled trials and for individual randomised controlled trials and extracted data from eligible studies.

**Results:**

Searches resulted in 3419 systematic review records and 746 RCT records from which there were 33 eligible interventions identified in 31 eligible RCTs (48 reports). Broad aims of the interventions were to: improve physical, psychological, or social functioning; adjust the effects or sensation of pain psychologically; enhance self-care with self-management skills or knowledge. Common mechanisms of change proposed were self-efficacy enhanced by self-management tasks and skills, using positive psychological skills or refocusing attention to improve responses to pain, and practising physical exercises to improve physiological well-being and reduce restrictions from pain. Content of interventions included: skills training and activity management, education, and physical exercise. Interventions were delivered in person or remotely to individuals or in groups, typically in 1–2 sessions weekly over 5–12 weeks.

**Conclusion:**

All the evaluated interventions appeared to show potential to provide some benefits to older people. None of the included studies assessed frailty. However, some of the included interventions appear appropriate for community-dwelling older people living with both frailty and pain.

**Supplementary Information:**

The online version contains supplementary material available at 10.1007/s41999-023-00871-1.

## Key summary points

**Aim:**

Review undertaken to identify and explore suitable strategies and interventions for managing or reducing the impact of pain for community-dwelling older people with frailty from the evidence in a broader population.

**Findings:**

Thirty-one randomised controlled trials (RCTs) that collectively evaluated 16 pain management programmes and 17 psychological therapies were identified. The common mechanisms of change proposed in the studies were enhancing self-efficacy, using positive psychological skills or refocusing attention to improve responses to pain, and practising physical exercises to improve physiological well-being and reduce restrictions from pain.

**Message:**

All the evaluated interventions appeared to show potential benefits to older people that may be transferable to those with frailty.

**Supplementary Information:**

The online version contains supplementary material available at 10.1007/s41999-023-00871-1.

## Introduction

Chronic pain, i.e., persistent pain of at least 3 months’ duration, is common amongst older people [1–3]. Poorly managed pain is associated with impaired activities of daily living, decreased ambulation and an increased risk of cognitive impairment [4]. Pain prevalence is particularly high in people living with frailty: 44% is the median published estimate (range 31–60%) [5]. Furthermore, it impacts more on community-dwelling older people (≥ 75 years) living with frailty compared to fit older people in the domains of mobility, ability to socialise and ability to accomplish tasks [6]. Frailty is often present with disability and comorbidity and the overlap increases with greater frailty [7]. Furthermore, there is the potential for a perpetuating cycle of pain and immobility, with further worsening of frailty [8, 9].

Pain or the impact of pain on everyday life is potentially modifiable with appropriate pain management techniques and support. However, little is known about the best strategies and interventions for managing or reducing the negative impact of pain in the older population living with frailty. The need to develop new models of care for older people, particularly those living with frailty, is highlighted in the National Health Service Long Term Plan [10].

This review aimed to map research evidence and information from RCTs of pain management programme and psychological therapies targeting community-dwelling older people. This review is part of the Pain in Older People with Frailty study [11, 12]. The objectives of this review are to: (1) identify RCTs of non-pharmacological and non-surgical pain management programmes and/or psychological therapies for persistent pain in older people (mean age ≥ 65 years) living in the community through searches for relevant systematic reviews (SRs) and recent studies; (2) describe and synthesise the content, mode of delivery, change mechanism and implementation strategies for the pain management programmes and psychological therapies in the identified RCTs, exploring their potential for improving the quality of life and other outcomes for older people including those living with frailty; and (3) identify processes and change mechanisms likely to meet the needs of older people with frailty, to inform the development of recommendations regarding content and implementation strategies as part of the POPPY study [13].

## Method

### Study design

We systematically identified pain management programmes and psychological therapies delivered to older people (mean age ≥ 65 years) with pain and pain-related conditions in RCTs, and mapped their aims, mechanisms of change, and delivery. This approach is similar to a systematic mapping of RCTs [14, 15]. Steps included: (1) setting the scope, questions and eligibility criteria; (2) searching for evidence; (3) screening evidence; (4) coding and collating information; (5) critical appraisal; and (6) describing, visualising, and reporting the findings.

We followed the enhancing transparency in reporting the synthesis of qualitative research (ENTREQ) statement in reporting the synthesis of the abstracted information [16], checklist (Supplementary materials, Appendix 1).

### Study selection

Eligible studies were RCTs which evaluated the efficacy and/or effectiveness of a pain management programme or psychological therapy in community-dwelling older people with chronic pain meeting the following criteria.

#### Population

Older people, i.e., the mean age of study participants was 65 years or older; who were community-dwelling, i.e., over 50% of participants were not living in a residential or nursing care home, hospice, or long-term care facility (defined as residents in such accommodation for over three months); and with persistent, non-specific pain or pain-related conditions of any pathology [17].

#### Interventions

Non-pharmacological and non-surgical interventions for persistent pain which were delivered as multicomponent pain management programmes or stand-alone psychological therapies, meeting the British Pain Society (BPS) [1] criteria, that an eligible intervention would "directly and indirectly produc[e] behaviour change, including methods based on cognitive and behavioural therapy" (p12). As such, participants would actively participate in some intervention component, to effect change to their cognitive, emotional, and behavioural response to pain (behavioural changes).

Pain Management Programme: We followed NICE's definition in identifying a pain management programme as "any intervention that has two or more components, including a physical and a psychological component, delivered by trained people, with some interaction/coordination between the two components" [18] (p89).

Psychological component/therapy: We used the psychological therapies identified by NICE [19] (p6-7) to identify whether a psychological intervention could be reported as a component of a pain management programme or a stand-alone therapy. The following were ineligible stand-alone interventions; biofeedback (not recommended by NICE [20]); sleep management/hygiene; and pain education which only defined pain and did not attempt to change perception or pain behaviour.

We excluded pain management programmes or psychological therapies targeting specific conditions other than osteoarthritis (OA) pain, back pain, or musculoskeletal pain, for example pain due to cancer (or receiving cancer treatment), fibromyalgia, migraine and rheumatoid arthritis.

#### Comparators

Comparators included usual care and standard available interventions.

#### Outcomes

Studies were eligible regardless of the outcome domains or length of follow-up.

#### Type of studies

RCTs and cluster RCTs including crossover designs.

#### Settings

Any setting if they met the other eligibility criteria.

#### Other criteria

Studies reported in English. We did not restrict by publication date of RCTs.

### Search strategies and selection process

Search strategies were developed in consultation with an information specialist (DA). We searched for SRs of RCTs, published from 2000, pertinent to the eligibility criteria and potentially including eligible RCTs. To account for recent research, we conducted an additional search for individual RCTs from the date of the latest search year of the most recent SRs.

The following databases were searched for SRs from 2000 to 16 December 2021: Medline, Embase, APA PsycInfo, Web of Science, Epistemonikos, and Cochrane Database of Systematic Review. We searched three databases and a trial register for RCTs from 2020 (the latest search year of the most recent SRs) to 30 June 2022: Ovid Medline, Embase, APA PsycInfo, Cochrane CENTRAL. Search strategies are available in Supplementary materials, Appendix 2.

Records identified from the literature searches were imported to EndNote (vX9.3.3) (Clarivate Analytics, Philadelphia, PA, USA) for deduplication. We used the Covidence web application (https://www.covidence.org/) (April 2022) for study selection. The results of the study selection process were then managed within EndNote. Two reviewers independently assessed the titles and abstracts from the first literature searches of SRs against our eligibility criteria and excluded obviously irrelevant SRs. Reviewers then assessed the eligibility criteria of the SRs and retained those that may have included eligible RCTs. Next, reviewers screened the included RCTs list of each relevant SR and excluded irrelevant RCTs. Additionally, they screened the titles and abstracts from the second literature searches of RCTs and excluded irrelevant RCTs. The full-text article of the potentially relevant RCTs from these two processes were then assessed against eligibility criteria to determine inclusion. Disagreements between reviewers were resolved by consensus or by consulting other authors of this review.

### Data items

We extracted data from each included RCT relating to study and participant characteristics; details of the experimental intervention and study results, including outcomes measured and potential for benefit. Full details of data extraction are in Supplementary materials, Appendix 3.

### Critical appraisal of studies

We assessed the quality of intervention reports by comparison to the template for intervention description and replication (TIDieR) [21] and study design using the Critical Appraisal Skills Programme Randomised Controlled Trial Standard Checklist (CASP for RCTs) [22].

### Abstraction process

We piloted a framework for categorising the data items and collecting details for critically appraising the intervention details and study methodology (data items and categories in Table1 and Supplementary materials Tables 1, 2, and 4–7). One reviewer extracted the data and completed the TIDieR and CASP checklist for each RCT; another reviewer independently checked the extracted details. We used NVivo 12 [23] to extract data from the reports into the piloted framework and used the functions in NVivo's "Framework Matrices" to populate the details into a matrix for each included study. We exported the completed matrix into Microsoft Excel for data collation and synthesis. Tables or matrices for the characteristics and content of included studies and interventions were produced using Microsoft Excel and Word (version 2210) (Microsoft Corporation, Redmond, Washington, USA). The characteristics of excluded studies table was produced via our EndNote library.

**Table 1:**
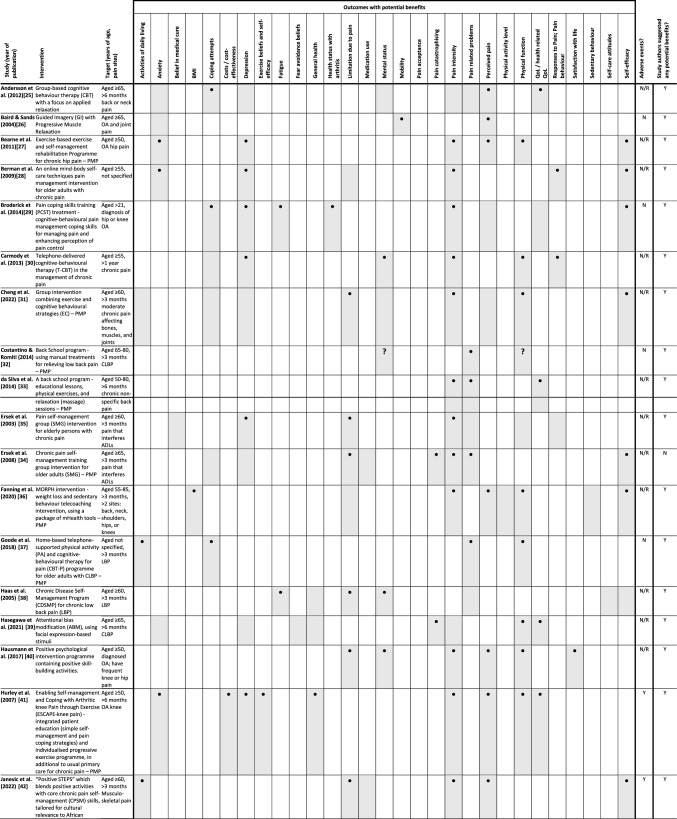

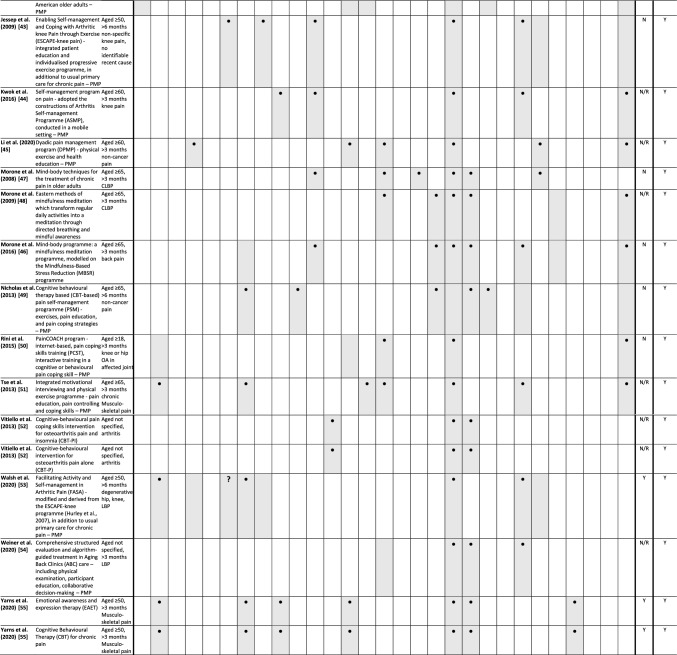
Summary characteristics and findings on included studies and interventions

*Shaded box *outcome(s) measured, **• **Study findings suggested potential benefits, ? Planned outcomes, but results not reported; *ADLs *activities of daily living, *CLBP *chronic lower back pain, *LBP *lower back pain, *N *no, *N/R *not reported, *OA *osteoarthritis, *Y *yes, *PMP *the intervention is classified to be a multicomponent pain management programme in this review

### Synthesis of results

We synthesised results from the extracted data using frequencies (counting occurrence) and thematic analysis using a deductive approach (for intervention content). The following describes specific considerations used to categorise and synthesise the results from the extracted data.

#### Intervention content

In line with our eligibility criteria, we used the NICE [19, 20] definition of pain management programmes and the list of psychological therapies to classify the physical and psychological components of each eligible intervention. We followed the description of the seven "specific cognitive and behavioural methods" explained in the British Pain Society guidelines for pain management programmes for adults (p13–15) [1] to categorise the various methods for producing behaviour change in pain management programmes.

When considering the intervention methods, we focused on those requiring behavioural or cognitive changes. Therefore, if some intervention components were pharmacological or did not involve active participation, e.g., massage, acupuncture, we did not categorise these methods in the matrix but reported them in the intervention description.

#### Intervention delivery and participants' engagement

To expand our understanding of intervention delivery and its potential to provide beneficial treatment effects, we collected information about the reasons for intervention dropouts, resources required for intervention delivery, whether the intervention was delivered as planned (intervention fidelity) and the participants’ engagement with the intervention [24].

We collected details about staff expertise, training and contribution to intervention delivery and compared the planned intervention with that delivered if both were reported. For participants' engagement, we collected details about what participants were expected to do, their use of the intervention (e.g., attendance), retention (or intervention dropouts), and satisfaction or comments about the intervention (e.g., perceived usefulness of the intervention). Satisfaction with the intervention, feasibility, fidelity, frequency of use of the intervention and compliance were specific outcomes in some studies. However, we separated them from the effectiveness outcome measures.

## Results

### Study selection

**Fig. 1:**
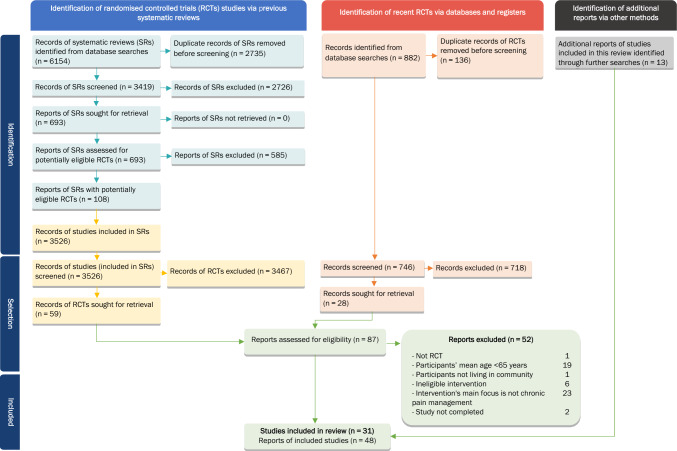
Preferred Reporting Items for Systematic Reviews and Meta-Analyses (PRISMA) flow diagram

We found 6154 records from the literature searches (conducted in December 2021) for SRs of RCTs and screened 3419 records after removing 2735 duplicates. We identified 693 records of SRs which might be relevant to our review. We reviewed the full-text reports of these 693 SRs, and identified that the eligibility criteria of 108 of the reviews suggested that eligible RCTs might be included. We screened the included studies lists of these 108 SRs. From these lists, we identified 59 RCT reports for full-text assessment, published between 1997 and 2020. From the additional literature search for RCTs published from the beginning of 2020 (recent RCTs), conducted in June 2022, we identified 746 RCT records, selecting 28 RCT reports for full-text assessment (Fig. 1).

We assessed the 87 full-text RCT reports from both literature search methods and identified 31 eligible RCT studies (48 reports) and 33 interventions to include for data synthesis.

### Included studies

We included 31 RCT studies published between 2003 and 2022 [25–55]. They originated from the United States of America (*n* = 18), Hong Kong (*n* = 4), the United Kingdom (*n* = 4), Australia, Brazil, Italy, Japan, and Sweden (*n* = 1 from each). The studies recruited 3538 people; sample sizes ranged from 21 to 418. Twenty-seven studies were parallel-group RCTs, three studies were cluster RCTs, one was a parallel-group crossover RCT (Supplementary materials, Table 2). There were 33 eligible interventions in the 31 studies, with 1872 participants in eligible intervention arms. Most interventions were delivered over 5–12 weeks, once or twice weekly. Two studies [38, 46] delivered all sessions in 6–8 weeks then provided continued support for approximately 6 months by telephone or monthly booster sessions.

Ten studies required participants to be 65 years old or above [25, 26, 32, 34, 39, 46–49, 51]; 16 studies included participants at least 50 years old; two studies included all adults from 18 [50] or 21 years old [29]; and no age criterion was specified in three studies [37, 52, 54]. Two studies placed a maximum age criterion: 80 [33] or 85 [36] years.

The conditions targeted were back or neck pain [25, 32, 33, 37–39, 46–48, 54], osteoarthritis- or arthritis-related pain [26, 27, 29, 40, 41, 50, 52], musculoskeletal pain [42, 51, 55], unspecified chronic pain or from more than one site in bones, muscles, or joints [28, 30, 31, 34–36, 45, 49, 53], and undiagnosed knee pain [43, 44]. Eleven studies excluded people with pain due to conditions which were not the target of the intervention, e.g., pain from cancer, acute injury or trauma, or infection [27, 33–35, 37, 40, 44, 46, 50, 54, 55]. Most studies specifically excluded people who could not participate in the intervention due to health conditions, e.g., mental illnesses, visual or hearing impairment, and language barriers, e.g., could not read or understand the local language. The most common exclusion criterion was cognitive impairment, if self-reported or assessed at baseline [25, 26, 29–31, 36–40, 42, 44, 46–50, 52, 54, 55].

### Excluded studies

The details of excluded studies are provided in Supplementary materials, Table 3.

### Characteristics of participants at baseline (Supplementary materials, Appendix 4)

The mean age of participants in the studies ranged from 65 to 82 years (Supplementary materials, Appendix 4). Most studies recruited more women (57–100%) than men. Ethnicity was reported in 18 studies.

The mean duration of participants' pain, reported in 13 studies, was greater than 5 years in all but one.

Mean baseline pain scores of participants, reported in 22 studies, were around the scale midpoint, except in one study [30] where participants’ self-rated mean pain scores were close to the worst possible score (bespoke pain intensity scale of 0–5).

The majority of the participants had at least one comorbidity at baseline (reported in 16 studies). Frailty status using specified frailty assessment tools was not measured or reported in any study.

Pain interference with daily activities or physical function was measured in 23 studies but was difficult to synthesise due to variation in measures and reporting. Further details are provided in Supplementary materials, Appendix 4.

### Interventions

We reported intervention details using the template for intervention description and replication (TIDieR) checklist (Supplementary materials, Table 4).

#### Intervention aims

Three broad aims of the interventions were identified (some had multiple aims): to improve physical (including improving mobility, strengthening muscles), psychological, or social functioning (20 interventions); to adjust the effects or sensation of pain psychologically (13 interventions); to enhance self-care with self-management skills or knowledge (12 interventions) (Supplementary materials, Table 5).

#### Theoretical/conceptual frameworks, mechanism of change

The included studies reported the rationale behind the intervention and the evaluation of the intervention. The mechanism of change of each intervention when reported was closely related to the aim of the intervention to bring about the targeted changes (Supplementary materials, Table 5).

The most common change mechanism was self-efficacy, enhanced by self-management tasks and skills. Through learning and continued practice of the skills, e.g., problem-solving, action planning, a person will build good habits, e.g., regular physical exercise, good posture, relaxation, and positive thinking; and his/her confidence and commitment to self-manage and self-care will increase even during pain flare-ups. A distinguishing characteristic of self-management is tailoring the intervention to individuals [56]. This was noted even in the interventions delivered via group sessions, e.g., tailoring the action plans or exercise regimen for each participant.

The common mechanisms used in psychological therapies involved positive psychological skills, e.g., cognitive restructuring, cognitive strategies, or refocusing or diverting attention, e.g., blocking painful stimuli to decrease pain sensation, training responses away from thoughts, expressions, or events associated with pain.

For interventions including a physical component, exercises aimed to improve physical capacity, endurance, strength or mobility, thereby reducing pain and restrictions caused by pain. Exercise was also incorporated into evaluation for weight management, intending to improve general health and joint function.

Theoretical frameworks were identified in thirteen studies (Supplementary materials, Table 5). The most common was Social Cognitive Theory [57] which promotes self-efficacy, with emphasis on self-management [36, 44, 50, 53]. Others included: the Stress-Appraisal Coping model of pain [30, 58]; the Bio-behavioural model of chronic pain dysfunction [52, 59]; the biopsychosocial model of pain [40, 60]; the Broaden and Build model [42, 61]; the Theory of Dyadic Illness Management [45, 62]; the Ritterband model for Internet interventions [36, 63]; and the Adult Learning Theory [50, 64].

#### Methods used in the interventions

We classified the 33 eligible interventions into stand-alone psychological therapies or pain management programmes according to whether any physical component was included. We further categorised the intervention content by 'specific cognitive and behavioural methods’ [1]. We found 19 combinations of these methods: 16 interventions were stand-alone psychological therapies in 8 combinations; 17 interventions were multicomponent pain management programmes, in 11 combinations (Supplementary materials, Table 6).

The 3 most commonly used methods among the 33 interventions were: skills training and activity management (31 interventions), education (15 interventions), and physical exercise (13 interventions). Graded exposure was not identified in any intervention. Weiner et al. [54] uniquely included physical and psychological assessments in the intervention and then tailored the treatment programme for each participant according to his/her needs and agreement.

### Mode of delivery

Seventeen interventions used single modes of delivery: 12 groups in person; 5 individuals remotely. Fourteen interventions had multiple delivery modes (e.g., starting with a baseline/orientation session to individuals in person followed by the remainder of the intervention delivered remotely or within groups), including one intervention in which participants had a tailored treatment programme following an initial assessment (Supplementary materials, Table 6) [54]. Delivery mode in two interventions was unclear.

Participants typically attended 1–2 sessions on in-person interventions weekly for a set number of weeks and then used knowledge and skills learned in daily life, e.g., integrating meditation into daily tasks, self-monitoring symptoms and achievements (Supplementary materials, Table 6). As a result, participants were expected to develop habits and master skills for continued use post intervention.

Resources required for implementing and delivering the interventions were briefly reported or implied in most studies. Hurley et al. [41] and Jessep et al. [43] reported intervention costs, yet did not report sufficient details about the cost items, e.g., costs or amount of written materials used, equipment used in the physical exercise sessions. Therefore, it is difficult to estimate the quantity and content of resources and hence the costs required of the intervention providers or participants for delivering or participating in the interventions.

### Participants' engagement

Most studies reported participant engagement (Supplementary materials, Table 7), tabulated as: use of the intervention (e.g., attendance, compliance), retention, and satisfaction.

Participants’ satisfaction with or perceived usefulness of the intervention was mostly positive (reported in 12 studies). Relaxation and physical exercise were the 2 most commonly cited useful components.

### Potential of the interventions to provide benefits

We considered the potential for benefit to be any improvement in any measured outcomes in participants after receiving the intervention, or between groups during or after the intervention, summarised in Table 1. The outcome domains and the range of measures used in studies varied. Therefore, we grouped measures which focussed on similar ideas according to the study authors' explanation and rationale for using them.

All except one study [34] reported some benefits from the experimental interventions in their conclusions (Table 1). These included improvement in the intervention arm from pre-intervention to post-intervention, the intervention feasibility, or participants' engagement.

For pain acceptance, pain catastrophising, pain intensity, pain-related problem, and perceived pain, there was the potential to provide benefit on at least one of these outcomes by all of the 33interventions.

Similarly, in outcomes related to physiological effects or functional health, namely limitations due to pain (e.g., disability), mobility, physical function, fatigue, and pain-related problems, all the interventions including a physical component showed potential to provide benefits in at least one of these outcomes.

No study reported benefits in medication use, physical activity levels, sedentary behaviour, or attitudes towards self-care. However, these outcomes were only measured in a small number of studies.

All studies included outcome measures of self-perception of well-being (e.g., general health status, mental well-being, self-efficacy), including Costantino and Romiti [32] which administered the 36-Item Short Form Health Survey (SF-36) to measure mental well-being but only reported a total score. All other 32 interventions reported potential or demonstrated benefits in at least one of these outcomes.

Two studies [41, 43] which investigated the Enabling Self-management and Coping with Arthritic knee Pain through Exercise (ESCAPE-knee pain) programme, reported cost related findings. Care home admission, hospitalisation, use of primary care and social care services were measured in these two studies only for the cost analyses. The programme, particularly if delivered in group sessions, could be cost-effective in improving physical functions; the costs of group sessions was lower than one-to-one outpatient physiotherapy.

Details of CASP assessment of the studies is provided in Supplementary materials, Appendix 5.

### Adverse events

Thirteen studies reported on adverse events: 9 studies reported no adverse events [26, 29, 32, 37, 43, 46, 47, 49, 50]; 4 studies reported adverse events experienced by a small proportion of the participants who received multicomponent pain management programmes [41, 42, 53, 55] and that most were likely related to the physical component, e.g., exacerbation of pain (*n* = 3/278 in intervention arms) [41, 55] (Table 1).

## Discussion

From 31 RCTs (48 reports) 33 eligible persistent pain management interventions were identified. All of these studies included participants of mean age 65 years or over. None provided a validated measure of frailty or discussed the results within the context of frailty. The common mechanisms of change proposed in the studies were self-efficacy enhanced by self-management tasks and skills, using positive psychological skills or refocusing attention to improve responses to pain, and practising physical exercises to improve physiological well-being and reduce restrictions from pain. The interventions were delivered by trained healthcare professionals, researchers, or peer-volunteers, primarily via face-to-face and/or group sessions. Telephone, internet, and mobile-phone apps were incorporated in some studies.

The most commonly used method in the 33 eligible interventions was skills training and activity management. Most interventions lasted 5–12 weeks, with sessions held once or twice weekly. All the interventions appeared to promote and expect the participants to continue using the acquired skills beyond the intervention period. Practising self-management skills, self-care and continued use of these skills to prepare for flare-ups are important. The duration of an intervention may only last a few weeks. However, the continued use of acquired skills and knowledge can be beneficial in the longer term. However, only 13 studies evaluated the treatment effects at or beyond 6 months.

Most participants engaged positively and completed most intervention sessions. According to the participants in five studies, relaxation and physical exercise were useful intervention components. Specific considerations adopted in four interventions, which specifically targeted “older” people, included simplified CBT sessions (for people aged ≥ 65) [25], delivering self-care tools online to overcome logistic barriers of in-person sessions (for people aged ≥ 55) [28], recruiting and involving informal caregivers in dyadic sessions (for people aged ≥ 60) [45] and ADL training (for people aged ≥ 60) [44]. The outcome findings, participants' engagement, satisfaction, and comments were generally positive; this suggests that potentially all these interventions would be feasible, acceptable and beneficial to older people with persistent pain.

Previous SRs have investigated the treatment effects of persistent pain interventions in older people. Mixed physiotherapy modalities [58] and health education programs [59] improved physical function and reduced pain. Psychological interventions [60] might improve self-efficacy and reduce pain. An integrated pain management approach [61], client-centred occupational therapy, and self-management programmes with cognitive-behavioural principles [59] were found to improve participation and patient–clinician therapeutic alliance [59, 61]. However, there was also a suggestion that the evidence in support of self-management (Stanford model of self-management or close derivatives) was not convincing [62]. They suggested that more research was needed to determine the best treatment and delivery strategies (e.g., content, duration, format) to older adults for sustainable effects [60, 63].

It has been proposed that persistent pain and comorbidities in older people commonly co-exist, leading to beliefs and misperception that pain is an inevitable part of ageing and therefore should be tolerated [44, 64, 65]. Furthermore, pain may be underreported, and older people may experience "age-related bias", which limits referral for interventions [64, 65]. Improvement in functional outcomes, e.g., mobility, may be achievable through comprehensive evaluations, an intervention targeting multiple pain sources, and correcting the misconception about the inevitability of age-related pain [64].

NICE recommends assessments to identify factors contributing to persistent pain and its effects on a person's life, providing advice and information at all stages of care, and collaborative care planning to support self-management of chronic pain in adults [20]. Similarly, the British Pain Society suggests using education about pain, general health and pain self-management in pain management programmes, which can also include physical exercises, activity management, and cognitive and behavioural therapies [1].

We did not undertake independent quantitative or statistical synthesis and instead relied on reported findings and conclusions. However, assessing the quality of the RCTs and the reported intervention details using TIDieR and CASP checklists aided our analyses of the potential to provide benefits, the transferability or generalisability of the interventions, and possible effects on the target population.

We were unable to investigate which specific cognitive and behavioural methods may be more suitable for the target population of older people with frailty, or provide more benefits to the study participants, because the methods were usually combined in a package of a multicomponent pain management programme or psychological therapy. Individual methods often have synergistic or dis-synergistic effects on each other in a pain management intervention and the context or implementation of the package of intervention [66]. All studies reported the intervention content and at least some of the resources utilised, though details were often brief. Only one research team, which evaluated the Enabling Self-management and Coping with Arthritic knee Pain through Exercise (ESCAPE-knee pain) programme, reported cost related findings [41, 43]. The information is insufficient for confident replication of any included intervention.

RCTs were identified from published SRs and individual RCTs published after the latest search year of the most recent SRs. Therefore, we relied on the SRs being published and having conducted comprehensive literature searches, specified eligibility criteria, and being correctly indexed.

We only included RCTs published in English; therefore, interventions which may have been published in other languages may have been omitted.

No assessment of frailty was reported by any study. However, according to the baseline characteristics of all included samples, pain duration, pain score, number of comorbidities, pain interference with daily activities or physical function, and mental health status, on average, revealed that most participants had lived with persistent pain for many years. Although the scores varied between studies, they were similar or slightly worse than the normative values of people with persistent pain, or similar to the general population in some studies and outcome measures. When specifically reported, comorbidities were common in the study samples. However, people with unstable physiological or psychological conditions were often excluded.

Many studies had some exclusion criteria, e.g., mental illnesses, visual or hearing impairment; with the most common exclusion being cognitive impairment (an exclusion in twenty studies) if self-reported or assessed at baseline. Furthermore, language and physical barriers were exclusions to attending group sessions, when group format was the main intervention delivery mode. Overall, older people with frailty were probably represented in some of the included studies. However, some older people with frailty were likely excluded due to cognitive, visual, hearing loss and physical barriers.

According to the study eligibility criteria, ten studies specifically targeted people aged 65 or over, but only Andersson et al. [25] reported specific customisation for this age group. All interventions targeted people with persistent pain of at least 3–6 months, a relatively short period compared to the reported duration of persistent pain of the participants. This suggests discrepancies between the clinical classification of persistent pain and people's perceived understanding and acceptance of "chronic pain" before they consider seeking treatments, and when and whether to consider pharmacological or non-pharmacological treatments [26, 31, 42, 47].

Most interventions included a range of simplified, easy to follow content, appropriate activities and activity levels, providing dyadic sessions, and training and practice sessions for using new technology and delivering the intervention via the internet. Various delivery modes, including online materials and mobile-phone apps, are feasible in providing the resources to many older people for skills training and practice. However, the review findings were predominantly based on interventions targeting the "younger old" people, aged from 50 years, with persistent pain, some with comorbidities and problems with physical function. There remain some older people, particularly those in later years and with more advanced frailty that do not have ready access to online resources or mobile-phone apps [67] and may require materials in alternative formats.

In conclusion, the evaluated interventions appeared to show the potential to provide some benefits to older adults. Some of these interventions may be transferable or adaptable to older adults living with frailty. Pain impact is potentially modifiable, and therefore should make an attractive target for services for older people living with both frailty and pain.

### Supplementary Information

Below is the link to the electronic supplementary material.

Supplementary file1 (DOCX 236 KB)

## Data Availability

The manuscript has no associated data or the data will not be deposited. All data generated during this study are contained in this published article and its Supplementary Information, along with the original references describing the randomised control trials included in this mapping review.
