# Supplementary material for: Mapping review of pain management programmes and psychological therapies for community-dwelling older people living with pain
Source: Eur Geriatr Med. 2023 Oct 18;15(1):33–45. doi: 10.1007/s41999-023-00871-1 (PMC10876761; doi:10.1007/s41999-023-00871-1)
Supplement: Supplementary file 1 — Supplementary file1 (DOCX 236 KB) [file 41999_2023_871_MOESM1_ESM.docx]

**Supplementary materials**

**Appendices**

| 1 | Enhancing transparency in reporting the synthesis of qualitative research ENTREQ statement in reporting the synthesis of the abstracted information checklist | 3 |
| --- | --- | --- |
| 2 | Search strategies | 5 |
| 3 | Details of data extraction | 16 |
| 4 | Characteristics of participants | 17 |
| 5 | CASP assessment | 19 |

**Tables**

| 1 | CASP assessment | 22 |
| --- | --- | --- |
| 2 | Characteristics of included studies | 24 |
| 3 | Excluded studies | 75 |
| 4 | TIDieR checklist | 77 |
| 5 | Intervention delivery and mechanism matrix | 79 |
| 6 | Intervention details | 96 |
| 7 | Participant engagement | 106 |

**Appendices**

**Appendix 1. Enhancing transparency in reporting the synthesis of qualitative research ENTREQ statement in reporting the synthesis of the abstracted information checklist**

| **No** | **Item** | **Guide and description** | **Reported on (Page number and sessions)** |
| --- | --- | --- | --- |
| 1 | Aim | State the research question the synthesis addresses. | Page 4 |
| 2 | Synthesis methodology | Identify the synthesis methodology or theoretical framework which underpins the synthesis, and describe the rationale for choice of methodology (e.g. meta-ethnography, thematic synthesis, critical interpretive synthesis, grounded theory synthesis, realist synthesis, meta-aggregation, meta-study, framework synthesis). | Page 5-10 |
| 3 | Approach to searching | Indicate whether the search was pre-planned (comprehensive search strategies to seek all available studies) or iterative (to seek all available concepts until they theoretical saturation is achieved). | Page 7-8 |
| 4 | Inclusion criteria | Specify the inclusion/exclusion criteria (e.g. in terms of population, language, year limits, type of publication, study type). | Page 5-7 |
| 5 | Data sources | Describe the information sources used (e.g. electronic databases (MEDLINE, EMBASE, CINAHL, psycINFO, Econlit), grey literature databases (digital thesis, policy reports), relevant organisational websites, experts, information specialists, generic web searches (Google Scholar) hand searching, reference lists) and when the searches conducted; provide the rationale for using the data sources. | Page 7-8, 10 |
| 6 | Electronic Search strategy | Describe the literature search (e.g. provide electronic search strategies with population terms, clinical or health topic terms, experiential or social phenomena related terms, filters for qualitative research, and search limits). | Page 7-8 and Appendix 2 |
| 7 | Study screening methods | Describe the process of study screening and sifting (e.g. title, abstract and full text review, number of independent reviewers who screened studies). | Page 7-8, 10 |
| 8 | Study characteristics | Present the characteristics of the included studies (e.g. year of publication, country, population, number of participants, data collection, methodology, analysis, research questions). | Page 10, 27-48 (Table 1), and Appendix Tables 1, 2 & 4 |
| 9 | Study selection results | Identify the number of studies screened and provide reasons for study exclusion (e,g, for comprehensive searching, provide numbers of studies screened and reasons for exclusion indicated in a figure/flowchart; for iterative searching describe reasons for study exclusion and inclusion based on modifications t the research question and/or contribution to theory development). | Page 10-11 and 33-34 (Fig 1) |
| 10 | Rationale for appraisal | Describe the rationale and approach used to appraise the included studies or selected findings (e.g. assessment of conduct (validity and robustness), assessment of reporting (transparency), assessment of content and utility of the findings). | Page 8 |
| 11 | Appraisal items | State the tools, frameworks and criteria used to appraise the studies or selected findings (e.g. Existing tools: CASP, QARI, COREQ, Mays and Pope [25]; reviewer developed tools; describe the domains assessed: research team, study design, data analysis and interpretations, reporting). | Page 8, Appendix 5, and Appendix Table 3 |
| 12 | Appraisal process | Indicate whether the appraisal was conducted independently by more than one reviewer and if consensus was required. | Page 8-9 and Appendix 5 |
| 13 | Appraisal results | Present results of the quality assessment and indicate which articles, if any, were weighted/excluded based on the assessment and give the rationale. | Appendix 5 |
| 14 | Data extraction | Indicate which sections of the primary studies were analysed and how were the data extracted from the primary studies? (e.g. all text under the headings “results /conclusions” were extracted electronically and entered into a computer software). | Page 8-9 |
| 15 | Software | State the computer software used, if any. | Page 7-8 |
| 16 | Number of reviewers | Identify who was involved in coding and analysis. | Page 8-9, 21 |
| 17 | Coding | Describe the process for coding of data (e.g. line by line coding to search for concepts). | Page 8-9 |
| 18 | Study comparison | Describe how were comparisons made within and across studies (e.g. subsequent studies were coded into pre-existing concepts, and new concepts were created when deemed necessary). | Page 8-10 |
| 19 | Derivation of themes | Explain whether the process of deriving the themes or constructs was inductive or deductive. | Page 8-10 |
| 20 | Quotations | Provide quotations from the primary studies to illustrate themes/constructs, and identify whether the quotations were participant quotations of the author’s interpretation. | Table 1 and Appendix Tables 4-7  (We occasionally extracted direct quotations and mainly summarised from them in the report.) |
| 21 | Synthesis output | Present rich, compelling and useful results that go beyond a summary of the primary studies (e.g. new interpretation, models of evidence, conceptual models, analytical framework, development of a new theory or construct). | Page 10-21 |

**Appendix 2 Search strategies**

**Systematic Review search strategies**

**SR**Database: Ovid MEDLINE(R) ALL <1946 to December 15, 2021>

Search Strategy:

--------------------------------------------------------------------------------

1 pain, intractable/ (6296)

2 chronic pain/ (18557)

3 ((persist* or intract* or idiopathic or atypical or "a typical" or chronic or prolong* or long last* or sustain* or longstanding or long standing or longterm or long term or refractory or sustain* or linger* or syndrome*) adj5 pain*).tw,kf. (126960)

4 or/1-3 [chronic pain] (132609)

5 Pain Management/ (38372)

6 (pain management adj2 (program* or rehab*)).tw,kf. (921)

7 ((management or rehab*) adj3 (program* or course* or session* or group* or class* or scheme* or strateg* or initiative* or training)).tw,kf. (119931)

8 self care/ (34921)

9 (selfcar* or selfmanagement or selfhelp or selfadministrat* or selfmonitor* or selfmedicat*).tw,kf. (267)

10 (Self adj2 (car* or manag* or program* or help or admistrat* or monitor* or medicat*)).tw,kf. (73523)

11 Self-Help Groups/ (9424)

12 telemedicine/ (31614)

13 telerehabilitation/ (684)

14 telehealth/ (31614)

15 ((tele adj2 (heal* or medicine or care)) or telehealth or telemedicine or telecare).tw,kf. (26086)

16 pain education.tw,kf. (593)

17 patient education as topic/ (87703)

18 health education/ (62528)

19 teaching/ (50938)

20 or/5-18 [pain management programmes] (418041)

21 exp psychotherapy/ (208480)

22 psychotherap*.tw,kf. (50012)

23 (psychological adj (treatment* or therapy or therapies)).tw,kf. (6756)

24 (group* adj3 (therap* or program*)).tw,kf. (47618)

25 exp mind-body therapies/ (53422)

26 hypnosis.tw,kf. (8388)

27 biofeedback.tw,kf. (7528)

28 "eye movement desensiti?ation and reprocessing".tw,kf. (644)

29 ((behavio?r* or cognitive or relax* or psycho* or compassion or solution) adj3 (technique* or therap* or treatment* or training or rehab*)).tw,kf. (131066)

30 (CBT or CBASP or EMDR).tw,kf. (13500)

31 (acceptance adj3 therap*).tw,kf. (1993)

32 (commitment adj3 therap*).tw,kf. (1487)

33 meditat*.tw,kf. (7208)

34 guided imagery.tw,kf. (811)

35 mindfulness.tw,kf. (9947)

36 (sleep adj3 (manag* or program* or regulat* or therap*)).tw,kf. (10134)

37 adaptation, psychological/ (100178)

38 coping skill*.tw,kf. (3577)

39 or/21-38 [psychological therapies] (470213)

40 20 or 39 [pain management programmes or psychological therapies] (847998)

41 "systematic review"/ (179666)

42 Meta-Analysis/ (149097)

43 exp Meta-Analysis as Topic/ (23617)

44 (meta analy* or metanaly* or metaanaly* or meta regression).ti,ab. (222005)

45 ((systematic* or evidence*) adj3 (review* or overview*)).ti,ab. (291950)

46 (reference list* or bibliograph* or hand search* or manual search* or relevant journals).ab. (48982)

47 (search strategy or search criteria or systematic search or study selection or data extraction).ab. (67706)

48 (search* adj4 literature).ab. (80956)

49 (medline or pubmed or cochrane or embase or psychlit or psyclit or psychinfo or psycinfo or cinahl or science citation index or bids).ab. (293021)

50 cochrane.jw. (15781)

51 ((multiple treatment* or indirect or mixed) adj2 comparison*).ti,ab. (3318)

52 or/41-51 (578486)

53 letter/ (1162808)

54 editorial/ (589856)

55 news/ (210577)

56 exp historical article/ (406575)

57 anecdotes as Topic/ (4746)

58 comment/ (943079)

59 case report/ (2233045)

60 (letter or comment*).ti. (172098)

61 or/53-60 (4670219)

62 52 not 61 [Adapted from systematic review search filter used by NICE] (547263)

63 4 and 40 and 62 [chronic pain and pain management programmes or psychological therapies and systematic reviews] (1964)

64 exp animals/ not humans.sh. (4931018)

65 63 not 64 [human only studies] (1958)

66 (exp adolescent/ or exp child/ or exp newborn/) not ((exp adult/ or exp aged/ or exp middle aged/) and (exp adolescent/ or exp child/ or exp newborn/)) (1872193)

67 65 not 66 [excluding studies about children] (1865)

68 ((cancer adj2 pain) not ("non cancer pain" or "noncancer pain")).ti. (5037)

69 67 not 68 [excluding studies about cancer pain in title] (1829)

70 limit 69 to yr="2000 -Current" (1792)

**Randomised controlled trials search strategies**

Database: Ovid MEDLINE(R) ALL <1946 to June 29, 2022>

Search Strategy:

--------------------------------------------------------------------------------

1 Pain, Intractable/ (6331)

2 chronic pain/ (20156)

3 ((persist* or intract* or idiopathic or atypical or "a typical" or chronic or prolong* or long last* or sustain* or longstanding or long standing or longterm or long term or refractory or linger* or syndrome*) adj5 pain*).tw,kf. (132020)

4 or/1-3 [chronic pain] (137738)

5 Pain Management/ (39465)

6 (pain management adj2 (program* or rehab*)).tw,kf. (967)

7 ((management or rehab*) adj3 (program* or course* or session* or group* or class* or scheme* or strateg* or initiative* or training)).tw,kf. (125715)

8 Self Care/ (35400)

9 (selfcar* or selfmanagement or selfhelp or selfadministrat* or selfmonitor* or selfmedicat*).tw,kf. (293)

10 (Self adj2 (car* or manag* or program* or help or admistrat* or monitor* or medicat*)).tw,kf. (77324)

11 Self-Help Groups/ (9482)

12 Telemedicine/ (34037)

13 Telerehabilitation/ (808)

14 ((tele adj2 (heal* or medicine or care)) or telehealth or telemedicine or telecare).tw,kf. (29363)

15 pain education.tw,kf. (639)

16 Patient Education as Topic/ (88081)

17 Health Education/ (62970)

18 Teaching/ (51356)

19 or/5-18 [pain management programmes] (481003)

20 exp Psychotherapy/ (212665)

21 psychotherap*.tw,kf. (51301)

22 (psychological adj (treatment* or therapy or therapies)).tw,kf. (7056)

23 (group* adj3 (therap* or program*)).tw,kf. (49234)

24 exp Mind-Body Therapies/ (45946)

25 hypnosis.tw,kf. (8509)

26 biofeedback.tw,kf. (7774)

27 "eye movement desensiti?ation and reprocessing".tw,kf. (666)

28 ((behavio?r* or cognitive or relax* or psycho* or compassion or solution) adj3 (technique* or therap* or treatment* or training or rehab*)).tw,kf. (136096)

29 (CBT or CBASP or EMDR).tw,kf. (14232)

30 (acceptance adj3 therap*).tw,kf. (2187)

31 (commitment adj3 therap*).tw,kf. (1667)

32 meditat*.tw,kf. (7625)

33 guided imagery.tw,kf. (847)

34 mindfulness.tw,kf. (10967)

35 Mindfulness/ (5399)

36 (sleep adj3 (manag* or program* or regulat* or therap*)).tw,kf. (10508)

37 Adaptation, Psychological/ (101799)

38 coping skill*.tw,kf. (3750)

39 or/20-38 [psychological therapies] (475041)

40 19 or 39 [pain management or psychological therapies] (911902)

41 randomized controlled trial.pt. (571827)

42 controlled clinical trial.pt. (94924)

43 randomized.ab. (566615)

44 placebo.ab. (229523)

45 drug therapy.fs. (2505782)

46 randomly.ab. (385683)

47 trial.ab. (606130)

48 groups.ab. (2372385)

49 or/41-48 (5393958)

50 exp animals/ not humans.sh. (5022766)

51 49 not 50 [Cochrane Cochrane Highly Sensitive Search Strategy for identifying randomized trials in MEDLINE] (4696754)

52 4 and 40 and 51 [chronic pain and pain management or psychological therapy and RCTs] (8976)

53 exp Aged/ (3405047)

54 Geriatrics/ (31090)

55 Geriatric Assessment/ (31266)

56 (gerontol* or ageing or aging or elder* or geriatric* or senior* or old age* or "older person*" or "older people" or "older adult" or late* life or very old or oldest old).tw,kf. (692751)

57 or/53-56 [aged 65 and over] (3741261)

58 52 and 57 [limited to aged 65 and over] (2278)

59 ((cancer adj2 pain) not ("non cancer pain" or "noncancer pain")).ti. (5155)

60 58 not 59 [excluding cancer pain] (2231)

61 limit 60 to yr="2020 -Current" (284)

***************************

Database: Embase <1996 to 2022 Week 25>

Search Strategy:

--------------------------------------------------------------------------------

1 intractable pain/ (3837)

2 chronic pain/ (67620)

3 ((persist* or intract* or idiopathic or atypical or "a typical" or chronic or prolong* or long last* or sustain* or longstanding or long standing or longterm or long term or refractory or linger* or syndrome*) adj5 pain*).tw,kf. (176986)

4 or/1-3 [chronic pain] (192265)

5 analgesia/ (122186)

6 (pain management adj2 (program* or rehab*)).tw,kf. (1322)

7 ((management or rehab*) adj3 (program* or course* or session* or group* or class* or scheme* or strateg* or initiative* or training)).tw,kf. (162134)

8 self care/ (64562)

9 (selfcar* or selfmanagement or selfhelp or selfadministrat* or selfmonitor* or selfmedicat*).tw,kf. (4336)

10 self help/ (10656)

11 (Self adj2 (car* or manag* or program* or help or admistrat* or monitor* or medicat*)).tw,kf. (99374)

12 telemedicine/ (37726)

13 telerehabilitation/ (1839)

14 teletherapy/ (834)

15 ((tele adj2 (heal* or medicine or care)) or telehealth or telemedicine or telecare).tw,kf. (38037)

16 pain education.tw,kf. (924)

17 patient education/ (103434)

18 health education/ (79094)

19 teaching/ (81096)

20 or/5-19 [pain management programmes] (678277)

21 exp psychotherapy/ (216732)

22 psychotherap*.tw,kf. (50630)

23 (psychological adj (treatment* or therapy or therapies)).tw,kf. (9257)

24 (group* adj3 (therap* or program*)).tw,kf. (66850)

25 alternative medicine/ (45288)

26 hypnosis.tw,kf. (6020)

27 biofeedback.tw,kf. (8749)

28 "eye movement desensiti?ation and reprocessing".tw,kf. (792)

29 biofeedback/ (3014)

30 ((behavio?r* or cognitive or relax* or psycho* or compassion or solution) adj3 (technique* or therap* or treatment* or training or rehab*)).tw,kf. (160918)

31 (CBT or CBASP or EMDR).tw,kf. (20737)

32 (acceptance adj3 therap*).tw,kf. (2982)

33 (commitment adj3 therap*).tw,kf. (2257)

34 meditat*.tw,kf. (9649)

35 guided imagery.tw,kf. (1114)

36 mindfulness.tw,kf. (13954)

37 meditation/ (8299)

38 (sleep adj3 (manag* or program* or regulat* or therap*)).tw,kf. (13717)

39 psychological adjustment/ (1718)

40 coping skill*.tw,kf. (4826)

41 coping behavior/ (64987)

42 or/21-41 [psychological therapies] (497898)

43 20 or 42 [pain management or psychological therapies] (1120573)

44 Randomized controlled trial/ (669354)

45 Controlled clinical study/ (422095)

46 random$.ti,ab. (1670358)

47 randomization/ (85803)

48 intermethod comparison/ (275649)

49 placebo.ti,ab. (294680)

50 (compare or compared or comparison).ti. (453469)

51 ((evaluated or evaluate or evaluating or assessed or assess) and (compare or compared or comparing or comparison)).ab. (2396084)

52 (open adj label).ti,ab. (95957)

53 ((double or single or doubly or singly) adj (blind or blinded or blindly)).ti,ab. (210310)

54 double blind procedure/ (170290)

55 parallel group$1.ti,ab. (27398)

56 (crossover or cross over).ti,ab. (97120)

57 ((assign$ or match or matched or allocation) adj5 (alternate or group$1 or intervention$1 or patient$1 or subject$1 or participant$1)).ti,ab. (350557)

58 (assigned or allocated).ti,ab. (411477)

59 (controlled adj7 (study or design or trial)).ti,ab. (380362)

60 (volunteer or volunteers).ti,ab. (219596)

61 human experiment/ (452418)

62 trial.ti. (330698)

63 or/44-62 (5208249)

64 (random$ adj sampl$ adj7 (cross section$ or questionnaire$1 or survey$ or database$1)).ti,ab. not (comparative study/ or controlled study/ or randomi?ed controlled.ti,ab. or randomly assigned.ti,ab.) (8155)

65 Cross-sectional study/ not (randomized controlled trial/ or controlled clinical study/ or controlled study/ or randomi?ed controlled.ti,ab. or control group$1.ti,ab.) (308047)

66 (((case adj control$) and random$) not randomi?ed controlled).ti,ab. (18944)

67 (Systematic review not (trial or study)).ti. (212657)

68 (nonrandom$ not random$).ti,ab. (15364)

69 Random field$.ti,ab. (2660)

70 (review.ab. and review.pt.) not trial.ti. (969462)

71 we searched.ab. and (review.ti. or review.pt.) (42322)

72 update review.ab. (111)

73 (databases adj4 searched).ab. (51375)

74 (rat or rats or mouse or mice or swine or porcine or murine or sheep or lambs or pigs or piglets or rabbit or rabbits or cat or cats or dog or dogs or cattle or bovine or monkey or monkeys or trout or marmoset$1).ti. and animal experiment/ (795533)

75 Animal experiment/ not (human experiment/ or human/) (1653526)

76 or/64-75 (3168076)

77 63 not 76 [Cochrane Highly Sensitive Search Strategy for identifying controlled trials in Embase] (4587931)

78 4 and 43 and 77 [chronic pain and pain management or psychological therapy and RCTs] (11575)

79 exp aged/ (2896640)

80 geriatrics/ (25579)

81 geriatric assessment/ (18681)

82 (gerontol* or ageing or aging or elder* or geriatric* or senior* or old age* or "older person*" or "older people" or "older adult" or late* life or very old or oldest old).tw,kf. (794639)

83 or/79-82 [aged 65 and over] (3279485)

84 78 and 83 [limited to aged 65 and over] (2479)

85 ((cancer adj2 pain) not ("non cancer pain" or "noncancer pain")).ti. (5903)

86 84 not 85 [excluding cancer pain] (2426)

87 limit 86 to yr="2020 -Current" (458)

***************************

Database: APA PsycInfo <2002 to June Week 3 2022>

Search Strategy:

--------------------------------------------------------------------------------

1 chronic pain/ (11898)

2 ((persist* or intract* or idiopathic or atypical or "a typical" or chronic or prolong* or long last* or sustain* or longstanding or long standing or longterm or long term or refractory or linger* or syndrome*) adj5 pain*).tw. (24134)

3 or/1-2 [chronic pain] (24832)

4 pain management/ (8773)

5 (pain management adj2 (program* or rehab*)).tw. (362)

6 ((management or rehab*) adj3 (program* or course* or session* or group* or class* or scheme* or strateg* or initiative* or training)).tw. (32185)

7 self-care/ (2742)

8 (selfcar* or selfmanagement or selfhelp or selfadministrat* or selfmonitor* or selfmedicat*).tw. (191)

9 self-help techniques/ (2535)

10 (Self adj2 (car* or manag* or program* or help or admistrat* or monitor* or medicat*)).tw. (36066)

11 self-management/ (5608)

12 support groups/ (2908)

13 telemedicine/ (6736)

14 telerehabilitation/ (194)

15 telepsychology/ (197)

16 ((tele adj2 (heal* or medicine or care)) or telehealth or telemedicine or telecare).tw. (5431)

17 pain education.tw. (188)

18 client education/ (2735)

19 health education/ (10024)

20 teaching/ (40284)

21 or/4-20 [pain management programmes] (133906)

22 exp psychotherapy/ (119650)

23 psychotherap*.tw. (68437)

24 (psychological adj (treatment* or therapy or therapies)).tw. (7547)

25 (group* adj3 (therap* or program*)).tw. (18146)

26 mind body therapy/ (323)

27 hypnosis.tw. (4056)

28 hypnosis/ (2750)

29 biofeedback.tw. (2109)

30 biofeedback/ (1067)

31 "eye movement desensiti?ation and reprocessing".tw. (1544)

32 ((behavio?r* or cognitive or relax* or psycho* or compassion or solution) adj3 (technique* or therap* or treatment* or training or rehab*)).tw. (112400)

33 (CBT or CBASP or EMDR).tw. (17215)

34 (acceptance adj3 therap*).tw. (3167)

35 (commitment adj3 therap*).tw. (2967)

36 meditat*.tw. (8458)

37 meditation/ (3989)

38 guided imagery.tw. (814)

39 mindfulness.tw. (17255)

40 mindfulness/ (11583)

41 (sleep adj3 (manag* or program* or regulat* or therap*)).tw. (3565)

42 coping skill*.tw. (4363)

43 coping behavior/ (32969)

44 or/22-43 [psychological therapies] (279398)

45 21 or 44 [pain management or psychological therapies] (395991)

46 exp Clinical Trials/ or Placebo/ or (random* or sham or placebo* or ((singl* or doubl*) adj (blind* or dumm* or mask*)) or ((tripl* or trebl*) adj (blind* or dumm* or mask*)) or (control* adj3 (study or studies or trial* or group*)) or Nonrandom* or non random* or non-random* or quasi-random* or quasirandom* or allocated or ((open label or open-label) adj5 (study or studies or trial*)) or ((equivalence or superiority or non-inferiority or noninferiority) adj3 (study or studies or trial*)) or ((pragmatic or practical) adj3 trial*) or ((quasiexperimental or quasi-experimental) adj3 (study or studies or trial*)) or (phase adj3 (III or "3") adj3 (study or studies or trial*))).ti,ab,hw. [CADTH’s database search filters - randomized controlled trials / controlled clinical trials - PsycInfo] (291401)

47 3 and 45 and 46 [chronic pain and pain management or psychological therapy and RCTs] (1748)

48 aging/ (61169)

49 geriatrics/ (10618)

50 older adulthood/ (7250)

51 geriatric assessment/ (757)

52 (gerontol* or ageing or aging or elder* or geriatric* or senior* or old age* or "older person*" or "older people" or "older adult" or late* life or very old or oldest old).tw. (146015)

53 or/48-52 [aged 65 and over] (157310)

54 47 and 53 [limited to aged 65 and over] (85)

55 ((cancer adj2 pain) not ("non cancer pain" or "noncancer pain")).ti. (742)

56 54 not 55 [excluding cancer patients] (85)

57 limit 56 to yr="2020" (3)

***************************

CENTRAL Search strategy

Search Name: Brown pain management6

Date Run: 30/06/2022 16:23:18

Comment: SD 23/06/22

ID Search Hits

#1 MeSH descriptor: [Pain, Intractable] this term only 275

#2 MeSH descriptor: [Chronic Pain] this term only 3064

#3 ((persist* or intract* or idiopathic or atypical or "a typical" or chronic or prolong* or "long last*" or sustain* or longstanding or "long standing" or longterm or "long term" or refractory or linger* or syndrome*) NEAR/5 pain*):ti,ab,kw (Word variations have been searched) 30269

#4 #1 or #2 or #3 30269

#5 MeSH descriptor: [Pain Management] this term only 4344

#6 ("pain management" NEAR/2 (program* or rehab*)):ti,ab,kw (Word variations have been searched) 253

#7 ((management or rehab*) NEAR/3 (program* or course* or session* or group* or class* or scheme* or strateg* or initiative* or training)):ti,ab,kw (Word variations have been searched) 29451

#8 MeSH descriptor: [Self Care] this term only 4362

#9 ((selfcar* or selfmanagement or selfhelp or selfadministrat* or selfmonitor* or selfmedicat*)):ti,ab,kw (Word variations have been searched) 22207

#10 (self NEAR/2 (car* or manag* or program* or help or admistrat* or monitor* or medicat*)):ti,ab,kw (Word variations have been searched) 27689

#11 MeSH descriptor: [Self-Help Groups] this term only 741

#12 MeSH descriptor: [Telemedicine] this term only 2714

#13 MeSH descriptor: [Telerehabilitation] this term only 170

#14 ((tele NEAR/2 (heal* or medicine or care)) or (telehealth or telemedicine or telecare)):ti,ab,kw (Word variations have been searched) 6810

#15 ("pain education"):ti,ab,kw (Word variations have been searched) 329

#16 MeSH descriptor: [Patient Education as Topic] this term only 9229

#17 MeSH descriptor: [Health Education] this term only 4195

#18 MeSH descriptor: [Teaching] this term only 1817

#19 #5 or #6 or #7 or #8 or #9 or #10 or #11 or #12 or #13 or #14 or #15 or #16 or #17 or #18 74808

#20 MeSH descriptor: [Psychotherapy] explode all trees 26860

#21 (psychotherap*):ti,ab,kw (Word variations have been searched) 14873

#22 (psychological NEAR/1 (treatment* or therapy or therapies)):ti,ab,kw (Word variations have been searched) 25006

#23 (group* NEAR/3 (therap* or program*)):ti,ab,kw (Word variations have been searched) 34731

#24 MeSH descriptor: [Mind-Body Therapies] explode all trees 6932

#25 (hypnosis):ti,ab,kw (Word variations have been searched) 1767

#26 (biofeedback):ti,ab,kw (Word variations have been searched) 3733

#27 ("eye movement desensiti?ation and reprocessing"):ti,ab,kw (Word variations have been searched) 0

#28 ((behavio?r* or cognitive or relax* or psycho* or compassion or solution) NEAR/3 (technique* or therap* or treatment* or training or rehab*)):ti,ab,kw (Word variations have been searched) 84609

#29 ((CBT or CBASP or EMDR)):ti,ab,kw (Word variations have been searched) 9743

#30 ((acceptance NEAR/3 therap*)):ti,ab,kw (Word variations have been searched) 2505

#31 (commitment NEAR/3 therap*):ti,ab,kw (Word variations have been searched) 1419

#32 (meditat*):ti,ab,kw (Word variations have been searched) 3695

#33 ("guided imagery"):ti,ab,kw (Word variations have been searched) 749

#34 (mindfulness):ti,ab,kw (Word variations have been searched) 11218

#35 MeSH descriptor: [Mindfulness] this term only 1279

#36 (sleep NEAR/3 (manag* or program* or regulat* or therap*)):ti,ab,kw (Word variations have been searched) 4225

#37 MeSH descriptor: [Adaptation, Psychological] this term only 4423

#38 (coping skill*):ti,ab,kw (Word variations have been searched) 2921

#39 #20 or #21 or #22 or #23 or #24 or #25 or #26 or #27 or #28 or #29 or #30 or #31 or #32 or #33 or #34 or #35 or #36 or #37 or #38 141634

#40 #4 and #19 and #39 1337

#41 MeSH descriptor: [Aged] this term only 219901

#42 MeSH descriptor: [Geriatrics] this term only 213

#43 MeSH descriptor: [Geriatric Assessment] this term only 1582

#44 (gerontol* or ageing or aging or elder* or geriatric* or senior* or "old age*" or "older person*" or "older people" or "older adult" or "late* life" or "very old" or "oldest old"):ti,ab,kw (Word variations have been searched) 787921

#45 #41 or #42 or #43 or #44 787921

#46 #40 and #45 835

#47 ((cancer NEAR/2 pain) NOT ("non cancer pain" or "noncancer pain")):ti,ab,kw (Word variations have been searched) 3130

#48 #46 NOT #47 with Publication Year from 2020 to 2022, in Trials 137

**Appendix 3 Details of data extraction**

We extracted the following data items from each included study:

- Study characteristics including location, study citation, eligibility criteria, sample size, type of RCT, follow-up duration and frequency, outcomes measures used, control/comparator arm(s) description;
- Characteristics of participants including age, sex, pain duration and intensity, comorbidities, living status, relationship status, ethnicity/race, ADL restriction due to pain, mood, frailty status;
- Experimental intervention(s) including intervention content, theoretical/conceptual framework, mechanism of change, behaviour change techniques used, pain dimensions addressed, mode of delivery, intervention time length, resources, expected input from participants, planned and actual delivered intervention, participants' engagement;
- Study results, adverse events, study authors' conclusions.

**Appendix 4 Characteristics of participants**

Four studies found that the participants' physical function mean score was lower than the midpoint of the measurement scale [2-5]. Similarly, the participants' mobility mean score in Tse *et al*. [6] suggested that they were independent in basic ADLs. Yet the mean time for performing four common ADLs was slightly longer than healthy people's normative time in Jessep *et al*. [4], and about double the time in Hurley *et al*. [3]. Two studies recruited veterans with similar age, sex, and ethnicity characteristics: Goode *et al*. [7] required the participants to be able to complete a home exercise programme intervention safely and hence this may explain their mean score was better than those in Hausmann *et al*. [8], whose participants self-assessed their function, with a mean score around the 75^th^ percentile of scores (higher score is worse), suggesting participants’ self-perceived physical function was poor. Participants in Janevic *et al*. [9] also scored lower than the mean of the general population.

Mean pain interference scores of participants in one study were lower than midpoint of the scale [10], whilst those in four other studies scored about the midpoint [11] [12] [13] [14], and higher than midpoint of the score of the general population in two studies [7] [9]. Ersek *et al*. 2003 and 2008 [12, 13] had the oldest mean age of participants of over 80 years, but interference was scored around midpoint of the scales, even though the inclusion criteria required some pain interference in regular activities and the pain was not limited to any specific pain site. Weiner *et al*. [15] reported how much pain interfered with daily activities in the prior week. Over 80% of participants reported that their day-to-day activities and working around the home were quite a bit or very much interfered by pain, and over half of participants also reported that social activities and household chores were affected quite a bit or very much.

Pain-related disability was scored lower than midpoint of the scales (i.e., better than midpoint) in four studies [16-19]; scored around the midpoint or the normative value of similar population groups with pain conditions in another four studies [20-23]; and higher than midpoint (i.e. worse) in one study [24]. The mean scores found in these studies did not appear to be related to the study eligibility criteria.

**Appendix 5 CASP assessment**

We used CASP RCT Standard Checklist to critically appraise the included studies and to inform consideration of the relevance and transferability of the results to older adults with persistent pain and frailty (Table). All the studies addressed a clearly focused research question (Q)1. At study selection, we only included studies reporting the assignment method as randomised. However, we did not have sufficient information about the assignment sequence generation and allocation concealment methods for eight studies to answer "yes" to Q 2. Dropouts were common but some studies used imputation or modelling methods to account for the missing data, so only ten studies did not account for all the randomised participants in the analyses (Q3).

Blinding the intervention providers (who delivered the interventions) and participants was usually unfeasible. Mostly, we did not have sufficient information about whether they were blinded or blinded securely, or whether the personnel analysing the outcomes were blinded (Q4). Some studies only reported the baseline characteristics of participants who completed all follow-ups; hence we could not determine if the study arms were similar at baseline for those studies (Q5). We judged that the participants in all arms in each study received the same level of care except the intervention (Q6).

We judged that all studies reported the results comprehensively (Q7) and with precision of the estimate of the intervention or treatment effect (Q8) [except Baird and Sands?]. When considering whether the benefits of an intervention outweighed the harms and costs, we considered whether there was any evidence of benefits (i.e., at least showing some statistically significant difference between groups in one outcome result), or any potential to provide benefits (i.e., improvement within the intervention arm after receiving the intervention). Only four among 1872 participants in all eligible intervention arms experienced non-life-threatening adverse events from physical exercises, so we considered all the interventions to be safe. Twenty-five studies showed some evidence of benefits ("yes"). That is, if there was any statistically significant improvement in outcome in the experimental intervention arm compared to the control arm, whatever the outcome and follow-up time-point were, we inferred that there may be benefits and hence potentially cost-effectiveness too. We inferred that the intervention may potentially have provided benefits if there was only within-group benefit in the intervention arm (any outcome improvement during or after receiving the intervention), answering "can't tell" (Q9). We judged that most of the studies' sample populations were broadly similar to our target population (mean age of 65 or above with frailty) according to the reported comorbidities and physical function (Q10). The exceptions were the studies of Broderick *et al*. [25] in which participants were unlikely to representative of the general older population because most of them had college or higher education qualifications; and Kwok *et al*. [26] who commented that the sample was relatively healthy thus the findings may not be generalisable to older adults who are less healthy and in more severe pain in other body parts.

The potential to provide benefits and the resources reallocation for delivering the experimental intervention were our main considerations for whether the intervention would provide greater value to people in typical pain management care in the United Kingdom (Q11). Ten studies showed potential to provide benefits in outcomes and the resources required for delivering the intervention were similar to standard PMP available, as described by the BPS [27]; but we were uncertain about the interventions in 16 studies due to the uncertainty about the resources required for implementing and delivering the intervention.

**Tables**

**Table 1: CASP assessment**

|  | Section A: Is the basic study design valid for a randomised controlled trial? | | | Section B: Was the study methodologically sound? | | | | | | Section C: What are the results? | | | Section D: Will the results help locally? | |
| --- | --- | --- | --- | --- | --- | --- | --- | --- | --- | --- | --- | --- | --- | --- |
|  | 1.Did the study address a clearly focused research question? | 2.Was the assignment of participants to interventions randomised? | 3.Were all participants who entered the study accounted for at its conclusion? | 4a.Were the participants ‘blind’ to intervention they were given? | 4b.Were the investigators ‘blind’ to the intervention they were giving to participants? | 4c.Were the people assessing analysing outcomes blinded? | 5.Were the study groups similar at the start of the randomised controlled trial? | 6.Apart from the experimental intervention, did each study group receive the same level of care (that is, were they treated equally)? | 7.Were the effects of intervention reported comprehensively? | | 8.Was the precision of the estimate of the intervention or treatment effect reported? | 9.Do the benefits of the experimental intervention outweigh the harms and costs? | 10.Can the results be applied to your local population in your context? | 11.Would the experimental intervention provide greater value to the people in your care than any of the existing interventions? |
| **Andersson et al. [28]** | ● | ● | ● | ? | ? | ? | ? | ● | ● | | ● | ● | ● | ? |
| **Baird and Sands [29]** | ● | ? |  | ? |  | ? | ● | ● | ● | |  | ● | ● | ● |
| **Bearne et al. [2]** | ● | ● |  |  |  | ? | ● | ● | ● | | ● | ? | ● |  |
| **Berman et al. [11]** | ● | ● |  |  |  | ? | ● | ● | ● | | ● | ● | ● | ● |
| **Broderick et al. [25]** | ● | ● | ● |  |  |  | ● | ● | ● | | ● | ● | ? | ? |
| **Carmody et al. [30]** | ● | ? | ● |  |  |  | ● | ● | ● | | ● | ? | ● |  |
| **Cheng et al. [20]** | ● | ● |  | ● |  | ? | ? | ● | ● | | ● | ● | ● | ? |
| **Costanti and Romiti [16]** | ● | ? |  | ? |  | ? | ● | ● | ● | | ● | ? | ● |  |
| **da Silva et al. [17]** | ● | ? |  |  |  | ● | ? | ● | ● | | ● | ● | ● |  |
| **Ersek et al. [12]** | ● | ● | ● |  |  | ? | ● | ● | ● | | ● | ? | ● |  |
| **Ersek et al. [13]** | ● | ● | ● |  | ? | ? |  | ● | ● | | ● | ? | ● |  |
| **Fanning et al. [14]** | ● | ● | ● | ? |  | ? | ● | ● | ● | | ● | ● | ● | ? |
| **Goode et al. [7]** | ● | ● | ● |  |  | ? | ● | ● | ● | | ● | ? | ● |  |
| **Haas et al. [18]** | ● | ● | ● |  |  | ? | ● | ● | ● | | ● | ● | ● | ● |
| **Hasegawa et al. [31]** | ● | ● | ● |  |  | ● | ● | ● | ● | | ● | ● | ● | ? |
| **Hausmann et al. [8]** | ● | ● | ● | ● |  | ? | ● | ● | ● | | ● | ● | ● | ● |
| **Hurley et al. [3]** | ● | ? | ● |  |  | ? | ● | ● | ● | | ● | ● | ● | ● |
| **Janevic et al. [9]** | ● | ? |  |  |  | ? | ? | ● | ● | | ● | ● | ● | ● |
| **Jessep et al. [4]** | ● | ● |  |  |  | ? | ● | ● | ● | | ● | ● | ● | ● |
| **Kwok et al. [26]** | ● | ? | ● | ● |  | ? | ? | ● | ● | | ● | ● | ? | ? |
| **Li et al. [10]** | ● | ● | ● | ● |  | ? | ● | ● | ● | | ● | ● | ● | ? |
| **Morone et al. [21]** | ● | ● | ● | ? |  | ? | ● | ● | ● | | ● | ● | ● | ? |
| **Morone et al. [22]** | ● | ● |  | ? |  | ? | ? | ● | ● | | ● | ? | ● |  |
| **Morone et al. [24]** | ● | ● | ● |  |  | ? | ● | ● | ● | | ● | ● | ● | ? |
| **Nicholas et al. [23]** | ● | ● | ● |  |  | ● | ● | ● | ● | | ● | ● | ● | ● |
| **Rini et al. [5]** | ● | ● | ● |  |  | ? | ● | ● | ● | | ● | ● | ● | ? |
| **Tse et al. [6]** | ● | ● | ● | ● |  | ? | ● | ● | ● | | ● | ● | ● | ? |
| **Vitiello et al. [19] CBT-PI** | ● | ● | ● |  |  | ? |  | ● | ● | | ● | ● | ● | ? |
| **Vitiello et al. [19] CBT-P** | ● | ● | ● |  |  | ? |  | ● | ● | | ● | ● | ● | ? |
| **Walsh et al. [32]** | ● | ? |  |  |  | ? | ● | ● | ● | | ● | ● | ● | ● |
| **Weiner et al. [15]** | ● | ● | ● |  |  | ● |  |  | ● | | ● | ● | ● | ? |
| **Yarns et al. [33] EAET** | ● | ● | ● |  | ● | ? | ● | ● | ● | | ● | ● | ● | ? |
| **Yarns et al. [33] CBT** | ● | ● | ● |  | ● | ? | ● | ● | ● | | ● | ? | ● | ? |
| ● = Yes; blank cell = No; ? = Can’t tell | | | | | | | | | | | | | | |

**Table 2. Characteristics of included studies**

**Andersson (2012), Sweden [28]**

| **Eligibility criteria** | Inclusion criteria:  • Age over 65 years;  • Chronic back and/or neck pain with no radiation to arms or legs, pain duration > 6 months;  • Having a medical ﬁle at the hospital, being able to walk stairs;  • Being able to attend group meetings;  • A physician screened all medical ﬁles before inclusion. Participants were interviewed using a structured interview and their medical ﬁles were checked by a physician. |
| --- | --- |
|  | Exclusion criteria:  • A history of dementia or cognitive impairment or those who were unable to understand the study procedures and the consent form were excluded. |
| **Sample size (assigned to intervention and control arms)** | IG: n=11  CG: n=10 |
| **Participant demographic characteristics (age, gender, ethnicity)** | Age, mean (SD), years:  Overall (n=21): 72.0 (4.6)  Overall range: 65-82 |
|  | Gender (Female), Number (%):  Overall (n= 21): 16 (76%) |
|  | Ethnicity: Not reported |
| **Participant health status (Pain duration, Pain intensity, Comorbidities, ADL restriction due to pain, mood)** | Pain duration:  Duration of pain (years), mean (SD), range (n=21): 15.8 (14.5), 0.5-48 |
|  | Pain intensity: Pain (VAS 0-100), mean (SD):  IG (n=11): 37.9 (9.6)  CG (n=10): 33.2 (23.0) |
|  | Comorbidities: Not reported |
|  | ADL (or physical function): Not reported |
|  | Mood:  HADS anxiety, mean (SD)  IG (n=11): 8.2 (3.9)  CG (n=10): 10.0 (5.3)  HADS depression, mean (SD)  IG (n=11): 4.6 (2.8)  CG (n=10): 5.6 (4.8) |
| **Study design (RCT type)** | Parallel-group RCT |
| **Intervention description (name, duration)** | Name: Brief cognitive-behavioural group treatment |
|  | Duration: 6 weeks |
| **Control arm description** | Waiting list |
| **Data collection time points** | Baseline (1 week before intervention), 8 weeks (1 week after intervention) |
| **Outcome measures for treatment effects** | Multidimensional Pain Inventory  Visual analogue scale (0 -100)  Pain and Impairment Relationship Scale (PAIRS)  HADS - anxiety, depression subscales  Anxiety and Sensitivity Index (ASI)  Quality of Life Inventory (QOLI)  Coping Strategies Questionnaire (CSQ) |
| IG=intervention group; CG=control group; SD=standard deviation; VAS=visual analogue scale; ADL=activities of daily living; HADS=hospital anxiety and depression scale; RCT=randomised controlled trial | |

**Baird (2004), USA [29]**

| **Eligibility criteria** | Inclusion criteria:  • Women over 65 years of age with a diagnosis of OA and joint pain. |
| --- | --- |
|  | Exclusion criteria:  • Individuals with a history of dementia or cognitive impairment or who were unable to understand the study procedures and the consent form. |
| **Sample size (assigned to intervention and control arms)** | IG: n=18  CG: n=10 |
| **Participant demographic characteristics (age, gender, ethnicity)** | Age, mean (SD), years:  IG (n=17): 72.06 (7.32)  CG (n=10): 74.80 (9.75)  Overall range: 65-93 |
|  | Gender (Female), Number (%):  Overall (n=28): 100% |
|  | Ethnicity: Not reported |
| **Participant health status (Pain duration, Pain intensity, Comorbidities, ADL restriction due to pain, mood)** | Pain duration: Not reported |
|  | Pain intensity:  Perceived pain (Arthritis Impact Measures - AIMS2), mean, reported in graph only  IG (n=17): ~3.3  CG (n=10): ~3.6 |
|  | Comorbidities:  IG (n=17):  Fibromyalgia: 2 (11%)  Polymyalgia: 3 (17%)  Other unspecified arthritis: 1 (6%)  Other medical conditions: 15 (83%)  CG (n=10):  Fibromyalgia: 1 (7%)  Polymyalgia: 0 (0%)  Other unspecified arthritis: 1 (7%)  Other medical conditions: 10 (67%) |
|  | ADL (or physical function): Not reported |
|  | Mood: Not reported |
| **Study design (RCT type)** | Parallel-group RCT |
| **Intervention description (name, duration)** | Name: Guided Imagery (GI) with Progressive Muscle Relaxation (PMR) |
|  | Duration: 12 weeks |
| **Control arm description** | Standard care + Journaling 3 times a week about arthritis symptoms of arthritis and managing arthritis. |
| **Data collection time points** | Baseline, 12 weeks |
| **Outcome measures for treatment effects** | Arthritis Impact Measures (AIMS2): questions about arthritis pain, walking and bending. |
| IG=intervention group; CG=control group; SD=standard deviation; ~=approximately; ADL=activities of daily living; RCT-randomised controlled trial | |

**Bearne (2011), UK [2]**

| **Eligibility criteria** | Inclusion criteria:  • Chronic hip pain of more than six months’ duration were recruited from two general practitioner practices in the south of England  • 50 years of age or older with a clinical diagnosis of hip osteoarthritis |
| --- | --- |
|  | Exclusion criteria:  • Had received physiotherapy for hip pain within the past six months;  • Had primary pain from other joints (e.g. back, knees or ankles) which interfered with assessment;  • Had unstable co‐existing medical problems (e.g. cardiovascular, respiratory or neurological disorders);  • Had received an intra‐articular injection to the hip within six months of study commencement;  • Were currently taking systemic steroids;  • Were unable or unwilling to exercise or unable or unwilling to give informed consent. |
| **Sample size (assigned to intervention and control arms)** | IG: n=24  CG: n=24 |
| **Participant demographic characteristics (Age, Gender, Ethnicity)** | Age, mean (SD), years:  IG (n=24): 65  CG (n=24): 67  Overall range: 52-78 |
|  | Gender (Female), Number (%):  IG (n=24): 15 (63%)  CG (n=24): 19 (79%) |
|  | Ethnicity: Not reported |
| **Participant health status (Pain duration, Pain intensity, Comorbidities, ADL restriction due to pain, mood)** | Pain duration:  Duration of hip pain (years), mean (range)  IG (n=24): 4.4 (1-12)  CG (n=24): 5.6 (1-40) |
|  | Pain intensity: Pain (WOMAC-pain), mean (SD)  IG (n=24): 5.0 (2.65)  CG (n=24): 5.2 (4.22) |
|  | Comorbidities: Not reported |
|  | ADL (or physical function):  Physical function (WOMAC-function), mean (SD)  IG (n=24): 14.3 (9.0)  CG (n=24): 17.3 (12.5) |
|  | Mood:  HADS anxiety, mean (SD)  IG (n=24): 5.0 (2.6)  CG (n=24): 4.1 (2.6)  HADS depression, mean (SD)  IG (n=24): 3.04 (2.3)  CG (n=24): 2.88 (2.8) |
| **Study design (RCT type)** | Parallel-group RCT |
| **Intervention description (name, duration)** | Name: Exercise‐Based Rehabilitation Programme for Chronic Hip Pain |
|  | Duration: 5 weeks |
| **Control arm description** | Usual care |
| **Data collection time points** | Baseline, 6 weeks, 6 months |
| **Outcome measures for treatment effects** | WOMAC: Total score, pain subscale, function subscale  HADS - anxiety, depression subscales  Aggregate functional performance time (AFPT]  Arthritis self‐efﬁcacy scale |
| IG=intervention group; CG=control group; SD=standard deviation; WOMAC=Western Ontario and McMaster Universities Arthritis Index; ADL=activities of daily living; HADS=hospital anxiety and depression scale; RCT=randomised controlled trial | |

**Berman (2009), USA[11]**

| **Eligibility criteria** | Inclusion criteria:  • Aged 55 years or older;  • Reported at least 1 day in the previous 30 days when pain made it difﬁcult to do usual activities and/or at least moderate levels of pain on average (a minimum score of 3 on a scale of 0 to 10);  • Had basic familiarity with computers;  • Could read and understand English. |
| --- | --- |
|  | Exclusion criteria: Not reported |
| **Sample size (assigned to intervention and control arms)** | IG: n=52  CG: n=37 |
| **Participant demographic characteristics (Age, Gender, Ethnicity)** | Age, mean (SD), years:  IG (n=41): 64.3  CG (n=37): 67.5  Overall range: 55-91 |
|  | Gender (Female), Number (%):  IG (n=41): 36 (87.8)  CG (n=37): 32 (86.5) |
|  | Ethnicity:  Race/ethnicity, n (%):  IG (n=41):  Caucasian (not Hispanic): 22 (53.7%)  African American (not Hispanic): 18 (43.9%)  Hispanic/Latino: 1 (2.4%)  Other: 0 (0%)  CG (n=37):  Caucasian (not Hispanic): 17 (45.9%)  African American (not Hispanic): 17 (45.9%)  Hispanic/Latino: 2 (5.4%)  Other: 1 (2.7%) |
| **Participant health status (Pain duration, Pain intensity, Comorbidities, ADL restriction due to pain, mood)** | Pain duration: Not reported |
|  | Pain intensity:  Pain intensity (BPI, average mean of 4 intensity items), mean (SD)  IG (n=41): 4.54 (2.08)  CG (n=37): 5.01 (1.83) |
|  | Comorbidities: Not reported |
|  | ADL (or physical function):  Pain interference (BPI), mean (SD)  IG (n=41): 4.21 (2.74)  CG (n=37): 3.95 (2.16) |
|  | Mood:  CESD-10, mean (SD)  IG (n=41): 10.06 (6.25)  CG (n=37): 9.70 (5.18)  Anxiety (STAI Y-6) (6-24), mean (SD):  IG (n=41): 11.54 (4.22)  CG (n=37): 10.41 (3.53) |
| **Study design (RCT type)** | Parallel-group RCT |
| **Intervention description (name, duration)** | Name: Online mind-body self-care techniques pain management intervention |
|  | Duration: 6 weeks |
| **Control arm description** | Waiting list |
| **Data collection time points** | Baseline, 6 weeks |
| **Outcome measures for treatment effects** | Brief Pain Inventory-Short Form (BPI)  Pain interference subscale of the BPI  Pain Self-efﬁcacy Questionnaire (PSEQ)  Center for Epidemiologic Studies Short Depression Scale (CES-D 10)  6-item State-Trait Anxiety Inventory (STAI-6)  Pain Awareness Questionnaire (PAQ) |
| IG=intervention group; CG=control group; SD=standard deviation; ADL=activities of daily living; BPI= Brief Pain Inventory-Short Form; CESD-10=Center for Epidemiologic Studies-Depression Scale; STAI Y-6, State-  Trait Anxiety Inventory Form Y and 6-item; RCT=randomised controlled trial; RCT=randomised controlled trial | |

**Broderick (2014), USA [25, 34]**

| **Eligibility criteria** | Inclusion criteria:  • Physician-confirmed diagnosis of hip or knee OA;  • 21 years of age or older;  • Usual pain 4 on a 10-point scale for a duration of at least 6 months;  • Ability to read, write, and understand English;  • Ability to attend 10 treatment sessions at the doctor’s office if randomized to treatment;  • No cognitive/ mental impairment that would interfere with participation,  • No expected joint replacement surgery in the next 2 years. |
| --- | --- |
|  | Exclusion criteria: Not reported separately. |
| **Sample size (assigned to intervention and control arms)** | IG: n=129  CG: n=127 |
| **Participant demographic characteristics (Age, Gender, Ethnicity)** | Age, mean (SD), years:  IG (n=129): 68.00 (8.67)  CG (n=128): 66.37 (10.26) |
|  | Gender (Female), Number (%):  IG (n=129): 74.4%  CG (n=128): 78.9% |
|  | Ethnicity:  White race, %:  IG (n=129): 87.6%  CG (n=128): 85.9% |
| **Participant health status (Pain duration, Pain intensity, Comorbidities, ADL restriction due to pain, mood)** | Pain duration:  Years with osteoarthritis, mean (SD)  IG (n=128): 13.95 (10.63)  CG (n=121): 13.59 (9.09) |
|  | Pain intensity: Not reported |
|  | Comorbidities: Not reported |
|  | ADL (or physical function): Not reported |
|  | Mood:  Receiving treatment for psychiatric disorder, %  IG (n=128): 16.4%  CG (n=126): 15.1% |
| **Study design (RCT type)** | Parallel-group RCT |
| **Intervention description (name, duration)** | Name: Pain coping skills training (PCST) |
|  | Duration: 10 weeks |
| **Control arm description** | Usual care for OA (which includes providing with an OA informational brochure from the Arthritis Foundation and information on programs (support groups, arthritis education, and aquatic exercise classes) offered in the community). |
| **Data collection time points** | Baseline, 10 weeks, 6 months, 12 months |
| **Outcome measures for treatment effects** | Brief Pain Inventory (BPI)  WOMAC  Beck Depression Inventory (BDI)  Quality of Life Scale  Arthritis Self-Efficacy Scale  Coping Strategies Questionnaire (CSQ)  Arthritis Impact Measurement Scales (AIMS2)  Brief Fatigue Inventory (BFI) |
| IG=intervention group; CG=control group: SD=standard deviation; OA=osteoarthritis; ADL=activities of daily living; RCT=randomised controlled trial | |

**Carmody (2013), USA [30]**

| **Eligibility criteria** | Inclusion criteria:  • Military veterans enrolled in a VA primary-care clinic;  • Ages 55 or older;  • With documented chronic pain for at least one year;  • Having access to a telephone;  • Pain conditions must have been stable, with no clear indication for specific medical/surgical interventions. |
| --- | --- |
|  | Exclusion criteria:  • Were psychotic, cognitively impaired, at significant risk for suicide (history of multiple suicide attempts or actively suicidal), and currently abusing or dependent on alcohol or other drugs, including prescribed opioid pain medications. |
| **Sample size (assigned to intervention and control arms)** | IG: n=50  CG: n=51 |
| **Participant demographic characteristics (Age, Gender, Ethnicity)** | Age, mean (SD), years:  IG (n=48): 66 (9)  CG (n=50): 69 (10) |
|  | Gender (Female), Number (%):  IG (n=48): 2 (4%)  CG (n=50): 1 (2%) |
|  | Ethnicity:  White, number (%)  IG (n=48): 33 (69%)  CG (n=50): 34 (72% |
| **Participant health status (Pain duration, Pain intensity, Comorbidities, ADL restriction due to pain, mood)** | Pain duration:  Duration of pain (years), mean (SD)  IG (n=48): 18 (18)  CG (n=50): 17 (15) |
|  | Pain intensity:  Pain intensity (bespoke) (0-5), mean (SD)  IG (n=48): 4.3 (1.1)  CG (n=50): 4.1 (1.2) |
|  | Comorbidities: Not reported |
|  | ADL (or physical function):  Disabled, number (%)  IG (n=48): 20 (42%)  CG (n=50): 21 (42%) |
|  | Mood:  Current major depression, number (%)  IG (n=48): 15 (31%)  CG (n=50): 11 (22%) |
| **Study design (RCT type)** | Parallel-group RCT |
| **Intervention description (name, duration)** | Name: Telephone-delivered cognitive-behavioural therapy (T-CBT) |
|  | Duration: 20 weeks |
| **Control arm description** | Telephone-delivered pain education (T-EDU) |
| **Data collection time points** | 10 weeks (midtreatment), 20 weeks (posttreatment), 32 weeks (3-month follow-up), and 46 weeks (6-month follow-up). |
| **Outcome measures for treatment effects** | Pain intensity (0 no pain to 5 incapacitating pain)  SF-12v2 - mental health summary score, physical health summary score  Pain Behaviour Checklist (PBCL)  Coping Strategies Questionnaire-Revised (CSQ-R) |
| VA=Veteran Affairs; IG=intervention group; CG=control group; SD=standard deviation; ADL=activities of daily living; RCT=randomised controlled trial; SF-12v2=Short Form 12 Health Survey Version 2 | |

**Cheng (2022), Hong Kong [20] [35]**

| **Eligibility criteria** | Inclusion criteria:  • Aged 60 years or over;  • Moderate chronic pain affecting bones, muscles, and joints in the previous 3 months operationalized as scoring ≥40 out of 100 points on the Pain Intensity subscale of the Chronic Pain Grade questionnaire (Von Korff et al., 1992);  • Mild depressive symptoms as indicated by a score of ≥4 on the CESD-10 (Andresen et al., 1994; Cheng & Chan, 2005; Radloff, 1977);  • Cantonese speaking;  • Having basic ability to read and write. |
| --- | --- |
|  | Exclusion criteria:  • Insufﬁcient ﬂuency in Cantonese;  • Possible cognitive impairment as suggested by a score of ≥3 on the Short Portable Mental Status Questionnaire (Pfeiffer, 1975);  • Any impairment in basic activities of daily living as indicated by a score of <21 on the OARS Multidimensional Functional Assessment Questionnaire (Fillenbaum & Smyer, 1981);  • Physical conditions affecting participation in intervention, including speech and hearing impairments. |
| **Sample size (assigned to intervention and control arms)** | IG: n=89 (9 clusters)  CG: n=89 (9 clusters) |
| **Participant demographic characteristics (Age, Gender, Ethnicity)** | Age, mean (SD), years:  IG (n=77): 74.38 (5.93)  CG (n=75): 72.35 (8.02) |
|  | Gender (Female), Number (%):  IG (n=77): 85.7%  CG (n=75): 76.0% |
|  | Ethnicity:  Hong Kong Chinese (n=178): 100% |
| **Participant health status (Pain duration, Pain intensity, Comorbidities, ADL restriction due to pain, mood)** | Pain duration:  Pain duration (years), mean (SD)  IG (n=77): 9.95 (8.46)  CG (n=75): 10.07 (8.48) |
|  | Pain intensity:  Pain intensity (GCPS), mean (SD)  IG (n=77): 65.41 (15.02)  CG (n=75): 61.78 (13.37) |
|  | Comorbidities:  Number of chronic illnesses, mean (SD)  IG (n=77): 3.13 (1.84)  CG (n=75): 3.28 (2.17) |
|  | ADL (or physical function):  Pain disability (GCPS), mean (SD)  IG (n=77): 50.65 (21.08)  CG (n=75): 45.47 (18.04) |
|  | Mood:  CESD-10, mean (SD)  IG (n=77): 12.21 (6.98)  CG (n=75): 10.96 (6.75)  SF-12 Mental component, mean (SD)  IG (n=77): 49.17 (9.40)  CG (n=75): 50.86 (8.59) |
| **Study design (RCT type)** | Cluster RCT |
| **Intervention description (name, duration)** | Name: A group intervention combining exercise and cognitive behavioural strategies (EC) |
|  | Duration: 10 weeks |
| **Control arm description** | Pain education programme (10 group sessions) |
| **Data collection time points** | Baseline (1 week before intervention), 12 weeks (1 week after intervention), 3 months, 6 months. |
| **Outcome measures for treatment effects** | Chronic Pain Grade questionnaire (GCPS)  Pain Catastrophizing Scale  Presence of Chronic Pain  CESD-10  Short Portable Mental Status Questionnaire  SF-12  Measure with Back Strength Dynamometer  OARS Multidimensional Functional Assessment Questionnaire  Chronic Pain Self-Efﬁcacy Scale  Brief Coping Index |
| CESD-10=10-item Center for Epidemiologic Studies-Depression Scale; OARS= Older Americans Resources and Services; IG=intervention group; CG=control group SD=standard deviation; ADL=activities of daily living; SF-12=12-Item Short Form Health Survey; GCPS= Graded Chronic Pain Scale; RCT=randomised controlled trial | |

**Costantino (2014), Italy [16]**

| **Eligibility criteria** | Inclusion criteria:  • Participants aged between 65 and 80 years;  • Diagnosis of chronic non-specific low back pain;  • Algic low back pain recurrence in the last three months. |
| --- | --- |
|  | Exclusion criteria:  • Presence of musculoskeletal disorders, severe heart failure or internal medicine pathologies that could interfere with moderate physical activity;  • Fever or infectious disease;  • Systemic inflammatory or rheumatologic diseases;  • Previous spinal surgery or a history of vertebral traumas/fractures;  • Instrumental physical therapies or physiotherapeutic therapies in the previous three months. |
| **Sample size (assigned to intervention and control arms)** | IG: n=28  CG: n=28 |
| **Participant demographic characteristics (Age, Gender, Ethnicity)** | Age, mean (SD), years:  IG (n=27): 73.63 (3.36)  CG (n=27): 73.30 (3.55)  Overall range: 66-78 |
|  | Gender (Female), Number (%):  IG (n=27): 48.2%  CG (n=27): 40.7% |
|  | Ethnicity: Not reported |
| **Participant health status (Pain duration, Pain intensity, Comorbidities, ADL restriction due to pain, mood)** | Pain duration:  Symptoms duration (months), mean (SD):  IG (n=27): 22.81 (7.01)  CG (n=27): 23.96 (7.67) |
|  | Pain intensity: Not reported |
|  | Comorbidities: Not reported |
|  | ADL (or physical function):  Physical disability (RMDQ), mean (SD)  IG (n=27): 10.22 (2.50)  CG (n=27): 9.59 (3.08) |
|  | Mood: Not reported. |
| **Study design (RCT type)** | Parallel-group RCT |
| **Intervention description (name, duration)** | Name: Back School programme (stretching and selective muscle reinforcement techniques) |
|  | Duration: 12 weeks |
| **Control arm description** | Hydrotherapy program |
| **Data collection time points** | Baseline, 12 weeks, 26 weeks |
| **Outcome measures for treatment effects** | RMDQ  SF-36 v2 (Not calculated using a recommended method) |
| IG=intervention group; CG=control group SD=standard deviation; ADL=activities of daily living; RMDQ=Roland Morris Disability Questionnaire; RCT=randomised controlled trial | |

**da Silva (2014), Brazil[17]**

| **Eligibility criteria** | Inclusion criteria:  • Both genders, aged between 50 and 80 years;  • Having chronic non-specific back pain. |
| --- | --- |
|  | Exclusion criteria:  • Suffering from back pain for less than six months or whose pain was due to a specific cause (e.g., disc herniation, fracture, spondylolisthesis);  • Received treatment for pain with another intervention at the time of the study or had any oncologic, neurologic, and/or rheumatologic diseases;  • Anyone who was unable to answer questionnaires. |
| **Sample size (assigned to intervention and control arms)** | IG: n=11  Educational lessons CG: n=10  Physical exercise CG: n=10  Waiting list CG: n=10 |
| **Participant demographic characteristics (Age, Gender, Ethnicity)** | Age, mean (SD), years:  IG (n=8): 69.6 (6.0)  Educational lessons CG (n=5): 73.2 (9.6)  Physical exercise CG (n=9): 70.1 (2.7)  Waiting list CG (n=9): 64.6 (8.5) |
|  | Gender (Female), Number (%):  IG (n=8): 100%  Educational lessons CG (n=5): 100%  Physical exercise CG (n=9): 100%  Waiting list CG (n=9): 88.9% |
|  | Ethnicity: Not reported |
| **Participant health status (Pain duration, Pain intensity, Comorbidities, ADL restriction due to pain, mood)** | Pain duration: Not reported |
|  | Pain intensity:  Pain intensity (VAS) (0-10), mean (SD)  IG (n=8): 6.0 (1.4)  Educational lessons CG (n=5): 5.8 (3.0)  Physical exercise CG (n=9): 5.7 (2.1)  Waiting list CG (n=9): 5.2 (1.7) |
|  | Comorbidities: Not reported |
|  | ADL (or physical function):  Physical disability (RMDQ) (0-24), mean (SD)  IG (n=8): 8.3 (4.3)  Educational lessons CG (n=5): 12.4 (3.2)  Physical exercise CG (n=9): 9.0 (6.8)  Waiting list CG (n=9): 7.0 (4.5) |
|  | Mood:  WHOQOL-BREF-psychological (range 6-30), mean (SD)  IG (n=8): 22.3 (1.9)  Educational lessons CG (n=5): 22.0 (1.4)  Physical exercise CG (n=9): 22.1 (1.7)  Waiting list CG (n=9): 21.4 (1.8) |
| **Study design (RCT type)** | Parallel-group RCT |
| **Intervention description (name, duration)** | Name: Back school (BS) programme |
|  | Duration: 5 weeks |
| **Control arm description** | 3 control arms:  Educational lessons  Physical exercise  Waitlist control |
| **Data collection time points** | Baseline, 5 weeks |
| **Outcome measures for treatment effects** | RMDQ  VAS  WHO quality of life questionnaire (WHOQOL-BREF) |
| IG=intervention group; CG=control group SD=standard deviation; VAS=visual analogue scale; ADL=activities of daily living; RMDQ=Roland Morris Disability Questionnaire; WHOQOL-BREF= abbreviated World Health Organization Quality of Life questionnaire | |

**Ersek (2003), USA [12]**

| **Eligibility criteria** | Inclusion criteria:  • Residency in one of the participating retirement facilities;  • Age 60 years or older;  • Pain >3 months duration that interfered with regular activities;  • Ability to read and complete study questionnaires;  • Ability to attend at least five group sessions. |
| --- | --- |
|  | Exclusion criteria:  • Current cancer requiring treatment;  • Self-reported consumption of more than two alcoholic drinks per day;  • No potential participants were excluded because of these criteria. |
| **Sample size (assigned to intervention and control arms)** | IG: n=22  CG: n=23 |
| **Participant demographic characteristics (Age, Gender, Ethnicity)** | Age, mean (SD), years:  IG (n=22): 83.6 (SD 5.0)  CG (n=23): 80.3 (SD 6.6)  Overall range: 65-94 |
|  | Gender (Female), Number (%):  IG (n=22): 20 (91%)  CG (n=23): 19 (83%) |
|  | Ethnicity:  IG (n=22):  Caucasian: 21 (95%)  Other: 1 (5%)  CG (n=23):  Caucasian: 17 (74%)  Other: 6 (26%) |
| **Participant health status (Pain duration, Pain intensity, Comorbidities, ADL restriction due to pain, mood)** | Pain duration: Not reported |
|  | Pain intensity: Not reported |
|  | Comorbidities:  The 3 most common self-reported pain conditions (n=45), %  Osteoarthritis (68%)  Old fractures (20%)  Neuralgia (15%)  Others included rheumatoid arthritis, fibromyalgia, and migraine. |
|  | ADL (or physical function):  Pain interfered (GCPS), mean (SD)  IG (n=22): 4.0 (2.3)  CG (n=23): 4.3 (2.9)  Days kept from usual activities by pain (past 90 days), mean (SD)  IG (n=22): 47.2 (44.2)  CG (n=23): 25.1 (37.8) |
|  | Mood:  Geriatric depression scale, mean (SD)  IG (n=22): 8.2 (4.8)  CG (n=23): 8.5 (6.1) |
| **Study design (RCT type)** | Parallel-group RCT |
| **Intervention description (name, duration)** | Name: Self-management group intervention |
|  | Duration: 8 weeks |
| **Control arm description** | Educational booklet group |
| **Data collection time points** | Baseline (1 week before intervention), 9 weeks, 5 months (3 months after intervention ended) |
| **Outcome measures for treatment effects** | Survey of Pain Attitudes (SOPA) – harm, control, medical cure subscales  Graded chronic pain scale (GCPS)  SF-36 physical functioning |
| IG=intervention group; CG=control group SD=standard deviation; ADL=activities of daily living; RCT=randomised controlled trial; SF-36=36-Item Short Form Health Survey | |

**Ersek (2008), USA [13] [36, 37]**

| **Eligibility criteria** | Inclusion criteria:  • Age 65 years or older;  • Pain lasting more than three months that interfered with daily activities, average pain in the past week greater than two on a 0-10 scale;  • Ability to complete study questionnaires, and ability to attend seven weekly sessions at the participant’s retirement facility. |
| --- | --- |
|  | Exclusion criteria:  • Active cancer, surgery within the past six months, and surgery planned in the next six months. |
| **Sample size (assigned to intervention and control arms)** | IG: n=133 (21 clusters)  CG: n=123 (22 clusters) |
| **Participant demographic characteristics (Age, Gender, Ethnicity)** | Age, mean (SD), years:  IG (n=133): 81.9 (6.3)  CG (n=123): 81.8 (6.7) |
|  | Gender (Female), Number (%):  IG (n=133): 87.2%  CG (n=123): 82.1% |
|  | Ethnicity:  Caucasian, %:  IG (n=133): 93.2%  CG (n=123): 93.5% |
| **Participant health status (Pain duration, Pain intensity, Comorbidities, ADL restriction due to pain, mood)** | Pain duration: Not reported |
|  | Pain intensity:  Pain Intensity (0-10), mean (SD)  IG (n=133): 5.4 (1.9)  CG (n=123): 5.4 (1.8) |
|  | Comorbidities:  Charlson comorbidity score, %  IG (n=133):  0: 43.1%  1-2: 40.8%  ≥3: 16.1%  CG (n=123):  0: 40.7%  1-2: 38.2%  ≥3: 21.1% |
|  | ADL (or physical function):  Pain Interference (BPI) (0-10), mean (SD):  IG (n=133): 4.2 (2.0)  CG (n=123): 4.5 (2.0) |
|  | Mood:  Geriatric Depression Scale (0-30), mean (SD)  IG (n=133): 11.1 (2.8)  CG (n=123): 11.0 (3.0) |
| **Study design (RCT type)** | Cluster RCT |
| **Intervention description (name, duration)** | Name: Pain self-management group (SMG) Intervention |
|  | Duration: 7 weeks |
| **Control arm description** | Educational book provided + 2 telephone calls to encourage reading and answering questions from participants |
| **Data collection time points** | Baseline, 7 weeks, 6 months, 12 months |
| **Outcome measures for treatment effects** | Roland Morris disability questionnaire (RMDQ)  Brief Pain Inventory (BPI)  Coping Strategies Questionnaire (CSQ) - catastrophizing subscale  Geriatric Depression Scale (GDS)  Arthritis Self-Efficacy Scale  Chronic Pain Coping Inventory (CPCI)  Medication use |
| IG=intervention group; CG=control group SD=standard deviation; ADL=activities of daily living; RCT=randomised controlled trial | |

**Fanning (2020), USA [14] [38, 39]**

| **Eligibility criteria** | Inclusion criteria:  • Aged 55-85 years of age with a body mass index (BMI) of 30-45 kg/m2;  • Were low active (i.e., did not participate in regular resistance training and/or more than 20min of aerobic exercise on 2 or more days per week in the previous 6 months);  • Weight stable (i.e., had not lost or gained more than 5% of their body weight in the previous 6 months);  • Had no contraindication to exercise.  • Participants were also required to own a personal smartphone;  • Had pain in at least two of the following sites on most days in the previous 3 months: back, neck, shoulders, hips, or knees.  • Those who participated in the ﬁrst phase of the study could not participate in the second phase. |
| --- | --- |
|  | Exclusion criteria:  • Unable to walk without assistive devices;  • Will have cognitive impairment as indicated by a Montreal Cognitive Assessment score of less than 22. |
| **Sample size (assigned to intervention and control arms)** | IG: n=15  CG: n=13 |
| **Participant demographic characteristics (Age, Gender, Ethnicity)** | Age, mean (SD), years:  IG (n=15): 70.12 (5.43)  CG (n=13): 70.32 (5.20) |
|  | Gender (Female), Number (%):  IG (n=15): 13 (86.7%)  CG (n=13): 9 (69.2%) |
|  | Ethnicity:  Race, number (%)  IG (n=15):  Black: 3 (20%)  White: 12 (80%)  CG(n=13):  Black: 2 (15.4%)  White: 11 (84.6%) |
| **Participant health status (Pain duration, Pain intensity, Comorbidities, ADL restriction due to pain, mood)** | Pain duration: Not reported |
|  | Pain intensity:  Pain intensity (PROMIS), mean (SD)  IG (n=15): 64.21 (3.85)  CG (n=13): 60.42 (6.12)  SF-36 bodily pain, mean (SD)  IG (n=15): 39.01 (4.56)  CG (n=13): 43.22 (4.38) |
|  | Comorbidities: Not reported |
|  | ADL (or physical function):  Pain interference (PROMIS), mean (SD)  IG (n=15): 59.92 (8.59)  CG(n=13): 58.80 (7.74) |
|  | Mood: Not reported |
| **Study design (RCT type)** | Parallel-group RCT |
| **Intervention description (name, duration)** | Name: Mobile Intervention to Reduce Pain and improve Health (MOPRH) - group-mediated intervention for delivery in the home via mHealth tools |
|  | Duration: 12 weeks |
| **Control arm description** | Waitlist |
| **Data collection time points** | Baseline, 12 weeks |
| **Outcome measures for treatment effects** | Pain Interference Scale  PROMIS Pain Intensity Scale  SF-36 - pain, physical functioning subscales  Satisfaction with physical functioning  Short Physical Performance Battery (SPPB)  Self-efficacy for walking  Number of transitions from sedentary behaviour (SB) to non- sedentary behaviour  Number of daily steps  Body Mass Index |
| IG=intervention group; CG=control group SD=standard deviation; ADL=activities of daily living; PROMIS= Patient-Reported Outcomes Measurement Information System; SF-36=36-Item Short Form Health Survey; RCT=randomised controlled trial | |

**Goode (2018), USA [7]**

| **Eligibility criteria** | Inclusion criteria:  • Self-reported having had lower back pain on most days for greater than 3 months;  • Were sedentary, defined as reporting less than 150 minutes of moderate PA per week;  • Could complete a 10-second semi-tandem stand and walk about 2.4 meters (8 feet) in 6.0 seconds, to safely complete a home exercise program;  • Self-reported not satisfied with their current state of functional ability, based on reporting “dissatisfied” with at least 1 aspect of physical function on the Satisfaction With Physical Function Scale;  • Could safely participate in the intervention, based on the physical therapist baseline examination and clinical expertise. |
| --- | --- |
|  | Exclusion criteria:  • Unilateral or bilateral sciatica; isolated coccyx pain (based on self-report at screening);  • Dementia, memory loss, or other significant cognitive impairment found in the medical record;  • Movement or motor neuron disorders (eg, Parkinson disease, multiple sclerosis, amyotrophic lateral sclerosis);  • Rheumatoid arthritis, fibromyalgia, or other systemic rheumatic disease;  • Hospitalization for a stroke, myocardial infarction, heart failure, or coronary artery revascularization in the past 3 months;  • Significant hearing impairment without accommodations (must be able to talk on the telephone)  • Psychosis or current, uncontrolled substance abuse disorder;  • Any other health conditions determined by the study team to be contraindications to performing mild to moderate home exercises. |
| **Sample size (assigned to intervention and control arms)** | IG: n=20  Physical activity CG: n=20  Waitlist CG: n=20 |
| **Participant demographic characteristics (Age, Gender, Ethnicity)** | Age, mean (SD), years:  IG (n=20): 69.5 (4.0)  Physical activity CG (n=20): 69.6 (3.5)  Waitlist CG (n=20): 71.9 (6.5) |
|  | Gender (Female), Number (%):  IG (n=20): 2 (10%)  Physical activity CG (n=20): 1 (5%)  Waitlist CG (n=20): 1 (5%) |
|  | Ethnicity:  Race, number (%)  IG (n=20):  American Indian: 0 (0%)  Black/African American: 11 (55.0%)  White: 9 (45%)  Physical activity CG (n=20):  American Indian: 1 (5%)  Black/African American: 11 (55%)  White: 8 (40%)  Waitlist CG (n=20):  American Indian: 0 (0%)  Black/African American: 8 (40%)  White: 11 (55%)  Missing: 1 (5%)  Ethnicity  IG (n=20):  Hispanic: 0 (0%)  Not Hispanic or Latino: 20 (100%)  Physical activity CG (n=20):  Hispanic: 1 (5%)  Not Hispanic or Latino: 19 (95%)  Waitlist CG (n=20):  Hispanic: 0 (0%)  Not Hispanic or Latino: 20 (100%) |
| **Participant health status (Pain duration, Pain intensity, Comorbidities, ADL restriction due to pain, mood)** | Pain duration:  Duration of symptoms, number of participants (%)  IG (n=20):  6 m-1 yr: 1 (5%)  1-5 yr: 2 (10%)  >5 yr: 17 (85%)  Physical activity CG (n=20):  6 m-1yr: 0 (0%)  1-5 yr: 3 (15%)  >5 yr: 17 (85%)  Waitlist CG (n=20):  6 m-1 yr: 2 (10%)  1-5 yr: 2 (10%)  >5 yr: 16 (80%) |
|  | Pain intensity: Not reported |
|  | Comorbidities: Not reported |
|  | ADL (or physical function):  Pain Interference (PROMIS) (1-20), mean (SD)  IG (n=20): 13.0 (4.4)  Physical activity CG (n=20): 13.5 (3.9)  Waitlist CG (n=20): 11.9 (3.6)  Physical function (PROMIS) (1-20), mean (SD)  IG (n=20): 12.3 (2.7)  Physical activity CG (n=20): 11.2 (3.8)  Waitlist CG (n=20): 12.8 (3.3) |
|  | Mood: Not reported |
| **Study design (RCT type)** | Parallel-group RCT |
| **Intervention description (name, duration)** | Name: Physical activity plus cognitive-behavioural therapy for pain (CBT-P) |
|  | Duration: 12 weeks |
| **Control arm description** | 2 control arms:  Telephone-supported physical activity only  Waitlist control |
| **Data collection time points** | Baseline, 12 weeks |
| **Outcome measures for treatment effects** | Roland Morris disability questionnaire (RMDQ)  Timed Up & Go Test (TUG)  Satisfaction with Physical Function  Patient-Specific Functional Scale (PSFS)  PROMIS Health Assessment Questionnaire  Coping Strategies Questionnaire (CSQ) |
| IG=intervention group; CG=control group SD=standard deviation; ADL=activities of daily living; yr=year; PROMIS= Patient-Reported Outcomes Measurement Information System; RCT=randomised controlled trial | |

**Haas (2005), USA [18] [40]**

| **Eligibility criteria** | Inclusion criteria:  • African American or white seniors;  • Aged 60 and over;  • Suffered from chronic LBP (3 months or longer);  • Had the ability to read and write English. |
| --- | --- |
|  | Exclusion criteria:  • Dementia, significant heart or respiratory illness, serious blood disorders;  • Participation in another intensive health promotion program within the last year  • Unwillingness to be randomized. |
| **Sample size (assigned to intervention and control arms)** | IG: n=60  CG: n=60 |
| **Participant demographic characteristics (Age, Gender, Ethnicity)** | Age, mean (SD), years:  IG (n=60): 78.6 (7.5)  CG (n=49): 75.5 (7.5) |
|  | Gender (Female), Number (%):  IG (n=60): 81.6%  CG (n=49): 87.8% |
|  | Ethnicity:  IG (n=60):  White: 81.6%  African American: 18.4%  CG (n=49):  White: 89.8%  African American: 10.2% |
| **Participant health status (Pain duration, Pain intensity, Comorbidities, ADL restriction due to pain, mood)** | Pain duration: Not reported |
|  | Pain intensity:  Pain (Modified Von Korff scales, 0-100), mean (SD)  IG (n=60): 48.3 (25.7)  CG (n=49): 49.2 (22.4) |
|  | Comorbidities: Not reported |
|  | ADL (or physical function):  Pain-related disability (Modified Von Korff scales), mean (SD)  IG (n=60): 44.4 (28.7)  CG (n=49): 39.8 (24.5)  Disability days (last 2 weeks), mean (SD)  IG (n=60): 4.1 (4.6)  CG (n=49): 4.1 (4.9) |
|  | Mood:  SF-36 emotional well-being, mean (SD)  IG (n=60): 67.6 (20.1)  CG (n=49): 69.2 (16.4)  SF-36 depression screen (positive), %  IG (n=60): 16.7%  CG (n=49): 12.2% |
| **Study design (RCT type)** | Parallel-group RCT |
| **Intervention description (name, duration)** | Name: Chronic Disease Self-Management Program (CDSMP) for chronic low back pain (LBP |
|  | Duration: 24 weeks (6 weeks intervention, and telephone calls support every 2 weeks for 24 weeks) |
| **Control arm description** | Waitlist |
| **Data collection time points** | Baseline, 6 months |
| **Outcome measures for treatment effects** | Modified Von Korff scale – disability, pain scales  SF-36 - General health, Emotional wellbeing, Energy fatigue  Arthritis Self-Efficacy Scale  Self-care attitudes (responsibility, energy/time) |
| IG=intervention group; CG=control group SD=standard deviation; ADL=activities of daily living; SF-36=36-Item Short Form Health Survey; RCT=randomised controlled trial | |

**Hasegawa (2021), Japan [31, 41]**

| **Eligibility criteria** | Inclusion criteria:  • Chronic lower back pain for ≥6months;  • Aged over 65 years old;  • Independence in activities of daily life. |
| --- | --- |
|  | Exclusion criteria:  • Suspected cognitive decline (Mini Mental State Examination 23 points or less);  • Receiving individual physical therapy sessions;  • No consent to participate. |
| **Sample size (assigned to intervention and control arms)** | IG: n=22  CG: n=21 |
| **Participant demographic characteristics (Age, Gender, Ethnicity)** | Age, mean (SD), years:  IG (n=22): 77.9 (7.0)  CG (n=21): 79.9 (5.9) |
|  | Gender (Female), Number (%):  IG (n=21): 4 (19.1%)  CG (n=21): 3 (14.3%) |
|  | Ethnicity: Presumably all Japanese |
| **Participant health status (Pain duration, Pain intensity, Comorbidities, ADL restriction due to pain, mood)** | Pain duration: Not reported |
|  | Pain intensity (NRS), mean (SD):  IG (n=22): 4.7 (1.4)  CG (n=21): 5.7 (1.4) |
|  | Comorbidities: Not reported |
|  | ADL (or physical function):  ADL (Barthel Index), mean (SD):  IG (n=22): 97.5 (3.4)  CG (n=21): 94.2 (6.4) |
|  | Mood:  HADS anxiety, mean (SD)  IG (n=22): 6.7 (2.3)  CG (n=21): 6.4 (1.8)  HADS depression, mean (SD)  IG (n=22): 7.4 (2.2)  CG (n=21): 6.6 (3.5) |
| **Study design (RCT type)** | Parallel-group, crossover RCT |
| **Intervention description (name, duration)** | Name: Attentional bias modification (ABM) + standard intervention |
|  | Duration: 12 weeks (before crossover only) |
| **Control arm description** | Waitlist (ABM Trailing arm before crossover) + standard intervention |
| **Data collection time points** | (Before crossover only) Baseline, 12 weeks |
| **Outcome measures for treatment effects** | Numerical Rating Scale (NPR)  Pain catastrophizing scale  HADS - anxiety, depression subscales  EQ-5D-3L  Somatic Symptom Scale-8  Timed Up & Go Test (TUG)  30-second chair-stand test  Fear-Avoidance Beliefs Questionnaire (FABQ-J) |
| IG=intervention group; CG=control group SD=standard deviation; ADL=activities of daily living; NRS= Numerical Rating Scale; HADS=hospital anxiety and depression scale; RCT=randomised controlled trial; EQ-5D=EuroQol 5 Dimensions | |

**Hausmann (2017), USA [8]**

| **Eligibility criteria** | Inclusion criteria:  • Veterans aged 50 years or older;  • Receive primary care from the VA;  • Report having physician diagnosed OA; have frequent knee or hip pain; and rate their pain level as 4 or greater on a 0-10 scale. |
| --- | --- |
|  | Exclusion criteria:  • Inflammatory arthritis or low back pain;  • Problems with serious illness, memory, hearing, or eyesight that would prevent them from completing the study;  • Inability to receive study related telephone calls;  • Inability to complete simple writing activities without assistance. |
| **Sample size (assigned to intervention and control arms)** | IG: n=21  CG: n=21 |
| **Participant demographic characteristics (Age, Gender, Ethnicity)** | Age, mean (SD), years:  IG (n=21): 69.2 (11.3)  CG (n=21): 65.7 (9.1) |
|  | Gender (Female), Number (%):  IG (n=21): 4 (19.1%)  CG (n=21): 3 (14.3%) |
|  | Ethnicity:  Race, number (%)  IG (n=21):  Black/African American: 8 (38.1%)  White: 13 (61.9%)  CG (n=21):  Black/African American: 10 (47.6%)  White: 11 (52.4%) |
| **Participant health status (Pain duration, Pain intensity, Comorbidities, ADL restriction due to pain, mood)** | Pain duration: Not reported |
|  | Pain intensity:  WOMAC Pain (0-100), mean (SD)  IG (n=21): 51.4 (15.5)  CG (n=21): 51.4 (18.0) |
|  | Comorbidities:  Charlson comorbidity index, mean (SD):  IG (n=21): 3.2 (2.4)  CG (n=21): 4.1 (3.1) |
|  | ADL (or physical function):  Physical function (WOMAC-function), mean (SD)  IG (n=21): 52.2 (13.8)  CG (n=21): 49.3 (18.3) |
|  | Mood:  Anxiety disorder, number (%)  IG (n=21): 8 (38.1%)  CG (n=21): 6 (28.6%)  Depressive disorder, number (%)  IG (n=21): 10 (47.6%)  CG (n=21): 10 (47.6%) |
| **Study design (RCT type)** | Parallel-group RCT |
| **Intervention description (name, duration)** | Name: Positive Psychological Intervention |
|  | Duration: 6 weeks |
| **Control arm description** | Neutral Programme - consists of affectively neutral activities |
| **Data collection time points** | Baseline, 1 month, 3 months, 6 months |
| **Outcome measures for treatment effects** | WOMAC – Total score, stiffness, pain, physical function subscales  10-item Positive and Negative Affect Schedule (PANAS)  Satisfaction With Life Scale |
| VA=Veterans Affairs; OA=osteoarthritis; IG=intervention group; CG=control group SD=standard deviation; ADL=activities of daily living; WOMAC= Western Ontario MacMaster Osteoarthritis Index; RCT=randomised controlled trial | |

**Hurley (2007), UK [3] [3, 42, 43]**

| **Eligibility criteria** | Inclusion criteria:  • Age 50 years or older  • Had consulted a primary care physician for mild, moderate, or severe knee pain of >6 months’ duration. Many participants’ condition had been labeled OA based on their clinical presentation and history without attempting to identify the cause of pain using investigations not routinely available to primary care physicians (e.g., radiographs). |
| --- | --- |
|  | Exclusion criteria:  • Lower limb arthroplasty, physiotherapy for knee pain in the preceding 12 months, intraarticular injections in the preceding 6 months;  • Unstable medical conditions;  • Inability/unwillingness to exercise, wheelchair dependence, and inability to understand English;  • Participants were not excluded if they used assistive walking devices; had stable comorbidities common in this age group (e.g., type II diabetes, cardiovascular or respiratory disorders); or had back, lower, or upper limb pain. |
| **Sample size (assigned to intervention and control arms)** | Individual-rehab IG: n=146  Group-rehab IG: n=132  CG: n=140 |
| **Participant demographic characteristics (Age, Gender, Ethnicity)** | Age, mean (SD), years:  Individual-rehab IG (n=146): 66  Group-rehab IG (n=132): 68  CG (n=140): 67  Overall range: 50-91 |
|  | Gender (Female), Number (%):  Individual-rehab IG (n=146): 104 (71%)  Group-rehab IG (n=132): 94 (71%)  CG (n=140): 96 (68%) |
|  | Ethnicity: Not reported |
| **Participant health status (Pain duration, Pain intensity, Comorbidities, ADL restriction due to pain, mood)** | Pain duration:  Duration of symptoms (years), median (IQR)  Individual-rehab IG (n=146): 7 (3-15)  Group-rehab IG (n=132): 5 (2.5-11)  CG (n=140): 6 (3-15) |
|  | Pain intensity:  Pain (WOMAC-pain), mean (95% CI)  All participants (n=418): 7.6 (95% CI 7.2, 8.0) |
|  | Comorbidities: Not reported |
|  | ADL (or physical function):  Physical function (WOMAC-function), mean (95% CI)  All participants (n=418): 27.2 (95% CI 25.7, 28.6)  Aggregate functional performance time of four common activities of daily living (AFPT) (seconds), mean (95%CI)  All participants (n=418): 63.3 (95% CI 59.8, 66.7) |
|  | Mood:  HADS anxiety, mean (95% CI)  All participants (n=418): 6.53 (95% CI 6.11, 6.95)  HADS depression, mean (95% CI)  All participants (n=418): 4.86 (95%CI 4.53, 5.20) |
| **Study design (RCT type)** | Parallel-group RCT |
| **Intervention description (name, duration)** | Name: Enabling Self-management and Coping with Arthritic knee Pain through Exercise (ESCAPE-knee pain) |
|  | Duration: 6 weeks |
| **Control arm description** | Usual primary care (whatever intervention a participant’s primary care physician considered to be required and appropriate) |
| **Data collection time points** | Baseline, 6 weeks, 6 months (6 months after intervention ended) |
| **Outcome measures for treatment effects** | WOMAC – Total score, pain, physical function subscales  HADS – anxiety, depression subscales  EQ-5D  QALY from EQ-5D  Aggregate functional performance time (AFPT]  Exercise health beliefs and self-efﬁcacy questionnaire (ExBeliefs)  Intervention costs  Cost-utility analysis |
| OA= osteoarthritis; IG=intervention group; CG=control group SD=standard deviation; ADL=activities of daily living; WOMAC= Western Ontario MacMaster Osteoarthritis Index; HADS=hospital anxiety and depression scale; RCT=randomised controlled trial; EQ-5D=EuroQol 5 Dimension; QALY= quality of life | |

**Janevic (2022), USA [9]**

| **Eligibility criteria** | Inclusion criteria:  • Aged 60 and older;  • Ambulatory with or without an assistive device, with musculoskeletal pain of at least 3 months’ duration, with at least one day in the last 30 days when pain made it difficult to do usual activities;  • Had to have a smartphone or other means of watching online videos. |
| --- | --- |
|  | Exclusion criteria:  • Serious acute illness or hospitalization in the last month or planned major surgery in the next 3 months.  • Participants were asked if they had significant memory difficulties that interfered with daily activities. If they answered yes, they were read a description of the program and asked if their memory challenges would get in the way of being able to do the program; participants responding no were eligible. |
| **Sample size (assigned to intervention and control arms)** | IG: n=25  CG: n=26 |
| **Participant demographic characteristics (Age, Gender, Ethnicity)** | Age, mean (SD), years:  IG (n=22): 72.0 (6.9)  CG (n=24): 72.3 (7.6)  Overall range: 60-90 |
|  | Gender (Female), Number (%):  IG (n=22): 20 (91%)  CG (n=24): 21 (88%) |
|  | Ethnicity:  African American, number (%)  IG (n=22): 20 (91%)  CG (n=24): 23 (96%)  The remaining participants were identified as multiracial. |
| **Participant health status (Pain duration, Pain intensity, Comorbidities, ADL restriction due to pain, mood)** | Pain duration: Not reported |
|  | Pain intensity:  Pain intensity (11- point numeric rating scale), mean (SD)  IG (n=22): 6.1 (1.8)  CG (n=24): 6.3 (1.5) |
|  | Comorbidities:  Chronic conditions, number (%)  IG (n=22):  Arthritis: 20 (91%)  Low back pain: 17 (77%)  Hypertension: 20 (91%)  Diabetes: 5 (23%)  Heartburn/acid reflux: 22 (59%)  Depression: 10 (46%)  CG (n=24):  Arthritis: 22 (92%)  Low back pain: 19 (79%)  Hypertension: 19 (79%)  Diabetes: 8 (33%)  Heartburn/acid reflux: 15 (63%)  Depression: 7 (29%)  Count of chronic conditions, mean (SD)  IG (n=22): 6.2 (2.3)  CG (n=24): 5.6 (2.0) |
|  | ADL (or physical function):  Pain Interference (PROMIS), mean (SD)  IG (n=22): 59.4 (6.7)  CG (n=24): 58.9 (8.2)  Physical Functioning (PROMIS), mean (SD)  IG (n=22): 41.5 (7.4)  CG (n=24): 40.8 (6.7) |
|  | Mood:  Depression (comorbidity), number (%)  IG (n=22): 10 (46%)  CG (n=24): 7 (29%) |
| **Study design (RCT type)** | Parallel-group RCT |
| **Intervention description (name, duration)** | Name: Positive STEPS (Seniors using Technology to Engage in Pain Self-management) |
|  | Duration: 7 weeks |
| **Control arm description** | Waitlist |
| **Data collection time points** | Baseline, 8 weeks |
| **Outcome measures for treatment effects** | PROMIS-43 Adult Profile subscale  PROMIS-29 Adult Profile subscale - social participation, physical function scales  11-point numeric rating scale  Participant global impression of change (in function and pain)  Connor-Davidson Resilience Scale  Pain self-efficacy  Amount of medication use |
| IG=intervention group; CG=control group SD=standard deviation; ADL=activities of daily living; PROMIS= Patient-Reported Outcomes Measurement Information System; RCT=randomised controlled trial | |

**Jessep (2009), UK [4]**

| **Eligibility criteria** | Inclusion criteria:  • Over 50 years of age;  • Had consulted a primary care physician for mild, moderate or severe non-speciﬁc knee pain lasting for more than 6 months with no identiﬁable recent cause;  • Would be diagnosed as having clinical osteoarthritis based on their clinical presentation and history. |
| --- | --- |
|  | Exclusion criteria:  • Over 50 years of age;  • Had consulted a primary care physician for mild, moderate or severe non-speciﬁc knee pain lasting for more than 6 months with no identiﬁable recent cause;  • Would be diagnosed as having clinical osteoarthritis based on their clinical presentation and history. |
| **Sample size (assigned to intervention and control arms)** | IG: n=29  CG: n=35 |
| **Participant demographic characteristics (Age, Gender, Ethnicity)** | Age, mean (SD), years:  IG (n=29): 66  CG (n=35): 67  Overall range: 51-81 |
|  | Gender (Female), Number (%):  IG (n=29): 22 (76%)  CG (n=35): 22 (63%) |
|  | Ethnicity: Not reported |
| **Participant health status (Pain duration, Pain intensity, Comorbidities, ADL restriction due to pain, mood)** | Pain duration:  Duration of knee pain (years), mean (range):  IG (n=29): 13 (1 to 30)  CG (n=35): 12 (0.5 to 55) |
|  | Pain intensity:  Pain (WOMAC-pain), mean (SD)  IG (n=29): 5.6 (3.4)  CG (n=35): 5.7 (3.2) |
|  | Comorbidities: Not reported |
|  | ADL (or physical function):  Physical function (WOMAC-function), mean (SD)  IG (n=29): 16.1 (11.8)  CG (n=35): 15.9 (10.4)  0-68, lower is better  Aggregate functional performance time of four common activities of daily living (AFPT) (seconds), mean (SD)  IG (n=29): 41.8 (11.9)  CG (n=35): 43.5 (12.8) |
|  | Mood:  HADS-anxiety, mean (SD):  IG (n=29): 4.2 (2.9)  CG (n=35): 3.6 (2.4)  HADS-depression, mean (SD):  CG (n=35): 2.7 (1.7)  IG (n=29): 2.7 (1.7) |
| **Study design (RCT type)** | Parallel-group RCT |
| **Intervention description (name, duration)** | Name: Enabling Self-management and Coping with Arthritic knee Pain through Exercise (ESCAPE-knee pain) - Adopted from ESCAPE-knee pain, not the original: number of sessions reduced and review session added for 4 months after intervention ended. |
|  | Duration: 5 weeks |
| **Control arm description** | Standard intervention available:  Outpatient physiotherapy followed the usual clinical practice of Sevenoaks Hospital Physiotherapy Outpatient Department |
| **Data collection time points** | Baseline, 5 weeks, 12 months |
| **Outcome measures for treatment effects** | WOMAC – pain, physical function subscales  EQ-5D  QALY from EQ5D  Aggregate functional performance time (AFPT)  Exercise health beliefs and self-efﬁcacy questionnaire (ExBeliefs)  Intervention costs |
| IG=intervention group; CG=control group SD=standard deviation; ADL=activities of daily living; WOMAC= Western Ontario MacMaster Osteoarthritis Index; HADS=hospital anxiety and depression scale; RCT=randomised controlled trial; EQ-5D=EuroQol 5 Dimensions; QALY= quality of life | |

**Kwok (2016), Hong Kong[26]**

| **Eligibility criteria** | Inclusion criteria:  • Aged 60 or above, who had been recruited at a mobile health centre in Hong Kong;  • Had chronic knee pain living in the community, experienced persistent knee pain for at least 3 months were screened for eligibility to participate in the study;  • Self-reported musculoskeletal pain, and no diagnostic investigations were conducted to investigate causes of their pain;  • Had a VAS score of 40 or above;  • All were able to communicate in Cantonese and therefore had no difficulty participating in activities during the program. |
| --- | --- |
|  | Exclusion criteria:  • Diagnosed with osteoporosis, rheumatoid arthritis, gout, a mental disorder (e.g., hallucination, dementia, or depression), were suffering from a complicated spinal problem (e.g., nerve root compression or a perforated vertebral disc), or had active cancer;  • Those who had problems following instructions (e.g., due to a hearing impairment);  • Had undergone surgery or been hospitalized in the previous 6 months;  • To avoid mixing the effect of multiple treatments, older adults who had participated in other intensive health promotion programs or received other treatment modalities within the previous 6 months were not included in the study. |
| **Sample size (assigned to intervention and control arms)** | IG: n=19  CG: n=27 |
| **Participant demographic characteristics (Age, Gender, Ethnicity)** | Age, mean (SD), years:  IG (n=19): 70  CG(n=27): 73 |
|  | Gender (Female), Number (%):Not reported |
|  | Ethnicity: Presumably all Hong Kong Chinese |
| **Participant health status (Pain duration, Pain intensity, Comorbidities, ADL restriction due to pain, mood)** | Pain duration: Not reported |
|  | Pain intensity: Not reported |
|  | Comorbidities: Not reported |
|  | ADL (or physical function): Not reported |
|  | Mood:  SF-36 Mental Health subscale, mean (SD)  IG (n=19): 77.37 (18.06)  CG (n=27): 75.00 (22.62)  SF-36 Mental Component Summary, mean (SD)  IG (n=19): 53.19 (9.39)  CG (n=27): 53.19 (9.39) |
| **Study design (RCT type)** | Parallel-group RCT |
| **Intervention description (name, duration)** | Name: Self-management programme on pain |
|  | Duration: 6 weeks |
| **Control arm description** | Waitlist |
| **Data collection time points** | Baseline, 1 month, 6 weeks (end of intervention), 10 weeks (1 months after intervention ended) |
| **Outcome measures for treatment effects** | VAS  SF-36 - General Health, vitality, physical Functioning subscales, Physical component summary  6-Minute Walk Test (6MWT)  Chair Stand Test (CST)  Chinese version of the Pain Self-efficacy Questionnaire (PSEQ-HK) |
| VAS=visual analogue scale; IG=intervention group; CG=control group SD=standard deviation; ADL=activities of daily living; SF-36=36-Item Short Form Health Survey; RCT=randomised controlled trial | |

**Li (2020), Hong Kong[10, 44]**

| **Eligibility criteria** | Inclusion criteria:  • ≥60 years of age;  • Non-cancer pain duration ≥3 months;  • Pain score ≥2 assessed on a 0 to 10 numeric rating scale (NRS);  • Mainly cared for by informal caregivers;  • Able to understand Cantonese;  • Have suﬃcient behavioural abilities to take part in a light exercise and stretching program;  • Has an informal caregiver who owns a mobile phone and who can access the internet;  Able to join in whole program with their informal caregivers. |
| --- | --- |
|  | Exclusion criteria:  • Had undergone medical or surgical treatment in the past two months;  • Had a history of serious organic disease, a malignant tumour, loss of consciousness, a mental disorder, a drug addiction problem, or were on scheduled pain medications. |
| **Sample size (assigned to intervention and control arms)** | IG: n=32  CG: n=32 |
| **Participant demographic characteristics (Age, Gender, Ethnicity)** | Age, mean (SD), years:  IG (n=32): 70.7 (15.8)  CG (n=32): 72.6 (13.7)  Overall range: 61-92 |
|  | Gender (Female), Number (%):  IG (n=32): 23 (72%)  CG (n=32): 24 (75%) |
|  | Ethnicity:  Presumably 100% Chinese |
| **Participant health status (Pain duration, Pain intensity, Comorbidities, ADL restriction due to pain, mood)** | Pain duration: Not reported |
|  | Pain intensity:  Pain score (BPI-C), mean (SD):  IG (n=32): 4.25 (1.05)  CG (n=32): 4.43 (1.09) |
|  | Comorbidities:  Chronic diseases, number (%)  IG (n=32):  Heart disease: 9 (28%)  Diabetes: 13 (41%)  Hypertension: 16 (50%)  Tracheal disease: 5 (17%)  Cataract: 6 (19%)  Stroke: 3 (9%)  Arthritis: 8 (25%)  Gout: 3 (9%)  Other chronic disease: 3 (9%)  CG (n=32):  Heart disease: 7(22%)  Diabetes: 12 (38%)  Hypertension: 14 (44%)  Tracheal disease: 7 (22%)  Cataract: 3 (9%)  Stroke: 4 (13%)  Arthritis: 9 (28%)  Gout: 5 (17%)  Other chronic disease: 4 (13%) |
|  | ADL (or physical function):  Pain interference with activities of life in the previous 24 hours (BPI-C), mean (SD)  IG (n=32): 2.85 (1.52)  CG (n=32): 2.89 (1.70) |
|  | Mood:  Depression, anxiety, and stress status (DASS-21), mean (SD)  DAS-Depression:  IG (n=32): 8.75 (6.79)  CG (n=32): 8.63 (6.47)  DAS-Anxiety:  IG (n=32): 10.26 (6.32)  CG (n=32): 10.71 (6.94)  DAS-Stress  IG (n=32): 11.50 (7.55)  CG (n=32): 11.00 (6.18) |
| **Study design (RCT type)** | Parallel-group RCT |
| **Intervention description (name, duration)** | Name: Dyadic pain management program (DPMP) |
|  | Duration: 8 weeks |
| **Control arm description** | Provided with a page of simple pain-related information + waitlist |
| **Data collection time points** | Baseline, 8 weeks |
| **Outcome measures for treatment effects** | BPI-C  Chinese Version of DASS-21  World Health Organization Quality of Life-BREF (WHOQOL-BREF)  Pain Self-Eﬃcacy Questionnaire-Chinese Version  Exercise time per week |
| IG=intervention group; CG=control group SD=standard deviation; ADL=activities of daily living; BPI-C= Brief Pain Inventory—Chinese Version; DASS-21=Depression Anxiety Stress Scales 21-item; RCT=randomised controlled trial | |

**Morone (2008), USA [24]**

| **Eligibility criteria** | Inclusion criteria:  • 65 years of age or older;  • Had intact cognition MMSE ≥23);  • Had CLBP, deﬁned as moderate pain occurring daily or almost every day for at least the previous three months;  • Spoke English. |
| --- | --- |
|  | Exclusion criteria:  • 65 years of age or older;  • Had intact cognition MMSE ≥23);  • Had CLBP, deﬁned as moderate pain occurring daily or almost every day for at least the previous three months;  • Spoke English. |
| **Sample size (assigned to intervention and control arms)** | IG: n=19  CG: n=18 |
| **Participant demographic characteristics (Age, Gender, Ethnicity)** | Age, mean (SD), years:  IG (n=19): 74.1 (6.1)  CG (n=18): 75.6 (5.0)  Overall range: 65-84 |
|  | Gender (Female), Number (%):  IG (n=19): 10 (53%)  CG (n=18): 11 (61%) |
|  | Ethnicity:  IG (n=19):  White: 17  African American: 1  Asian: 1  CG (n=18):  White: 16  African American: 1  Asian: 1 |
| **Participant health status (Pain duration, Pain intensity, Comorbidities, ADL restriction due to pain, mood)** | Pain duration: Not reported |
|  | Pain intensity: Pain intensity (Short form McGill Pain Questionnaire), mean (SD)  IG (n=19): 15.5 (10.0)  CG (n=18): 15.2 (7.0)  SF-36 Pain Scale, mean (SD):  Intervention (n=19): 35.5 (6.0)  Control (n=18): 35.7 (7.2) |
|  | Comorbidities:  Low back pain cause, number (%)  IG (n=19):  Osteoarthritis: 17 (89%)  Injury: 0 (0%)  Fibromyalgia: 0 (0%)  Not sure: 2 (11%)  CG (n=18):  Osteoarthritis: 16 (88%)  Injury: 1 (6%)  Fibromyalgia: 1 (6%)  Not sure: 0 (0%) |
|  | ADL (or physical function):  Physical disability (RMDQ) (0-24), mean (SD)  IG (n=19): 11.5 (3.7)  CG (n=18): 11.8 (4.6) |
|  | Mood:  SF-36 Mental Health Composite, mean (SD)  IG(n=19): 41.7 (11.3)  CG (n=18): 40.8 (13.7) |
| **Study design (RCT type)** | Parallel-group RCT |
| **Intervention description (name, duration)** | Name: Mindfulness meditation programme |
|  | Duration: 8 weeks |
| **Control arm description** | Waitlist |
| **Data collection time points** | Baseline, 8 weeks, 3 months |
| **Outcome measures for treatment effects** | RMDQ  McGill Pain Questionnaire Short Form  Chronic Pain Acceptance Questionnaire  SF-36 - Mental Health Composite, global Health Composite, physical Function Scale, Physical Health Composite, pain scale |
| MMSE=Mini-Mental Status Exam; CLBP= chronic low back pain; IG=intervention group; CG=control group SD=standard deviation; ADL=activities of daily living; SF-36=36-Item Short Form Health Survey; RMDQ=Roland Morris Disability Questionnaire; RCT=randomised controlled trial | |

**Morone (2009), USA [21]**

| **Eligibility criteria** | Inclusion criteria:  • CLBP of at least 3 months duration and of at least moderate intensity according to a vertical verbal descriptor scale (pain thermometer);  • Age 65 years or older;  • Intact cognition (Mini-Mental Status Exam ≥24) |
| --- | --- |
|  | Exclusion criteria:  • Non-English speaking;  • Previous participation in a mindfulness meditation program;  • Serious hearing or vision impairment that would preclude responding to questionnaires or participating in the meditation program;  • Medical instability from heart or lung disease;  • Multiple recent falls or inability to stand independently;  • Pain caused by an acute injury in the previous 3 months, and underlying red ﬂags of serious underlying illness such as recent unexplained weight loss, fever, or sudden worsening of back pain. |
| **Sample size (assigned to intervention and control arms)** | IG: n=20  CG: n=20 |
| **Participant demographic characteristics (Age, Gender, Ethnicity)** | Age, mean (SD), years:  IG (n=16): 78 (7.1)  CG (n=19): 73 (6.2)  Dropouts (IG (n=4) + CG (n=1)): 84 |
|  | Gender (Female), Number (%):  IG (n=16): n=11 (69%)  CG (n=19): n=11 (57%) |
|  | Ethnicity:  IG (n=16):  White: 15 (94%)  African American: 1 (6%)  Asian: 0 (0%)  CG (n=19):  White: 15 (79%)  African American: 3 (16%)  Asian: 1 (5%) |
| **Participant health status (Pain duration, Pain intensity, Comorbidities, ADL restriction due to pain, mood)** | Pain duration:  Pain duration^a^, mean (SD):  IG (n=12): 9.4 (18.1)  CG (n=14): 11.1 (13.7) |
|  | Pain intensity: Not reported |
|  | Comorbidities:  Low back pain cause(s), number (%)  IG (n=16):  Osteoarthritis: 10 (63%)  Disc herniation: 1 (6%)  Unknown: 4 (25%)  Failed back surgery: 1 (6%)  Scoliosis: 0 (0%)  Spinal stenosis: 5 (31%)  Spondylolisthesis: 2 (13%)  Osteoporosis: 0 (0%)  CG (n=19):  Osteoarthritis: 9 (47%)  Disc herniation: 1 (5%)  Unknown: 6 (32%)  Failed back surgery: 1 (5%)  Scoliosis: 3 (16%)  Spinal stenosis: 2 (11%)  Spondylolisthesis: 1 (5%)  Osteoporosis: 1 (5%) |
|  | ADL (or physical function):  Physical disability (RMDQ) (0-24), mean (SD), reported in graph only  IG (n=16): ~9  CG (n=19): ~11.5 |
|  | Mood: Not reported |
| **Study design (RCT type)** | Parallel-group RCT |
| **Intervention description (name, duration)** | Name: Eastern methods of mindfulness meditation |
|  | Duration: 8 weeks |
| **Control arm description** | Health education programme in group sessions |
| **Data collection time points** | Baseline, 8 weeks, 4 months |
| **Outcome measures for treatment effects** | Roland Morris disability questionnaire (RMDQ)  McGill Pain Questionnaire Short Form  SF-36 - Pain Scale  Participant global impression of change (in function and pain)  Role Limitations Due to Emotional Problems scale  Chronic Pain Self-Efﬁcacy Scale  Mindful Attention Awareness Scale (MAAS) |
| ^a^= Unit of count is presumed to be "years"; ~=approximately  CLBP= chronic low back pain; IG=intervention group; CG=control group SD=standard deviation; ADL=activities of daily living; RCT=randomised controlled trial; SF-36=36-Item Short Form Health Survey | |

**Morone (2016), USA [24] [45]**

| **Eligibility criteria** | Inclusion criteria:  • 65 years or older;  • Spoke English;  • Had intact cognition (Mini-Mental State Examination score, ≥24), functional limitations owing to their chronic LBP (defined as a score of≥11 on the RMDQ, range 0-24, with higher scores indicating increased limitations), and had self-reported moderate chronic pain levels on a verbal descriptor scale (Pain Thermometer; measured on a visual scale as pain as bad as it could be, extreme, severe, moderate, mild, or no pain) occurring daily or almost every day for at least the previous 3 months. |
| --- | --- |
|  | Exclusion criteria:  • Had participated in a previous mindfulness meditation program;  • Had serious underlying illness (such as malignant neoplasms, infection, unexplained fever, weight loss, or recent trauma) causing their pain,  • Were non-ambulatory, had severe impaired mobility  • Had visual or hearing impairment that interfered with assessments, had pain in other parts of the body more severe than their chronic LBP or acute back pain, had an acute or a terminal illness, or had moderate to severe depressive symptoms (GDS score, ≥21; range, 0-30). |
| **Sample size (assigned to intervention and control arms)** | IG: n=140  CG: n=142 |
| **Participant demographic characteristics (Age, Gender, Ethnicity)** | Age, mean (SD), years:  IG (n=140): 75 (7.2)  CG (n=142): 74 (6.0) |
|  | Gender (Female), Number (%):  IG (n=140): 93 (66.4%)  CG (n=142): 94 (66.2%) |
|  | Ethnicity:  Race, number (%):  IG (n=140):  White: 98 (70.0%)  Black: 42 (30.0%)  Other: 0 (0%)  CG (n=142):  White: 101 (71.1%)  Black: 38 (26.8%)  Other: 3 (2.1%) |
| **Participant health status (Pain duration, Pain intensity, Comorbidities, ADL restriction due to pain, mood)** | Pain duration:  Pain duration (months), mean (SD)  IG (n=140): 137 (156.5)  CG(n=142): 138 (160.3) |
|  | Pain intensity: Not reported |
|  | Comorbidities: Cumulative Illness Rating Scale score, mean (SD):  IG (n=140): 3.4 (2.1)  CG (n=142): 3.2 (1.8) |
|  | ADL (or physical function):  Physical disability (RMDQ) (0-24), mean (SD)  IG (n=140): 15.6 (3.0)  CG (n=142): 15.4 (3.0) |
|  | Mood:  Depression (GDS score), mean (SD)  IG (n=140): 5.7 (4.3)  CG (n=142): 6.0 (4.3) |
| **Study design (RCT type)** | Parallel-group RCT |
| **Intervention description (name, duration)** | Name: Mindfulness-Based Stress Reduction (MBSR) programme |
|  | Duration: 8 weeks + 6 monthly booster sessions |
| **Control arm description** | 10 Keys to Healthy Aging (health education programme) + monthly booster for 6 months |
| **Data collection time points** | Baseline, 8 weeks, 6 months |
| **Outcome measures for treatment effects** | RMDQ  Numeric Pain Rating Scale  Catastrophizing Scale of the Coping Strategies Questionnaire  GDS  SF-36 - Global Health Composite, Physical Health Composite  Chronic Pain Self-Efficacy Scale  Mindful Attention Awareness Scale |
| LBP=low back pain; IG=intervention group; RMDQ=Roland Morris Disability Questionnaire; GDS=Geriatric Depression Scale CG=control group SD=standard deviation; ADL=activities of daily living; RCT=randomised controlled trial; SF-36=36-Item Short Form Health Survey | |

**Nicholas (2013), Australia [23] [46]**

| **Eligibility criteria** | Inclusion criteria:  • Aged 65 years or over;  • History of persisting, noncancer pain for more than 6 months;  • Still seeking help for their pain and its effects on lifestyle or mood;  • Able to attend the 2-hour sessions at the pain centre twice weekly for 4 weeks;  • Ability to read and speak adequate English to complete written questionnaires and participate in group discussions conducted in English;  • Score of 22 or greater in the Rowland Universal Dementia Assessment Scale (i.e., evidence of normal range short-term memory functioning);  • Have clearance by their doctors for participation in a light exercise and stretch program;  • Agree to accept randomisation to one of the intervention groups after a full explanation of the project. |
| --- | --- |
|  | Exclusion criteria:  • Presence of an active major mental disorder (e.g., psychoses, dementia, major depression with active suicidal ideation);  • Further medical/surgical treatments or investigations for pain condition planned;  • Evidence of a primary drug addiction problem. |
| **Sample size (assigned to intervention and control arms)** | IG: n=49  Exercise-attention CG: n=53  Waitlist CG: n=39 |
| **Participant demographic characteristics (Age, Gender, Ethnicity)** | Age, mean (SD), years:  IG (n=49): 74.59 (5.98)  Exercise-attention CG (n=53): 72.40 (5.5)  Waitlist CG (n=39): 74.95 (6.6)  Overall range: 65-87 |
|  | Gender (Female), Number (%):  IG (n=49): 32 (65%)  Exercise-attention CG (n=53): 37 (70%)  Waitlist CG (n=39): 20 (52%) |
|  | Ethnicity: Not reported |
| **Participant health status (Pain duration, Pain intensity, Comorbidities, ADL restriction due to pain, mood)** | Pain duration:  Pain duration (months), mean (SD)  IG (n=49): 207 (219)  Exercise-attention CG (n=53): 179 (216)  Waitlist CG (n=39): 135 (177)  Overall range: 7-780 |
|  | Pain intensity:  Usual pain intensity (0-10), mean (SD)  IG (n=49): 5.32 (2.05)  Exercise-attention CG (n=53): 5.48 (2.07)  Waitlist CG (n=39): 5.67 (2.26) |
|  | Comorbidities:  Overall: around 70% reported one or more ongoing diseases or health conditions (including joint diseases, cardiovascular conditions, visual impairments, gastrointestinal conditions, and endocrine, skin, and bladder disorders).  Mean (SD) number of comorbidities: 3.05 (2.16) |
|  | ADL (or physical function):  Physical disability (mRMDQ) (0-24), mean (SD)  IG (n=49): 12.93 (5.37)  Exercise-attention CG (n=53): 12.24 (5.08)  Waitlist CG (n=39): 12.67 (4.84) |
|  | Mood:  Depression (DASS-42), mean (SD)  IG (n=49): 10.8 (11.06)  Exercise-attention CG (n=53): 9.34 (8.04)  Waitlist CG (n=39): 12.0 (10.4) |
| **Study design (RCT type)** | Parallel-group RCT |
| **Intervention description (name, duration)** | Name: Pain self-management (PSM) program |
|  | Duration: 4 weeks |
| **Control arm description** | 2 control arms:  Exercise-Attention Control (EAC) group  Waitlist control |
| **Data collection time points** | Baseline, 1 month |
| **Outcome measures for treatment effects** | mRMDQ  Usual pain intensity  Pain Response Self-statements Scale (PRSS) - Catastrophising scale  Usual pain-related distress  DASS-21  Functional reach test  6-min walk  Pain self-efficacy questionnaire (PSEQ)  Fear avoidance (TAMPA Scale for Kinesiophobia) |
| IG=intervention group; CG=control group SD=standard deviation; ADL=activities of daily living; mRMDQ=modified Roland Morris Disability Questionnaire; DASS= Depression Anxiety Stress Scales; RCT=randomised controlled trial | |

**Rini (2015), USA [5] [47]**

| **Eligibility criteria** | Inclusion criteria:  • Adults (18 years old) with knee or hip OA, confirmed radiographically (KL grade 2, with pain in the affected joint), with American College of Rheumatology clinical criteria, or by their physician.  • Had to speak English and report having frequent OA pain (defined as pain on most days of the month for each of the prior three months). |
| --- | --- |
|  | Exclusion criteria:  • Had significant cognitive impairment (3 or more incorrect responses on a validated six-item screener);  • Less than 7th grade reading proficiency (three-item health literacy screener);  • Medical comorbidities that interfered with their ability to complete the intervention or that indicated they had a pain-related condition in addition to OA (uncorrectable moderate or severe hearing or vision deficits, Parkinson’s disease, cancer pain, rheumatoid arthritis, fibromyalgia, diabetic neuropathy, arthroscopic surgery or total knee- or hip-replacement surgery in the past six months, fractures in the past six months, history of falls in the past three months, or vertigo in the past month). |
| **Sample size (assigned to intervention and control arms)** | IG: n=58  CG: n=55 |
| **Participant demographic characteristics (Age, Gender, Ethnicity)** | Age, mean (SD), years:  IG (n=58): 68.52 (7.65)  CG (n=55): 66.67 (11.02)  Overall range 38-90 |
|  | Gender (Female), Number (%):  IG (n=58): 46 (79%)  CG (n=55): 45 (82%) |
|  | Ethnicity:  Ethnicity, number (%)  IG (n=58):  Not Hispanic/Latino: 51 (88%)  Hispanic/Latino: 7 (12%)  CG (n=55):  Not Hispanic/Latino: 50 (91%)  Hispanic/Latino: 5 (9%)  Race, number (%)  IG (n=58):  White: 38 (66%)  African American/Black: 20 (35%)  Other race: 0 (0%)  CG (n=55):  White: 41 (75%)  African American/Black: 13 (24%)  Other race: 1 (2%) |
| **Participant health status (Pain duration, Pain intensity, Comorbidities, ADL restriction due to pain, mood)** | Pain duration: Not reported |
|  | Pain intensity:  Pain (AIMS2 - arthritis pain subscale), mean (SD)  IG (n=58): 4.82 (1.73)  CG (n=55): 5.12 (1.81) |
|  | Comorbidities:  Presence of any of the 14 medical conditions (e.g., diabetes, cancer, vision problems, hearing problems, and arthritis of the hand or wrist)  Medical comorbidities (number), mean (SD)  IG (n=58): 1.34 (1.02)  CG (n=55): 1.31 (1.05) |
|  | ADL (or physical function):  Pain-related functioning (AIMS2), mean (SD)  IG (n=58): 1.70 (1.30)  CG (n=55): 1.89 (1.03) |
|  | Mood:  Pain Anxiety Symptoms Scale, mean (SD):  IG (n=58): 26.79 (19.81)  CG (n=55): 29.97 (18.21) |
| **Study design (RCT type)** | Parallel-group RCT |
| **Intervention description (name, duration)** | Name: Internet-based pain coping skills training (PainCOACH) |
|  | Duration: 8 weeks |
| **Control arm description** | Assessment only + standard care for OA |
| **Data collection time points** | Baseline (prior to randomization), midway through the intervention period (midpoint assessment), and after completion of the intervention (post-intervention assessment, approximately 9 to 11 weeks after randomization). |
| **Outcome measures for treatment effects** | AIMS2 - pain-related interference, pain, self-efficacy subscales  Pain Anxiety Symptoms Scale  Positive and Negative Affect Schedule (PANAS) |
| OA=osteoarthritis; IG=intervention group; CG=control group SD=standard deviation; ADL=activities of daily living; AIMS2=Arthritis Impact Measurement Scale 2; RCT=randomised controlled trial | |

**Tse (2013), Hong Kong [6]**

| **Eligibility criteria** | Inclusion criteria:  • Aged 65 and above;  • Suffering from chronic musculoskeletal pain for more than three months [meeting the deﬁnition of the International Association for the Study of Pain (1986)];  • Ability to communicate in Cantonese;  • Being oriented as to time and place. |
| --- | --- |
|  | Exclusion criteria: Not reported separately |
| **Sample size (assigned to intervention and control arms)** | IG: n=31  CG: n=25 |
| **Participant demographic characteristics (Age, Gender, Ethnicity)** | Age, mean (SD), years:  IG (n=31): 75.9 (6.4)  CG (n=25): 77.2 (5.1) |
|  | Gender (Female), Number (%):  IG (n=31): 28 (93.3%)  CG (n=25): 22 (95.7%) |
|  | Ethnicity: Presumably all Hong Kong Chinese |
| **Participant health status (Pain duration, Pain intensity, Comorbidities, ADL restriction due to pain, mood)** | Pain duration: Not reported |
|  | Pain intensity: Not reported |
|  | Comorbidities:  IG (n=31):  Hypertension: 17 (75.6%)  Arthritis: 4 (13.3%)  Heart disease: 3 (10.0%)  Diabetes: 3 (10.0%)  Cataract: 2 (6.7%)  No: 7 (23.3%)  CG (n=25):  Hypertension: 10 (43.5%)  Arthritis: 10 (43.5%)  Heart disease: 5 (21.7%)  Diabetes: 6 (26.1%)  Cataract: 6 (26.1%)  No: 3 (15.6) |
|  | ADL (or physical function):  Elderly Mobility Scale, mean (SD)  IG (n=31): 16.36 (1.86)  CG (n=25): 18.55 (2.58) |
|  | Mood:  Psychological parameters, mean (SD)  IG (n=31):  Anxiety - state: 43.06 (12.94)  Anxiety - trait: 35.39 (7.04)  Depression: 3.17 (7.04)  Happiness: 22.06 (3.11)  SF12-mental: 58.17 (6.64)  CG (n=25):  Anxiety - state: 36.65 (7.24)  Anxiety - trait: 36.22 (6.35)  Depression: 2.11 (2.17)  Happiness: 20.22 (3.12)  SF12-mental: 58.27 (6.55) |
| **Study design (RCT type)** | Parallel-group RCT |
| **Intervention description (name, duration)** | Name: Integrated motivational interviewing and physical exercise programme |
|  | Duration: 8 weeks |
| **Control arm description** | Usual care |
| **Data collection time points** | Baseline, 8 weeks |
| **Outcome measures for treatment effects** | Numerical rating scale (NRS)  State-Trait Anxiety Inventory (STAI)  Geriatric Depression Scale – Short Form  Subjective Happiness Scale  SF-12 – Mental health, physical health components  Elderly Mobility Scale (EMS): gait speed, functional reach and change in position  Pain Self-Efficacy Questionnaire (PSEQ) |
| IG=intervention group; CG=control group SD=standard deviation; ADL=activities of daily living; SF-12=12-Item Short Form Health Survey; RCT=randomised controlled trial | |

**Vitiello (2013), USA [19] [48]**

| **Eligibility criteria** | Inclusion criteria:  • Clinically significant pain and insomnia were eligible for enrolment.  • Significant arthritis pain was defined as Grade II, III, or IV pain on the Graded Chronic Pain Scale.  • Significant insomnia was defined as meeting research diagnostic criteria for insomnia based on self-reported sleep difficulties (trouble falling asleep, difficulty staying asleep, waking up too early, or waking up unrefreshed), 3 or more nights per week during the past month with at least one daytime sleep-related problem. |
| --- | --- |
|  | Exclusion criteria:  • Determined through electronic health records and included diagnosis of rheumatoid arthritis, obstructive sleep apnoea, periodic leg movement disorder, restless leg syndrome, sleep-wake cycle disturbance, rapid eye movement behaviour disorder, dementia or receiving cholinesterase inhibitors, Parkinson’s disease, cancer in the past year, receiving chemotherapy or radiation therapy in the past year, and inpatient treatment for congestive heart failure within the prior 6 months.  • Unable to read a newspaper, difficulty hearing in a group situation, unable to walk across a room without help. |
| **Sample size (assigned to intervention and control arms)** | CBT for pain IG: n=122 (12 clusters)  CBT for pain & insomnia IG: n=122 (13 clusters)  Education only CG: n=123 (14 clusters) |
| **Participant demographic characteristics (Age, Gender, Ethnicity)** | Age, mean (SD), years:  CBT for pain IG (n=122): 73.0 (8.4)  CBT for pain & insomnia IG (n=122): 73.2 (8.1)  Education only CG (n=123): 73.1 (8.0) |
|  | Gender (Female), Number (%):  CBT for pain IG (n=122): 80.3%  CBT for pain & insomnia IG (n=122): 79.5%  Education only CG (n=123): 75.6% |
|  | Ethnicity:  Caucasian, %  CBT for pain IG (n=122): 91.7%  CBT for pain & insomnia IG (n=122): 91.8%  Education only CG (n=123): 90.2% |
| **Participant health status (Pain duration, Pain intensity, Comorbidities, ADL restriction due to pain, mood)** | Pain duration: Not reported |
|  | Pain intensity:  Pain severity (Graded Chronic Pain Scale), mean (SD)  CBT for pain IG (n=122): 4.3 (1.6 )  CBT for pain & insomnia IG (n=122): 4.6 (1.5)  Education only CG (n=123): 4.1 (1.5) |
|  | Comorbidities:  % of participants having chronic illness  CBT for pain IG (n=122): 49.2%  CBT for pain & insomnia IG (n=122): 59.8%  Education only CG (n=123): 49.6%  Over half |
|  | ADL (or physical function):  Pain-related functioning (AIMS2), mean (SD)  CBT for pain IG (n=122): 36.0 (22.5)  CBT for pain & insomnia IG (n=122): 38.6 (22.2)  Education only CG (n=123): 34.6 (23.2) |
|  | Mood:  Geriatric Depression Scale, mean (SD)  CBT for pain IG (n=122): 6.6 (4.5)  CBT for pain & insomnia IG (n=122): 6.5 (5.1)  Education only CG (n=123): 7.0 (5.6)  Overall, 12% participants' scored ≥14, indicated moderate to severe depression. |
| **Study design (RCT type)** | Cluster RCT |
| **Intervention description (name, duration)** | Name: Cognitive-behavioural therapy for pain and insomnia (CBT-PI); a cognitive-behavioural pain coping skills intervention (CBT-P) |
|  | Duration: 6 weeks |
| **Control arm description** | Education only control (EOC) intervention: contained educational content related to pain and sleep management, self-help format only. |
| **Data collection time points** | Baseline, 2 months, 9 months |
| **Outcome measures for treatment effects** | Insomnia Severity Index (ISI)  Graded Chronic Pain Scale (GCPS)  AIMS2, Short Form, Revised - Arthritis symptom subscale |
| CBT=cognitive behavioural therapy; IG=intervention group; CG=control group SD=standard deviation; ADL=activities of daily living; RCT=randomised controlled trial; AIMS2=Arthritis Impact Measurement Scale 2 | |

**Walsh (2020), UK [32] [49]**

| **Eligibility criteria** | Inclusion criteria:  • Over 50 with a clinical or radiographic diagnosis of degenerative hip, knee or lower back pain of at least 6 months duration. |
| --- | --- |
|  | Exclusion criteria:  • Physiotherapy in preceding 6 months; lower limb arthroplasty; unstable medical or psychiatric conditions; and non-English speaking. |
| **Sample size (assigned to intervention and control arms)** | IG: n=170  CG: n=179 |
| **Participant demographic characteristics (Age, Gender, Ethnicity)** | Age, mean (SD), years:  IG (n=170): 66.3 (8.1)  CG (n=179): 66.5 (8.4) |
|  | Gender (Female), Number (%):  IG (n=170): 112 (66%)  CG (n=179): 104 (58%) |
|  | Ethnicity: Not reported |
| **Participant health status (Pain duration, Pain intensity, Comorbidities, ADL restriction due to pain, mood)** | Pain duration: Not reported |
|  | Pain intensity:  Pain intensity (Short form McGill Pain Questionnaire), mean (SD)  IG (n=170): 2.2 (2.0)  CG (n=179): 2.3 (2.1) |
|  | Comorbidities: Not reported |
|  | ADL (or physical function):  Dysfunction Index Short Musculoskeletal Functional Assessment (DI-SMFA, irrespective of site of pain), mean (SD)  IG (n=170): 60.4 (16.1)  CG (n=179): 60.5 (17.2) |
|  | Mood:  HADS Anxiety, mean (SD)  IG (n=170): 5.6 (3.7)  CG (n=179): 5.7 (3.7)  HADS Depression, mean (SD)  IG (n=170): 3.9 (2.7)  CG (n=179): 4.2 (3.1) |
| **Study design (RCT type)** | Parallel-group RCT |
| **Intervention description (name, duration)** | Name: Facilitating Activity and Self-management in Arthritic Pain (FASA) |
|  | Duration: 6 weeks |
| **Control arm description** | Continued with ‘standard’ GP care |
| **Data collection time points** | Baseline, 6 weeks, 6 months |
| **Outcome measures for treatment effects** | Short Form McGill Pain questionnaire  HADS – Anxiety, depression subscales  QALY from EQ-5D  EQ-5D  Dysfunction Index of the Short Musculoskeletal Functional Assessment (DISMFA)  Aggregated Functional Performance Time (AFPT)  Self-efﬁcacy and exercise health beliefs questionnaire (SEHBQ)  Weight  Hip to waist ratio  Cost per QALY |
| IG=intervention group; CG=control group SD=standard deviation; ADL=activities of daily living; HADS=hospital anxiety and depression scale; RCT=randomised controlled trial; QALY= quality of life;  EQ-5D=EuroQol 5 Dimensions | |

**Weiner (2020), USA [15]**

| **Eligibility criteria** | Inclusion criteria:  • CLBP, defined as pain in the lower back of at least moderate severity (assessed with a verbal rating scale), every day or almost every day, for at least three months;  • Lumbar magnetic resonance imaging (MRI) within the past 30 days that is without evidence of infection, malignancy, or acute fracture;  • Able to commit to six months of study participation. |
| --- | --- |
|  | Exclusion criteria:  • No red flags indicative of serious underlying illness requiring urgent care (e.g., fever, change in bowel/bladder function, sudden severe change in pain, unintentional weight loss, new lower extremity weakness);  • No prior lumbar surgery;  • No pain in other body locations that is more severe than CLBP; cognitively intact (based on Mini-Mental State Examination (MMSE) score, as described below); psychotic symptoms; no acute illness; no prohibitive communication impairment (e.g., severe hearing or visual impairment). |
| **Sample size (assigned to intervention and control arms)** | IG: n=25  CG: n=30 |
| **Participant demographic characteristics (Age, Gender, Ethnicity)** | Age, mean (SD), years:  IG (n=25): 71.3 (7.5)  CG (n=30): 67.2 (5.5) |
|  | Gender (Female), Number (%):  IG (n=25): 1 (4.0%)  CG (n=30): 1 (3.3%) |
|  | Ethnicity:  Ethnicity, number (%)  IG (n=25):  Hispanic/Latino: 0 (0%)  Not Hispanic/Latino: 25 (100%)  Unknown/not reported: 0 (0%)  CG (n=30):  Hispanic/Latino: 0 (0%)  Not Hispanic/Latino: 26 (86.7%)  Unknown: 1 (3.3%)  Not reported: 3 (10.0%)  Race, number (%)  IG (n=25):  Black: 8 (32.0%)  White: 17 (68.0%)  Unknown: 0 (0%)  CG (n=30):  Black: 9 (30.0%)  White: 20 (66.7%)  Unknown: 1 (3.3%) |
| **Participant health status (Pain duration, Pain intensity, Comorbidities, ADL restriction due to pain, mood)** | Pain duration:  Pain duration, number (%)  IG (n=25):  3-12 months: 3 (12%)  1-5 years: 6 (24%)  >5 years: 16 (64%)  CG (n=30):  3-12 months: 1 (3.3%)  1-5 years: 4 (13.3%)  >5 years: 25 (83.3%) |
|  | Pain intensity:  Pain severity (bespoke scale), mean (SD)  IG (n=25):  Current pain: 4.5 (2.8)  Average pain prior week: 6.6 (1.7)  Worst pain prior week: 8.8 (1.5)  CG (n=30):  Current pain: 5.3 (2.4)  Average pain prior week: 6.5 (1.4)  Worst pain prior week: 8.5 (1.9) |
|  | Comorbidities:  Comorbidities, number (%)  IG (n=25):  Cardiovascular: 9 (36.0%)  Neurological: 4 (16.0%)  Musculoskeletal: 25 (100%)  Visual/hearing: 16 (64.0%)  Diabetes: 11 (44.0%)  Cancer: 3 (12.0%)  Lung: 11 (44.4%)  CG (n=30):  Cardiovascular: 6 (20.0%)  Neurological: 2 (6.7%)  Musculoskeletal: 30 (100%)  Visual/hearing: 22 (73.3%)  Diabetes: 5 (16.7%)  Cancer: 7 (23.3%)  Lung: 5 (16.7%)  Duke comorbidity index, mean (SD):  IG (n=25): 3.9 (1.4)  CG (n=30): 3.2 (1.5) |
|  | ADL (or physical function):  In the prior week pain interference with, quite a bit or very much (Minimum Data Set, National Institutes of Health Task Force), number (%)  Day-to-day activities:  IG (n=25): 20 (80%)  CG (n=30): 25 (83.3%)  Work around the home:  IG (n=25): 21 (84%)  CG (n=30): 20 (66.7%)  Social activities:  IG (n=25): 14 (56%)  CG (n=30): 17 (56.7%)  Household chores:  IG (n=25): 17 (68%)  CG (n=30): 19 (63.3%) |
|  | Mood:  SF-12 - mental Component Summary, mean (SD)  IG (n=25): 51.7 (11.4)  CG (n=30): 51.3 (10.8) |
| **Study design (RCT type)** | Parallel-group RCT |
| **Intervention description (name, duration)** | Name: Aging Back Clinics |
|  | Duration: 6 months |
| **Control arm description** | Usual care |
| **Data collection time points** | Baseline, 6 months |
| **Outcome measures for treatment effects** | Roland & Morris Disability Questionnaire-Modified (mRMDQ)  Low back pain severity (bespoke)  SF-12 - mental component summary, physical component summary |
| CLBP= chronic low back pain; IG=intervention group; CG=control group SD=standard deviation; ADL=activities of daily living; SF-12=12-Item Short Form Health Survey; RCT=randomised controlled trial | |

**Yarns (2020), USA [33]**

| **Eligibility criteria** | Inclusion criteria:  • Veterans age 50 years or older who had had at least three months of musculoskeletal pain, including the following conditions likely to benefit from psychological interventions based on previous research; low back, neck, leg, or pelvic pain; temporomandibular joint disorders; fibromyalgia; tension headaches; or any combination of these conditions. |
| --- | --- |
|  | Exclusion criteria:  • Confirmed hip or knee osteoarthritis, leg pain greater than back pain (to exclude radiculopathy);  • Electromyography-confirmed “tunnel” syndromes (e.g., carpal or tarsal tunnel syndrome), gout, neuralgias, migraine, and cluster headaches.  • Following non-musculoskeletal conditions were excluded: autoimmune disease that typically generates pain (e.g., rheumatoid arthritis), cancer pain, sickle cell disease, burn pain, infection associated with pain, and cauda equina syndrome.  • Following conditions or circumstances were excluded: severe psychiatric disorder such as schizophrenia or bipolar I disorder not controlled with medications, active suicide or violence risk in the past six months, active severe alcohol or substance use disorder;  • Currently enrolled in another psychological treatment for chronic pain, currently in pain-related litigation or applying for pain-related compensation or compensation increase;  • Unable to fluently read or converse in English;  • Planning to move from the area in the next six months.  • Patients were included regardless of prior psychological or medical treatments for their pain.  • Score of <26/30 on the MMSE at baseline screening, due to concerns about cognitive impairment. |
| **Sample size (assigned to intervention and control arms)** | EAET IG: n=28  CBT IG: n=25 |
| **Participant demographic characteristics (Age, Gender, Ethnicity)** | Age, mean (SD), years:  EAET IG (n=28): 73.7 (9.6)  CBT IG (n=25): 73.3 (9.7) |
|  | Gender (Female), Number (%):  EAET IG (n=28): 2 (7.1%)  CBT IG (n=25): 2 (8.0%) |
|  | Ethnicity:  Race/ethnicity, number (%)  EAET IG (n=28):  White: 14 (50.0%)  African American: 9 (32.1%)  Other: 5 (17.9%) :  CBT IG (n=25):  White: 10 (40.0%)  African American: 12 (48.0%)  Other: 3 (12.0%) |
| **Participant health status (Pain duration, Pain intensity, Comorbidities, ADL restriction due to pain, mood)** | Pain duration: Not reported |
|  | Pain intensity: Not reported |
|  | Comorbidities:  Number of medical comorbidities, mean (SD)  EAET IG (n=28): 5.6 (2.5)  CBT IG (n=25): 6.5 (2.2)  Have a comorbid psychiatric diagnosis (n=53): 86.8% |
|  | ADL (or physical function): Not reported |
|  | Mood:  PROMIS - Depression, mean (SD)  EAET IG (n=28): 22.25 (8.93)  CBT IG (n=25): 22.84 (6.44)  PROMIS - Anxiety, mean (SD):  EAET IG (n=28): 21.57 (6.86)  CBT IG (n=25): 23.28 (5.92) |
| **Study design (RCT type)** | Parallel-group RCT |
| **Intervention description (name, duration)** | Name: EAET; and  CBT |
|  | Duration: 8 weeks |
| **Control arm description** | No control (both arms are eligible experimental interventions in this review) |
| **Data collection time points** | Baseline, 8 weeks, 3 months |
| **Outcome measures for treatment effects** | Brief Pain Inventory (BPI)  PROMIS - Anxiety  PROMIS - Depression  PROMIS - Sleep disturbance  PROMIS - Fatigue  National Institutes of Health Toolbox - Life satisfaction  Satisfaction with Therapy and Therapist Scale–Revised (STTS-R) |
| MMSE= Mini-Mental Status Exam; EAET=Emotional Awareness and Expression Therapy; IG=intervention group; CBT=cognitive behavioural therapy; CG=control group SD=standard deviation; ADL=activities of daily living; PROMIS= Patient-Reported Outcomes Measurement Information System; RCT=randomised controlled trial | |

**Table 3 Excluded studies**

| **Study** | **Reason for exclusion** |
| --- | --- |
| Ackerman, et al.[50] | Intervention's main focus is not chronic pain management |
| Alp, et al.[51] | Intervention's main focus is not chronic pain management |
| Baker, et al.[52] | Intervention's main focus is not chronic pain management |
| Basler, et al.[53] | Intervention's main focus is not chronic pain management |
| Bezalel, et al.[54] | Ineligible intervention (not multicomponent, or ineligible stand-alone psychotherapy) |
| Bilterys, et al.[55] | Participant mean age <65 years |
| Birch, et al.[56] | Intervention's main focus is not chronic pain management |
| Bobek, et al.[57] | Intervention's main focus is not chronic pain management |
| Brown, et al.[58] | Participant mean age <65 years |
| Burns, et al.[59] | Participant mean age <65 years |
| Buszewicz, et al.[60] | Intervention's main focus is not chronic pain management |
| Cederbom, et al.[61] | Intervention's main focus is not chronic pain management |
| Cederbom, et al.[62] | Intervention's main focus is not chronic pain management |
| Coleman, et al.[63] | Intervention's main focus is not chronic pain management |
| Corrêa Dias, et al.[64] | Intervention's main focus is not chronic pain management |
| Coupé, et al.[65] | Intervention's main focus is not chronic pain management |
| Foo, et al.[66] | Participant mean age <65 years |
| Fries, et al.[67] | Ineligible intervention (not multicomponent, or ineligible stand-alone psychotherapy) |
| Hausmann, et al.[68] | Participant mean age <65 years |
| Hay, et al.[69] | Ineligible intervention (not multicomponent, or ineligible stand-alone psychotherapy) |
| Holm, et al.[70] | Participant mean age <65 years |
| Hruschak, et al.[71] | Participant mean age <65 years |
| Hughes, et al.[72] | Intervention's main focus is not chronic pain management |
| Hunt, et al.[73] | Participant mean age <65 years |
| Irvine, et al.[74] | Participant mean age <65 years |
| Jensen, et al.[75] | Participant mean age <65 years |
| Jinnouchi, et al.[76] | Ineligible intervention (not multicomponent, or ineligible stand-alone psychotherapy) |
| Lin, et al.[77] | Intervention's main focus is not chronic pain management |
| Macfarlane, et al.[78] | Intervention's main focus is not chronic pain management |
| McCurry, et al.[79] | Intervention's main focus is not chronic pain management |
| Messier, et al.[80] | Ineligible intervention (not multicomponent, or ineligible stand-alone psychotherapy) |
| Miyamoto, et al.[81] | Participant mean age <65 years |
| Murphy, et al.[82] | Intervention's main focus is not chronic pain management |
| Murphy, et al.[83] | Participant mean age <65 years |
| Nunes[84] | Study not completed (including no published results) |
| Østerås, et al.[85] | Intervention's main focus is not chronic pain management |
| Petrozzi, et al.[86] | Not RCT |
| Pimm, et al.[87] | Participant mean age <65 years |
| Pratscher, et al.[88] | Participant mean age <65 years |
| Schulz, et al.[89] | Ineligible intervention (not multicomponent, or ineligible stand-alone psychotherapy) |
| Skovbo, et al.[90] | Study not completed (including no published results) |
| Suso-Ribera, et al.[91] | Participant mean age <65 years |
| Swerissen, et al.[92] | Intervention's main focus is not chronic pain management |
| Tak, et al.[93] | Intervention's main focus is not chronic pain management |
| Tang, et al.[94] | Participant mean age <65 years |
| Tse, et al.[95] | Participants not living in community |
| Turner, et al.[96] | Participant mean age <65 years |
| Vandermost, et al.[97] | Participant mean age <65 years |
| Vitiello, et al.[98] | Intervention's main focus is not chronic pain management |
| Vitiello, et al.[99] | Intervention's main focus is not chronic pain management |
| Yip, et al.[100] | Intervention's main focus is not chronic pain management |
| Yip, et al.[101] | Participant mean age <65 years |

**Table 4. TIDieR checklist**

|  | **Item 1. Brief name** | **Item 2. Why** | **Item 3. What (materials)** | **Item 4. What (procedures)** | **Item 5. Who provided** | **Item 6. How** | **Item 7. Where** | **Item 8. When and how much** | **Item 9. Tailoring** | **Item 10. Modifications** | **Item 11. How well (planned)** | **Item 12: How well (actual)** |
| --- | --- | --- | --- | --- | --- | --- | --- | --- | --- | --- | --- | --- |
| **Andersson et al. [28]** | ● | ● | ● | ● | ● | ● |  | ● |  |  |  | ● |
| **Baird and Sands [29]** | ● | ● | ● | ● | ● | ● | ● | ● | ● |  |  | ● |
| **Bearne et al. [2]** | ● | ● | ● | ● | ● | ● | ● | ● | ● |  |  | ● |
| **Berman et al. [11]** | ● | ● | ● | ● | ● | ● | ● | ● |  |  |  | ● |
| **Broderick et al. [25]** | ● | ● | ● | ● | ● | ● | ● | ● | ● |  |  | ● |
| **Carmody et al. [30]** | ● | ● | ● | ● | ● | ● | ● | ● | ● |  |  | ● |
| **Cheng et al. [20]** | ● | ● | ● | ● | ● | ● | ● | ● | ● |  | ● | ● |
| **Costantino and Romiti [16]** | ● | ● | ● | ● | ● | ● |  | ● |  |  |  | ● |
| **da Silva et al. [17]** | ● | ● | ● | ● | ● | ● |  | ● |  |  |  | ● |
| **Ersek et al. [12]** | ● | ● | ● | ● | ● | ● | ● | ● | ● |  |  | ● |
| **Ersek et al. [13]** | ● | ● | ● | ● | ● | ● |  | ● | ● |  | ● | ● |
| **Fanning et al. [14]** | ● | ● | ● | ● | ● | ● | ● | ● | ● | ● | ● | ● |
| **Goode et al. [7]** | ● | ● | ● | ● | ● | ● | ● | ● | ● |  |  | ● |
| **Haas et al. [18]** | ● | ● | ● | ● | ● | ● | ● | ● |  |  |  | ● |
| **Hasegawa et al. [31]** | ● | ● | ● | ● | ● | ● |  | ● |  |  |  | ● |
| **Hausmann et al. [8]** | ● | ● | ● | ● | ● | ● | ● | ● |  |  |  | ● |
| **Hurley et al. [3]** | ● | ● | ● | ● | ● | ● | ● | ● | ● |  |  | ● |
| **Janevic et al. [9]** | ● | ● | ● | ● | ● | ● | ● | ● | ● |  |  | ● |
| **Jessep et al. [4]** | ● | ● | ● | ● | ● | ● | ● | ● | ● |  |  | ● |
| **Kwok et al. [26]** | ● | ● |  | ● | ● | ● | ● | ● | ● |  |  | ● |
| **Li et al. [10]** | ● | ● | ● | ● | ● | ● | ● | ● | ● |  |  | ● |
| **Morone et al. [21]** | ● | ● | ● | ● | ● | ● |  | ● |  |  |  | ● |
| **Morone et al. [22]** | ● | ● | ● | ● | ● | ● |  | ● |  |  |  | ● |
| **Morone et al. [24]** | ● | ● | ● | ● | ● | ● | ● | ● |  |  | ● | ● |
| **Nicholas et al. [23]** | ● | ● | ● | ● | ● | ● | ● | ● |  |  |  | ● |
| **Rini et al. [5]** | ● | ● | ● | ● | ● | ● | ● | ● | ● |  |  | ● |
| **Tse et al. [6]** | ● | ● | ● | ● | ● | ● | ● | ● |  |  |  | ● |
| **Vitiello et al. [19]** | ● | ● | ● | ● | ● | ● | ● | ● |  |  | ● | ● |
| **Walsh et al. [32]** | ● | ● | ● | ● | ● | ● | ● | ● | ● |  | ● | ● |
| **Weiner et al. [15]** | ● | ● | ● | ● | ● | ● | ● | ● | ● |  |  | ● |
| **Yarns et al. [33]** | ● | ● | ● | ● | ● | ● | ● | ● |  |  |  | ● |
| ● = reported; blank cells = not reported | | | | | | | | | | | | |

**Table 5. Intervention delivery and mechanism matrix**

|  | **Aim of intervention** | **Mechanism of change (theoretical / conceptual framework)** | **Intended delivery (programme length and frequency, staff training and input)** | **Actual delivery (difference from the intended delivery)** |
| --- | --- | --- | --- | --- |
| **Andersson et al. [28]** | To reduce chronic pain in older people. | CBT is an established treatment approach in the management of chronic pain in adults. This study's intervention was different from many previous CBT studies in that it did not include any cognitive restructuring or strictly operant techniques. | Protocol or intervention plan unavailable. | 6 weekly group sessions, 2 hours long with 15-minute break, delivered by two psychologists supervised by the first study author. Each session included CBT exercise, homework assignments, feedback, rationale, and written handouts. Venue and group size not reported. |
| **Baird and Sands [29]** | To reduce pain and mobility difﬁculties in women with osteoarthritis. | Verbal suggestions in GI guide the individual to create a flow of thoughts which may refocus attention on imagined sensations, initiate cognitive processes, block painful stimuli transmission, leading to specific psychological and physiological responses, e.g., relaxation. PMR reduces response to stress and skeletal muscle contraction, thus decreasing the sensation of pain. The complexity of the physiologic and psychological pathways by which GI with PMR reduces pain is unknown. Yet, PMR is an essential adjunct to GI as it causes both physiological and psychological relaxation thus decreasing the sensation of pain. | Protocol or intervention plan unavailable. | A personalized audiotape with instructions in using GI with PMR tailored for each participant. For writing the audiotape script, in the ﬁrst face-to-face meeting the staff collected details from the participants about the joints and movements that caused pain and they were asked to describe a relaxing place. Twice a day for 12 weeks, participant listened to a 10-to-15-minute audiotaped script while following the instructions. Staff called each participant fortnightly to discuss and address difﬁculties in using GI with PMR. Presumably the participants would choose the venue, e.g., home. Unclear who provided the intervention, and whether a tape player was provided to the participants. |
| **Bearne et al. [2]** | To mobilize the hip joint and strengthen the surrounding muscles, and to decrease chronic hip pain and disability. | A combination of physical exercise and self-management, which includes education, cognitive restructuring, and psychosocial interventions, reduces pain and improves function (by improving motor function). This type of rehabilitation programme is designed to be delivered in primary healthcare settings to people with chronic musculoskeletal pain. | Protocol or intervention plan unavailable. | 10 group sessions, twice a week for 5 weeks, each session lasting 75 minutes, up to 8 persons per group, delivered by a qualified clinical physiotherapist in a primary care hospital physiotherapy outpatient department.  Supervised exercises: 45-minute circuit of exercises. The physiotherapist prescribed exercises for each participant according to their abilities, and monitored and revised the performance of these exercises. Education, coping and self‐management: following each exercise session, 30‐minute ‘interactive discussion’ facilitated by the same physiotherapist. A handbook containing information about the discussion topics and exercises was provided. Following all group sessions, the participants were discharged with written instructions to perform a simple home exercise programme consisting of the exercises performed during their rehabilitation sessions. |
| **Berman et al. [11]** | To deliver self-care tools to older people via the Internet, and to document changes in pain and ability to manage chronic pain. | Online self-care interventions addressing pain due to a variety of conditions are associated with reduced pain, disability, catastrophizing of pain, healthcare utilisation, and improved control over pain, coping, role function, mood, and perceived stress. Delivering self-care education via the internet may overcome the logistic barriers of attending face-to-face sessions and benefit people in chronic pain who are isolated or who have difficulty leaving the home or who are reluctant to participate in a group. | Protocol or intervention plan unavailable. | Orientation to the intervention website and access details, provided in person by a research assistant (RA), at a location of participant's choice, e.g., home. During the 6-week intervention period participants were instructed to use the web site intervention at least once a week on their own schedule and at their own pace at a location of their choice. The RA monitored participants’ use of the website, sent emails to prompt individuals to complete the unvisited modules, and provided technical assistance. Materials included audio, visual, and textual components, with illustrative examples and worksheets. The content was written at an average Flesch-Kincaid grade level of 8.5.  A study nurse monitored participants’ levels of pain via an online self-assessment tool and contacted the participants to advise seeking medical attention. |
| **Broderick et al. [25]** | To teach patients cognitive strategies and behavioural skills to reduce the effects of chronic pain on functioning and quality of life. | PCST teaches patients cognitive strategies and behavioural skills to reduce the effects of chronic pain on functioning and quality of life. PCST aims to treat pain caused by a medical condition, and the interaction of emotional state and the pain experience is a fundamental component. Emerging literature supports nurse-led self-management interventions to enhance the individual's ability to manage chronic pain. Patients identify nurse care managers as strong sources of support through their approach to individualizing self-management strategies, holding patients accountable for their pain management, and motivating patients. The nursing profession, with its emphasis on implementing self-management and patient education counselling for chronic illness, is well suited to deliver PCST. | Protocol or intervention plan unavailable. | 10 sessions, once a week for 10 weeks, 30-45 minutes long, first 3 sessions and the last session conducted in person at the clinic which the participant attended, up to 4 sessions could be conducted via telephone. Patients were provided with a treatment manual with handouts and logs to record home practice of the skill.  Intervention delivered by adult health nurse practitioners in community primary care and rheumatology offices, hired by the research grant, who were provided a 2-day training workshop in PCST conducted by clinical members of the research team and continued training practice with the instructors at their site to reach competency with delivery of the treatment. |
| **Carmody et al. [30]** | To facilitate adjustment to chronic pain by managing negative emotions and maladaptive thought patterns, improving social functioning, and improving coping with stressful life events. | The stress-appraisal coping model of pain [102] suggests that individuals’ thoughts have a direct impact on their adjustment to chronic pain by means of their appraisal of the pain and related stressors, their beliefs regarding their abilities to control their pain, and their choices of coping strategies. CBT has been shown to be a key component in interdisciplinary pain-management programmes. A telephone-delivered version of CBT overcomes barriers related to in-person access and improves attendance and completion of the intervention. | Protocol or intervention plan unavailable. | 12 telephone sessions over 20 weeks: Sessions 1-8 weekly, Sessions 9-10 scheduled biweekly [i.e. every two weeks], Sessions 11-12 scheduled a month apart. 6 sessions each for the CBT method and skills training. Structure for each session: setting an agenda, reviewing task assignment, feedback from the patient, summary of key points, and tailoring task assignments. Participants were provided with handouts for each session to support the intervention and guide outside task assignments.  Delivered by therapists who received 20-hour training of didactic explanations of the intervention principles, role plays and feedback, and basic skills for telephone therapy; audiotapes for adherence and competence ratings were used to monitor adherence and competence; therapists attended a weekly supervision group led by the supervising research psychologist. Unclear whether all 4 therapists delivered both intervention and control sessions or how many delivered the intervention only. |
| **Cheng et al. [20]** | To improve aerobic capacity and muscle strength and endurance; to reduce pain and psychological distress; thus to increase self-efficacy for pain management. | Exercise programme for chronic musculoskeletal pain aims to improve the individual’s aerobic capacity, muscle strength, and endurance. Increased frequency of any types of exercises is found to be more essential in improving chronic musculoskeletal pain than the length or intensity of the activity. CBT aims to increase self-efﬁcacy for pain management and by addressing dysfunctional beliefs and maladaptive behaviours to improve pain and psychological distress. Non-pharmacological interventions are suitable for the long-term management of chronic musculoskeletal pain. Exercise therapies and CBT are particularly recommended and have been found to be effective in alleviating pain intensity and pain interfering with daily functioning. Hence, an intervention that combines both approaches is recommended for chronic low back pain. | 10 group sessions, once a week for 10 weeks, 90-minute long, in group of 6-12 participants, in a quiet room in the clinic or a social centre. Each session began with a 45-minute physical exercise programme; followed by 40 minutes of training on cognitive-behavioural concepts and techniques and a 5-minute conclusion. Each session was structured similarly and included a review of the previous session, homework review, mood checking, session objectives, discussion, skills practice, and homework/goal setting. A brochure with the session's content was provided to each participant at the end of each session. Delivered by a research assistant, who was trained and under the supervision of two research team members with backgrounds in clinical psychology and nursing. | Comparing the intervention details in the protocol and results report, no modification to the intervention was reported. |
| **Costantino and Romiti [16]** | To improve the recovery of lumbar region mobility and in terms of pain reduction and disability. | European Guidelines for the management of chronic non-specific low back pain recommend the inclusion of cognitive behavioural therapy and a short educational intervention in addition to exercise programmes to reduce pain intensity and related disability. Back School is an educational programme that provides individuals with practical information about back care, posture, body mechanics, back exercises, and how to prevent long-term back problems. Modern Back Schools originated from the synthesis of several rehabilitative physical therapies and are enriched by modern kinesitherapy techniques. They emphasise psychophysical preparation and the individual's goals or needs, well-being and ability to manage pain with confidence and commitment. | Protocol or intervention plan unavailable. | 24 sessions, 2 sessions per week over 12 weeks, each lasting one hour. Session 1: participants were informed about pain-inducing mechanism, psychological aspects and stress management. Sessions 2-24, participants performed stretching and muscular strengthening exercises, associated with proper breathing. Equipment for exercises: small cushion.  Delivered by a professional physiotherapist.  Venue is not reported; unclear whether sessions were held for group or individual. |
| **da Silva et al. [17]** | To prevent back pain and rehabilitate individuals with degenerative spine disorders. | Postural strain, prolonged immobilization, or poor body mechanics may lead to spinal alignment problems and muscle shortening, causing back pain. Improved flexibility, movement habits, and posture can alleviate back pain and maintain good posture and balance. The educational lessons provide theoretical and practical information comprising spine anatomy, biomechanics, optimal posture, ergonomics, and exercises. Hence, back school programmes facilitate individuals to become responsible for their own recovery process and for the maintenance of their quality of life. | Protocol or intervention plan unavailable. | 10 sessions, twice a week for 5 weeks. The programme comprised educational lessons (theoretical concepts and practical training), physical exercises (dedicated to the maintenance of a “healthy back”), and relaxation sessions (massage, myofascial release, and trigger point therapies on trunk muscles), of approximately 30 minutes each. Pamphlets were provided to participants relating to the content of the sessions. Educational lessons and physical exercises were delivered by a therapist (physical therapy students / physiotherapists). Venue and whether sessions were delivered in group or individual were not reported. |
| **Ersek et al. [12]** | To enhance the ability to self-manage pain, hence to improve pain, mood, and physical functioning. | Enhanced beliefs in control and self-efficacy are found to be associated with improvement in pain outcomes among young-old people. Pain coping skills training can facilitate older people to increase perceived control over pain and decrease catastrophizing, thus reducing physical disability. | Protocol or intervention plan unavailable. | 7 90-minute group sessions: sessions 1-6 held weekly, session 7 held 2 weeks after Session 6. Each session included the presentation and discussion of various topics including chronic pain, relaxation practice, and developing an activities plan. Delivered by 1 of 2 trained, doctoral-level health professionals at the retirement community facilities. The first group meeting was co-facilitated by both professionals. They met after each session to discuss group processes and adherence to the session protocol. Group size is not reported. |
| **Ersek et al. [13]** | To decrease participants' physical disability and pain intensity; to increase participation in home, social, and recreational activities; and to enhance their self-efficacy for managing chronic pain. | Multimodal therapies that incorporate cognitive and behavioural strategies aim to enhance the participant's ability to successfully self-manage pain. In the self-management approach, the individual actively participates in treatment, consisting of the five core self-management skills: problem-solving, decision-making, resource utilization, patient-provider partnership, and adoption of actions. A distinguishing characteristic of self-management is tailoring the intervention to the individual [103]. Adoption of regular wellness-oriented pain management strategies may contribute to enhanced functioning and prolonged independence in older people. | 7 90-minute group sessions, one per week. Each session included the presentation and discussion of various topics including chronic pain, relaxation practice, and developing an activities plan. An intervention facilitator telephoned each participant at 12, 16, 22, and 30 weeks after the final group session to discuss pain and functioning, current pain management plans, and successes and obstacles in meeting pain management goals, and provide encouragement. Calls lasted 5-20 minutes.  Delivered by 1 of 3 trained health professionals (two nurses and one clinical psychologist) at the retirement community facilities. They were specifically trained according to the treatment protocol and met 3 times to discuss protocol and treatment integrity issues. All sessions were audiotaped, 20% were selected to be reviewed for protocol adherence and any deviations were discussed with the facilitator. Group size is not reported. | A random sample of 20% of the audiotaped sessions were reviewed for protocol adherence. Over 95% of items that were covered were in accordance with the protocol. |
| **Fanning et al. [14]** | To decrease both body mass and sedentary behaviour; to reduce chronic pain and improve physical functioning in older people. | Intervention content is grounded in social cognitive theory [104] which promotes self-efficacy and social outcome expectancies to facilitate modifying the challenging behaviours. The design of the smartphone app (MORPH Companion app) is grounded in the Ritterband model for Internet interventions [105], which proposes nine factors and nonlinear steps for effective internet behavioural change interventions, including the users, environmental factors, and website characteristics. Higher body mass index (BMI) is associated with increased pain severity, and both are associated with decreased physical performance, social and physical activities in older people. Group-based behavioural intervention delivered via smartphone and mHealth devices may increase the accessibility of the intervention for older people whose transport and social activities are limited. Weekly meetings and daily smartphone tools cue mastery experiences, enhance self-regulation, and provide ongoing support. Cueing brief exercises alongside light-to-moderate movement throughout the day and before peaks in pain and affective lows may support lasting behaviour change, reduce sedentary behaviour and thus reduce weight and improve pain. | 12 group sessions, once a week for 1 hour. Sessions 1-3: face-to-face meetings, provided equipment (MORPH Companion mHealth package: MORPH Companion App installed on mobile phone, Fitbit Alta wearable activity monitor, BodyTrace cellular-enabled weight scale, Webex teleconferencing software suite) to participants; and focused on orientation to the intervention and education. Weekly call between each participant and the behavioural interventionist to practice use of the Webex software. Sessions 4-12: online meetings, education provided by the interventionist and nutritionist, group discussion on successes and barriers in meeting goals, and practise guided mindfulness skills. During the intervention period, physical activity levels and weight data collected from the equipment were linked to the App for self-monitoring by participants and monitoring by interventionists of weight, sedentary behaviour, step counts and the dietary diary record. Step count and dietary intake goals were reviewed and set weekly by the interventionists via the App. Weekly animated videos reinforced weekly educational content, and participants also received regular messages within a “newsfeed” feature from the behavioural interventionist. Participants were able to reach intervention staff by phone or email throughout the intervention.  Group size and face-to-face meeting venue are not reported. | Device failure (e.g., battery failure, Bluetooth pairing failure) was experienced, and user errors while using the equipment were reported (e.g., placing the device on the charger and then forgetting to wear it, losing the device, failing to charge the device). |
| **Goode et al. [7]** | To treat chronic lower back pain in older people. | Physical activity can significantly improve physical function among older people and can be successfully delivered in the home setting. CBT for pain (CBT-P) has been shown to improve functional outcomes when combined with exercise in people with chronic lower back pain. Older people may benefit from remote telecommunication delivery of healthcare as a way of accessing services from homes. | Protocol or intervention plan unavailable. | 13 telephone call sessions over 12 weeks: physical therapist, weeks 1, 4 and 9; exercise counsellor, weeks 1-3, 5-8, and 10-12. Approximately 15 minutes for each call.  The exercise counsellor helped the participant to develop and refine action plans for achieving physical activity goals. The physical therapist provided an educational introduction in the first session and in all 3 sessions assessed any potential issues as a result of the delivered intervention (e.g., increased pain) and recommended necessary modifications in the exercise program. |
| **Haas et al. [18]** | To reform the participants’ expectation beliefs regarding their capability to exert control over their own behaviour. | Self-efficacy enhancing self-management posits that the individual has an active role in managing and improving health behaviour and outcomes. By reforming the beliefs in one's capabilities to exert control over one’s behaviour and actions, self-management may improve the individual's health status, quality of life, and reduce healthcare usage. Based on an interactional model of human behaviour, trained volunteers who suffer the same condition serve as role models to the participants. | Protocol or intervention plan unavailable. | 6 group sessions, 1 per week, 2.5 hours each, delivered in workshop class format and included a lecturette supplemented with a book, a period of group interaction, and a period of team tasks, implemented in 12 geographical locations, including community organisation and centres. Delivered by 2 trained lay leaders (volunteers) in a small group format. The leaders served as role models, as they also lived with chronic back conditions, and encouraged participants, through examples and discussions, to develop the confidence necessary for taking greater control of their health. Participants were contacted by phone every 2 weeks for 24 weeks with workshop reminders and to maintain contact. Group size and training for volunteering lay leaders were not reported. |
| **Hasegawa et al. [31]** | To correct and decrease the pain by addressing the psychological aspects of chronic pain. | In attentional bias modification, language or facial expressions are displayed on a screen (threat stimulus and neutral stimulus) to train responses away from painful expression by selecting a neutral stimulus, thus correcting the bias of attention caused by the emotional value of the stimulus. | Protocol or intervention plan unavailable. | 24 sessions, 2 sessions per week over 12 weeks, each lasting 10 minutes approximately. Using attentional bias modification Trainer for Windows (ideoquest, Tokyo, Japan) to randomly display images (human expressions including threat and neutral stimuli) on the top and bottom of a computer screen. Participant were instructed to select the neutral stimulus as quickly as possible. A rater was in a room separate from the participant.  The staff members and venue were not reported. |
| **Hausmann et al. [8]** | To build positive psychological skills to reduce osteoarthritis symptom severity, including pain and functioning, and to improve psychosocial wellbeing in patients with knee or hip osteoarthritis. | The biopsychosocial model of pain [106] recognises that a complex interaction of biological, psychological, and social factors influences an individual's pain experience. Poorer psychological characteristics and adverse social factors are associated with worse outcomes among people with osteoarthritis, and the psychosocial factors interact and affect osteoarthritis outcomes. Building positive psychological skills and improving psychosocial well-being may improve pain symptoms and positive affect for people with osteoarthritis. | Protocol or intervention plan unavailable. | 7 sessions, including orientation at baseline and 1 telephone session per week over 6 weeks, each telephone call lasting 10-15 minutes. At orientation, the study staff provided general orientation to the programme workbook, and reviewed the instructions for the first week’s activity. During each week, participants completed the week’s activity and study staff telephoned to assess adherence to the activity and review instructions for the next week’s activity. Details of staff training are not reported. |
| **Hurley et al. [3]** | To improve functioning, understanding, and conﬁdence in order to dispel inappropriate health beliefs, alter behaviour, and encourage regular physical activity. | Improvement in function is best achieved via benefits attainable from exercise and education/self-management interventions. Exercise and self-management complement each other and may enhance the benefits to be gained from each. The possible mechanisms are that simple exercises and activities without painful exacerbation, coupled with information, advice, and reassurance from a knowledgeable health care professional, may increase participants’ understanding of the importance and beneﬁt of physical activity, restore conﬁdence in their abilities, and facilitate self-help, thus resulting in changes in exercise health beliefs and self-efﬁcacy. | Protocol or intervention plan unavailable. | Delivered either to individuals in the Indiv-rehab arm, or via a group (average size of 8) to those in the Grp-rehab arm. 12 sessions, 2 sessions per week over 6 weeks, each session lasting approximately 45 minutes (Indiv-rehab arm) or 60 minutes (Grp-rehab arm). The intervention comprised interactive self-management, coping and education and discussion about self-care for arthritis (first 15-20 minutes) and an individualised progressive exercise programme (35-45 minutes). The physiotherapist and each participant agreed and reviewed the exercise complexity and intensity progression and developed a personalised action plan and home exercise regimen. After completion of all sessions, each participant was discharged with specific advice and written instructions to perform a 15-minute home exercise programme three times a week. Sessions to individuals could be rescheduled but not group sessions. A single physiotherapist delivered all the sessions to both arms at a Physiotherapy Outpatient Department in a community hospital. Average non-contact time (prepare session, write up notes) associated with each person for each session was 30 minutes. Did not require specialized training, sophisticated exercises, equipment, or facilities, but details are not reported. |
| **Janevic et al. [9]** | To address the disproportionate burden of pain-related disability among African American older people. | Broaden and Build model posits that more opportunities for positive affect will enhance and build resilience, facilitate learning new skills, and invest in relationship [107]. Chronic pain self-management interventions include psychological and behavioural skills and are recommended as part of chronic pain treatment to improve functioning and quality of life while avoiding medication-associated risks. Positive activity interventions based on the Broaden and Build theory attempt to induce positive emotions through simple, enjoyable activities and improve pain outcome via various pathways. These processes can help older people living in chronic pain to counteract the sense of helplessness and low mood. | Protocol or intervention plan unavailable. | 8 sessions, including orientation at baseline and 1 structured session per week over 7 weeks, each lasting 30 minutes. All sessions delivered over telephone by a designated Community Health Worker who reinforced key messages of the weekly topics, discussed the participant’s activity patterns and barriers, formulated action plans and set pain self-management goals with participants, and made referrals to community resources if necessary. Between sessions, participants watched web-based videos, and/or read the weekly topics in the workbook or website, and completed activities. The intervention was delivered remotely; resources including activity tracker and programme materials were mailed to participants or provided online.  3 Community Health Workers received a half-day training to deliver the intervention. They had previously completed the Michigan Community Health Worker Alliance training, which teaches core competencies including communication skills, healthy lifestyle components, and legal and ethical responsibilities.  Differences from intended delivery: participants often had difficulties accessing site, connectivity issues, using the tracker. |
| **Jessep et al. [4]** | To change people’s behaviour by challenging inappropriate beliefs regarding their condition and physical activity, encouraging regular exercise and enabling self-management of symptoms. | "The ethos of ESCAPE-knee pain is that regular exercise can control the symptoms and effects of chronic knee pain." Self-management skills can improve adherence to regular exercise and thus sustain the therapeutic benefits of exercise. The intervention programme challenges people's inappropriate beliefs about their condition and physical exercises, encourages regular exercise, and enables self-management via group discussion, coping strategies, problem-solving and planning, regular progressive exercise, and ongoing support from the review session. | Protocol or intervention plan unavailable. | 10 group sessions, 2 sessions per week over 5 weeks, each session lasting about 1 hour. Each session was delivered by a physiotherapist at a local authority adult education centre, including an informal themed group discussion (first 15-20 minutes), followed by a 40-minute progressive exercise circuit. Participants were provided with written information summarising key messages from each session. 4 months after completion of the programme, the physiotherapist telephoned participants to invite them to a 1-hour review session, and the participant’s home exercise regimen was reviewed.  The physiotherapist delivered all the sessions; training only included information about the programme’s ethos, aims, structure and content, guiding group discussions, and observing some groups being held at another location.  Group size not reported. |
| **Kwok et al. [26]** | To improve quality of life via improvement in pain level and frequency and physical functioning, and to raise the level of self-efficacy. | Lorig & Holman [103] suggested that self-efficacy is a possible change mechanism, based on Bandura's [104] Self-Efficacy Theory. Older people interpret pain as an inevitable part of ageing. However, positive pain outcomes may result from improved support for self-management and adherence to health regimens. Lorig and Holman's self-management concept and approach is a process of learning and actioning skills to manage the impact of a person's chronic conditions, by providing information, teaching problem-solving techniques, and changing a person's beliefs. | Protocol or intervention plan unavailable. | 6 group sessions, 1 session per week over 6 weeks, each session lasting 2 hours, in group of 6-7 persons. The sessions were interactive, included short lectures, group discussions, problem solving role plays, and skills practice; delivered by a researcher in a mobile integrative health centre (a vehicle equipped with medical facilities) located in areas where the participants resided.  The training for staff was not reported. |
| **Li et al. [10]** | To help reduce pain symptoms, improve quality of life, develop good exercise habits, cope with and break the vicious circle of pain. | Based on the Theory of Dyadic Illness Management [108] which focuses on the dyad as an interdependent team: they interact while co-participating in the intervention. Interventions targeting informal caregivers can improve their knowledge and coping skills to facilitate their caring experience and roles positively. Physical exercise and educational interventions for pain relief among community-dwelling older people can signiﬁcantly reduce their pain, enhance their physical function, and improve their well-being and self-eﬃcacy. Increased pain self-eﬃcacy means that elderly people have more conﬁdence in managing their pain-related symptoms, stresses, or limitations. Pain education helps older people to understand their responses to pain thus leading to a reduction in pain. | Protocol or intervention plan unavailable. | 4 dyadic sessions, 1 session per week over 4 weeks of 1 hour each, followed by home-based and digital-based activities for 4 weeks. The 4 sessions were delivered face-to-face to each dyad by a researcher at local community activity centres, each included watching a 10-minute video and discussion, 20-minute physical exercises, 20-minute pain management education, and 10-minute questions and answers conclusion. A book to guide home-based exercises was given to each dyad at the Week 4 session for practice in Weeks 5-8. During the 8 weeks, participants and researchers communicated via a mobile phone messaging app; videos and topic materials were shared to participants via the app.  Notes: Use of the messaging app was not reported in the trial register. 10-minute video watching and discussion and 10-minute conclusion parts in each session were only reported in trial register. |
| **Morone et al. [21]** | To improve pain, physical function, and quality of life. | The acceptance-based approach is based on Kabat-Zinn's [109] concept of mindfulness which encourages participants to take a fresh look at their pain condition and emphasises the concepts of letting go of struggle and accepting one’s condition without judgment, which may result in improvement on the experience of pain. | Protocol or intervention plan unavailable. | 8 group sessions, 1 per week over 8 weeks, each 90 minutes long. Each session included 45 minutes meditation and 45 minutes discussion. Support materials (audiotape, daily diary and reading materials) were provided. The audiotape was a 45-minute recording for guided body scan meditation and a 30-minute recording of guided sitting meditation, for weekly meditation homework assignments.   All sessions were led by 2 experienced MBSR teachers who had both received training in an MBSR program for health professionals directed by staﬀ from a Center for Mindfulness (such as Kabat-Zinn).  Group size and venue were not reported.  Notes: Scheduling conflicts of participants adversely impacted on recruitment and continued participation. |
| **Morone et al. [22]** | To decrease pain-related disability and pain. | Mindfulness meditation is well-suited to people with chronic pain as it teaches them to cope positively with pain which is experienced through sensory, cognitive, and affective domains. It is “*the awareness that emerges through paying attention on purpose, in the present moment, and nonjudgmentally to the unfolding of experience moment by moment*” [109]. Mindfulness methods for pain reduction include distraction, increased body awareness leading to behaviour change, better pain coping skills, imagery, and direct pain reduction through meditation. Benefits may take time to occur and regular meditation practice is emphasised to develop familiarity with the methods. | Protocol or intervention plan unavailable. | 8 group sessions, 1 per week over 8 weeks, each 90 minutes long. Each session included 1 hour of meditation and 30 minutes of discussion. Support materials (CD recording, daily diary and reading materials) were provided to support homework of daily meditation (45 minutes practice, 6 days per week). The CD recording was a 45-minute guided body scan meditation and a 30-minute guided sitting meditation; CD player was provided if needed by participants. First session introduced mindfulness meditation principles and practice. Sessions 2-8 included 30-minute discussion about meditation experience and meditation skills and practice.   All sessions were led by an experienced MBSR Program teacher who had received at least 50 hours of teacher training in the MBSR programme.  Group size and venue were not reported. |
| **Morone et al. [24]** | To increase function and decrease pain, and improve neuropsychological performance. | Mind-body therapies such as mindfulness meditation teach participants to work with the sensory, cognitive, and affective responses to pain. Mindfulness meditation in ordinary activities is a simple and safe method for improving function and pain relief and is suitable for the frailest older adult. It can affect pain processing by possibly activating the top-down inhibition of a nociceptive input. The sensory domain may be affected by relaxation and reduced muscle tension and thus reduction in pain sensation. Improved coping with negative affective reactions and enhanced general well-being may have an effect on the affective domain of pain processing. Recognising habits or thoughts, or improving attention and executive function performance may affect the cognitive domain of pain response. | 8 group sessions, 1 per week over 8 weeks, each 90 minutes long; followed by 6 booster sessions, 1 per month for up to 12 months, each about 1 hour long; 10-12 persons per group. First session introduced mindfulness meditation principles and practice, and homework of daily meditation (45 minutes practice, 6 days per week), provided CD recordings of guided meditations, daily log and reading materials to participants. Sessions 2-8 included 30-minute discussion about meditation experience and mediation skills and medication practice. Booster sessions were offered to participants who had completed the 8-week programme, including 30–40 minutes of mindfulness meditation and 20–30 minutes of discussion around the 8-week programme.   All sessions were led by an experienced MBSR Program teacher who underwent at least 2 intensive training programmes (≥ 100 hours) provided by the University of Massachusetts Medical School Center for Mindfulness; the sessions took place in a large classroom in a university campus, easily accessible by public transport. If a participant missed a session, the teacher would call them to review session materials relevant to the participant and encourage continued participation. | Slight differences about when some weekly content was delivered.  Group size was 10 persons.  Booster sessions were provided for 6 months. |
| **Nicholas et al. [23]** | To improve pain management skills, with consequently less suffering and the restoration of a more normal lifestyle despite persisting pain. | Self-management approaches emphasise the person taking an active role in managing their condition. CBT principles used in chronic pain self-management target changes in both cognitions and behaviours. Cognitive-behavioural theoretical formulations have posited that CBT for pain achieves improvements partly by reducing unhelpful cognitions. One of the tenets of CBT interventions for pain is that the impact of pain can be changed through cognitive mediating factors, e.g., pain self-efﬁcacy beliefs, catastrophising and fear of activities leading to disabilities. | Protocol or intervention plan unavailable. | 8 group sessions, 2 sessions per week over 4 weeks, each of 2 hours long (every Tuesday and Thursday, 10 am to noon), approximately 8 persons per group. Each session included discussion and practice of exercises and skills. Self-monitoring of homework was encouraged and evidence of attempts to practice the exercises and to apply the skills at home were reinforced with praise by the treatment team. Each participant was given a copy of the self-management “Manage Your Pain” book and encouraged to read it and use it as the treatment manual and the session topics were based on it.  A psychologist delivered the content of psychological sessions and a physiotherapist was responsible for the exercise sessions, both were present throughout each session at the Pain Management and Research Centre of a principal tertiary hospital.  Staff training details were not reported. |
| **Rini et al. [5]** | To reduce pain and improve pain-related interference with functioning, pain-related anxiety, self-efficacy for pain management, and positive and negative affect. | Based on the social cognitive theory [104], adult learning theory [110], and multimedia instruction principles, pain coping skill training focuses on educating people about links between cognitive, emotional, and behavioural responses and the pain coping skills, and helping people master those skills in pain self-management.  Motivational interviewing techniques enhance participants' motivation to initiate, adhere, and complete the programme. An “expert systems approach” is used in the programme algorithms which customise the modules and features according to participants’ responses and progress. | Protocol or intervention plan unavailable. | 1 in-person meeting at baseline and an 8-week internet-based intervention programme comprising 8 modules. At the baseline meeting, the research staff member used motivational interviewing techniques to ask questions designed to enhance participants’ motivation to complete the entire PainCOACH programme; the baseline meeting took place at a university medical centre or research centre. For the online programme, participants were expected to complete 1 module per week in a self-directed manner (i.e. without therapist contact). Participants were led through the programme by a female “virtual coach”. Each session took 30-45 minutes to complete, and included text, illustrations, photos, animations, audio narration, and provided interactive training and personalised feedback. Participants practised the skills after learning them and their completion of and experiences with practices were reviewed in the next module.  The modules and features were controlled by programming that used decision rules to customize participants’ experience based on their responses and progress through the programme, using an “expert systems approach” and algorithms. Participants accessed the programme at home through a wireless high-speed broadband connection or a 4G LTE high-speed Internet connection provided by the study team. Tablet computers were loaned to participants if required. Staff called participants to verify accessibility to the online programme and if a module was not accessed in time.  Staff who conducted the motivational interviewing at baseline were trained but the details of training were not reported. |
| **Tse et al. [6]** | To achieve pain reduction and improvements in physical and psychological well-being, self-efficacy and quality of life among older persons in the community with pain. | Motivational interviewing is a client-centred counselling technique which aims to improve the individual's motivation and commitment to initiate, perform and maintain behavioural changes. It empowers and encourages the individual in active decision-making to gain control over their own lifestyles and health, thus increasing self-efficacy in the self-management of pain. Physical exercise has been shown to reduce pain intensity and improve physical function and psychological well-being. Integrating motivation enhancement and an exercise programme, while addressing older people's motivation and barriers to physical activities and pain management, can decrease pain intensity and increase function. | Protocol or intervention plan unavailable. | 8 group sessions, 1 session per week over 8 weeks, 1.5 hours long for each session, held in community centres. The programme consisted of two main components: motivational interviewing counselling and physical exercise. The 30-minute MI session was held weekly in groups of 8-10 persons, led by a motivational interviewing -trained physiotherapist; participants shared pain experience and practised skills. The 45-minute physical exercise session was held weekly in groups of 15-20 persons, led by a physiotherapist and assisted by registered nurses. A booklet was given to participants to guide practise of the exercise at home (30 minutes every day or at least 4 times per week). A researcher telephoned each participant once during the intervention period to assess the compliance with exercise, boost adherence and check safety of self-practice.  Staff training details not reported. |
| **Vitiello et al. [19] CBT-PI** | To improve sleep and reduce pain and depression; hence increase or maintain physical and cognitive function. | Based on a bio-behavioural model of chronic pain dysfunction [111] and sleep disturbance, daily activities and sleep schedule are re-regulated through the enhancement of behavioural and coping responses to osteoarthritis pain and sleep disturbance thereby improving sleep which engenders more positive emotions and cognitions and reducing pain, resulting in increased or maintained physical and cognitive function. | 6 group sessions, 1 per week over 6 weeks, approximately 1.5 hours in duration each. Delivered in-person by a pair of mental health professionals (interventionists), at the Group Health primary care clinics where participants received primary care. A study assistant scored each participant’s weekly sleep diaries at the beginning of each session to facilitate their use by the interventionists to guide individualized treatment.  Staff training details were not reported. | Participants were enrolled in sessions according to their preferred start date.   Group size range 5-12 persons, average of 9.4 persons per group.  Sessions were delivered in a classroom setting at the primary care clinics. |
| **Vitiello et al. [19] CBT-P** | To reduce pain, and improve functions and cognition. | Based on a bio-behavioural model of chronic pain dysfunction [111], daily activities are re-regulated by managing the behavioural and coping responses to osteoarthritis pain hence improving emotions and cognitions, and reducing pain. | 6 group sessions, 1 per week over 6 weeks, approximately 1.5 hours in duration each. Delivered in-person by a pair of mental health professionals (interventionists), at the Group Health primary care clinics where participants received primary care.  Staff training details were not reported. | Participants were enrolled in sessions according to their preferred start date.   Group size range 5-12 persons, average of 9.4 persons per group.  Sessions were delivered in a classroom setting at the primary care clinics. |
| **Walsh et al. [32]** | To improve function in people with chronic knee, hip, or lower back pain. | Regular exercise and activity potentially improve general health status and joint function. Improving weight and blood pressure can reduce the risk of other conditions among older people. Exercise, education and self-management are recommended in osteoarthritis and chronic, degenerative joint pain management guidelines. Based on social cognitive theory [57], self-management skills will support people to participate in regular exercise and manage problems with lower limb joints or the lower back through exercises. | 12 group sessions, 2 sessions per week over 6 weeks, each lasting 60 minutes, approximately 8 persons per group. Each session included 15-20 minutes of group discussion and problem-solving session (with supporting handouts) about self-management led by the physiotherapist; followed by 35-40 minutes of physical exercises. Each participant developed an exercise/activities action plan for the following week in collaboration with the physiotherapist, which was reviewed each week.  All sessions were facilitated by the same physiotherapist (Trial Co-ordinator), and were held at community-located physiotherapy out-patient departments, using the existing equipment in typical community-located physiotherapy out-patient departments (no additional equipment was required). Sessions were scheduled into standard working hours.  Staff training details not reported. | The group discussion was approximately 20-25 minutes long.  The length of exercises in each session was approximately 30-35 minutes.  Each participant was provided with a supplementary patient booklet that contained educational materials and self-completed tasks to monitor their progress. |
| **Weiner et al. [15]** | To evaluate and treat the common contributors to pain and disability in older adults with chronic lower back pain, thus reducing pain and improving function. | Chronic lower back pain in older people is posited to be a geriatric syndrome and a final common pathway for the expression of multiple contributors, e.g., co-existing extra-spinal disorders, depression. Therefore, degenerative disease of the lumbar spine is not the sole treatment target. A series of clinical algorithms targeting 12 common contributors (health conditions) were produced to guide a comprehensive structured evaluation and treatments of these co-existing conditions in older people with chronic lower back pain. | Protocol or intervention plan unavailable. | Aging Back Clinics care included 1) a structured history and physical examination to identify pain contributors, 2) structured participant education, 3) collaborative decision-making, and 4) care guided by condition-specific algorithms.  A geriatrician reviewed screening questionnaire results and accordingly tailored the structured history-taking and physical examination procedures for each participant, conducted in outpatient clinics of 2 Veterans Affairs Medical Centers.  Upon completing the assessment, an Aging Back Clinics provider educated the participant about the relevant chronic lower back pain contributors, provided a summary of assessment findings, and discussed a multifaceted approach to treatment guided by 13 published algorithms. The providers collaborated with participant to devise and agree a treatment plan. Frequency and length of intervention varied according to the individual's needs and agreed interventions.  The 3 Aging Back Clinics providers were geriatricians who had not received official training in pain medicine, but were trained to conduct structured physical examination procedures and use the treatment algorithms. |
| **Yarns et al. [33] EAET** | To reduce stress and resolve trauma and psychological conflict, hence to relieve pain. | The conceptual model for emotional awareness and expression therapy is that stress and unhealthy ways of dealing with emotions cause pain through alterations in brain structure and function. Encouraging the disclosure, expression, and processing of avoided emotions and engagement in healthy relational behaviours can affect the brain centres which process pain and emotion, reduce stress, resolving trauma and psychological conflict and thus relieve pain. | Protocol or intervention plan unavailable. | 9 sessions, 1 per week over 8 weeks, including an initial, individual orientation session and 8 group sessions in small groups of up to 8 persons, each lasting 90 minutes. The intervention was conducted using the treatment manual: the initial session included obtaining a pain history, presenting the treatment conceptual model, and developing a therapeutic alliance with the participant; group sessions included discussion, experiential exercises, and written homework. Delivered by licensed clinicians (therapists), assisted by trainees: one psychiatrist conducted all four groups of emotional awareness and expression therapy and was joined by a psychology intern for the first two groups.  Venue and staff training details were not reported.  Notes: most patients missed a few sessions, mainly due to scheduling challenges and group treatment (which precluded rescheduling) |
| **Yarns et al. [33] CBT** | To treat chronic pain psychosocially. | The conceptual model for cognitive behavioural therapy is that pain is chronic, but learning cognitive and behavioural skills can help to cope with or manage pain, functional loss, and emotional distress. | Protocol or intervention plan unavailable. | 9 sessions, 1 per week over 8 weeks, including an initial, individual orientation session and 8 group sessions in small groups of up to 8 persons, each lasting 90 minutes. The intervention was conducted using the United States S Department of Veterans Affairs CBT for chronic pain manual: the initial session included obtaining a pain history, discussion about pain management, and psychoeducation on cognitive and behavioural skills training; group sessions included discussion, experiential exercises and written homework. Delivered by licensed clinicians (therapists), assisted by trainees: 2 psychologists conducted the groups: one for the first three groups and the other for the fourth group, and they were joined by a geriatric psychology fellow.  Presumably, therapists received the national training in CBT for chronic pain through their Evidence-Based Psychotherapy Training Program. Venue was not reported.  Notes: most patients missed a few sessions, mainly due to scheduling challenges and group treatment (which precluded rescheduling) |
| **Keys:** CBT=cognitive behaviour therapy; GI=guided imagery; PMR=progressive muscle relaxation; PCST=pain coping skills training; MBSR=Mindfulness-Based Stress Reduction Program; CBT-PI= cognitive-behavioural therapy for pain and insomnia; CBT-I=cognitive-behavioural therapy for insomnia; EAET= emotional awareness and expression therapy | | | | |

**Table 6. Intervention details**

| Study | Intervention | Component | Delivery mode | | | | Notes |
| --- | --- | --- | --- | --- | --- | --- | --- |
|  |  |  | To individual: | | To group: | |  |
|  |  |  | in person | distantly | in person | distantly |  |
| **STAND-ALONE PSYCHOLOGICAL THERAPIES** | | |  |  |  |  |  |
| **1. Methods to enhance acceptance, mindfulness and psychological flexibility** | | |  |  |  |  |  |
| Hasegawa et al. [31] | Attentional bias modification, using facial expression-based stimuli. | Attentional bias modification trainer randomly displays images of human expressions of negative threat stimuli and neutral stimuli; individual is instructed to select the neutral stimulus as quickly as possible. |  | ● (1) |  |  | (1) Via computer at site hospital |
| **2. Skills training and activity management** | | | |  |  |  |  |
| Baird and Sands [29] | Guided Imagery with Progressive Muscle Relaxation. | Pain sensation, relaxation (guided imagery, progressive muscle relaxation) |  | ● (1) |  |  | (1) (by personalised audiotape and instructions) |
| Berman et al. [11] | An online mind-body self-care techniques pain management intervention for adults aged 55 years and older with chronic pain. | The Six Stages of Change (problem-solving approach to planning for change), abdominal breathing, relaxation, writing about positive experiences, writing about difﬁcult experiences, creative visual expression, and positive thinking. Reﬂection on responses to pain and action planning. | ● (1) | ● (2) |  |  | (1) orientation only  (2) via online website |
| Broderick et al. [25] | The Pain coping skills training treatment which promotes the use of cognitive-behavioural pain management coping skills for managing pain and enhancing perception of pain control. | Reducing negative pain-related thoughts and emotions, progressive muscular relaxation, mini practices of relaxation, and activity pacing, pleasant imagery and distraction. Homework assignments with review and problem-solving. | ● (1) | ● (2) |  |  | (1) Sessions 1-3 and 10  (2) via telephone, sessions 4-9 |
| Hausmann et al. [8] | Positive psychological intervention programme containing positive skill-building activities. | Write down three good things, expressing thanks, acts of kindness, making good moments last, increasing pleasant activity, practice favourite activities. | ● (1) | ● (2) |  |  | (1) Baseline orientation only  (2) Via telephone |
| Yarns et al. [33] CBT | CBT for chronic pain | Discussion of medical, behavioural, and other approaches for pain management, psychoeducation on cognitive and behavioural skills training, relaxation training, pleasant activity scheduling, cognitive coping, and distraction, sleep hygiene, review and future planning. | ● (1) |  | ● |  | (1) Orientation session |
| **3. Skills training and activity management + Education** | | |  |  |  |  |  |
| Haas et al. [18] | Chronic Disease Self-Management Program for chronic low back pain. | Skills training and activity management  Skills building from sharing and learning from others, goal setting, action plans, feedback, and problem-solving; strategies for managing pain, physical limitations, and symptoms.  Education  General principles of chronic conditions, overview of self-management principles, care-seeking options, community resources, exercise, relaxation, nutrition, medication and side-effects. |  |  | ● |  |  |
| **4. Skills training and activity management + Graded activation guided by participant goals** | | |  |  |  |  |  |
| Ersek et al. [12] | Pain self-management group intervention for elderly persons with chronic pain. | Skills training and activity management  Definitions and mechanisms of pain, pain self-monitoring, pharmacologic and non-pharmacologic therapies, decision making, relaxation training and exercises, and communication with healthcare providers.  Graded activation guided by participant goals  Develop individualised pain management goals: identify needs and goals, goal-setting, identify and problem-solve for obstacles, monitor and review progress. |  |  | ● |  |  |
| **5. Skills training and activity management + Methods to enhance acceptance, mindfulness and psychological flexibility** | | |  |  |  |  |  |
| Morone et al. [21] | Mind-body techniques for the treatment of chronic pain in older adults, based on Kabat-Zinn's mindfulness mediation programme (1990). | Skills training and activity management  Discussion to facilitate meditation practice, theoretical information about meditation, pain, stress and the mind/body connection.  Methods to enhance acceptance, mindfulness and psychological flexibility  3 techniques - body scan, sitting practice, walking mediation, which focus participant's attention on body sensation and breathing. |  |  | ● |  |  |
| Morone et al. [22] | Eastern methods of mindfulness meditation which transform regular daily activities into a meditation through directed breathing and mindful awareness. | Skills training and activity management  Discussions of experience with meditation, problem-solving, pain, triangle of awareness, relaxation and stress response to worsening or decreasing pain.  Methods to enhance acceptance, mindfulness and psychological flexibility  Practice mindfulness mediation methods: the body scan, sitting practice, and walking meditation, with focused attention on body sensation and/or breathing. |  |  | ● |  |  |
| Morone et al. [24] | Mind-body programme: a mindfulness meditation programme, modelled on the Mindfulness-Based Stress Reduction programme. | Skills training and activity management  Discussions of experience with meditation, problem-solving, pain, triangle of awareness, relaxation and stress response to worsening or decreasing pain, and breaking through habits.  Methods to enhance acceptance, mindfulness and psychological flexibility  Practice mindfulness mediation methods: the body scan, sitting practice, walking meditation, and mindful stretching, with focused attention on breathing and mindful awareness. |  |  | ● |  |  |
| **6. Skills training and activity management + Cognitive therapy methods** | | |  |  |  |  |  |
| Andersson et al. [28] | Group-based CBT with a focus on applied relaxation for older adults with chronic pain. | Skills training and activity management  Relaxation practice, and topics about sleep management, communication strategies, and assertiveness  Cognitive therapy methods  CBT for chronic pain |  |  | ● |  |  |
| Carmody et al. [30] | A telephone-delivered cognitive-behavioural therapy in the management of chronic pain. | Skills training and activity management  Skills training in relaxation, coping self-statements, expressive writing, assertive communication, maintaining change, coping with setbacks, and overall review.  Cognitive therapy methods  The stress-pain-judgment model of chronic pain (Thorn, 2004) and identifying and challenging unhelpful automatic thoughts and underlying core beliefs. |  | ● (1) |  |  | (1) via telephone |
| **7. Skills training and activity management + Education + Cognitive therapy methods** | | |  |  |  |  |  |
| Vitiello et al. [19] CBT-PI | Cognitive-behavioural pain coping skills intervention for osteoarthritis pain and insomnia. | Skills training and activity management  Relaxation, sleep enhancement techniques  Education  Pain and sleep management rationale  Cognitive therapy methods  Sleep and activity goal setting and pacing, sleep and pleasant activity scheduling and review, automatic thoughts and willingness problem-solving, maintenance plan. |  |  | ● |  |  |
| Vitiello et al. [19] CBT-P | Cognitive-behavioural intervention for osteoarthritis pain alone. | Skills training and activity management  Relaxation, guided imagery  Education  Pain and sleep management rationale  Cognitive therapy methods  Activity goal setting and pacing, pleasant activity scheduling, automatic thoughts and willingness, problem-solving, maintenance plan. |  |  | ● |  |  |
| **8. Education + Cognitive therapy methods + Methods to enhance acceptance, mindfulness and psychological flexibility + Graded activation guided by participant goals** | | | | | | |  |
| Yarns et al. [33] EAET | Emotional awareness and expression therapy. | Education  Psychoeducation on the therapy model (links pain, life stress, emotions, and brain changes).  Cognitive therapy methods  Identifying stress–symptom connections, attenuating pain through directly addressing stress and experiencing previously avoided emotions. Sharing and redefining shameful, secrets experiences.  Methods to enhance acceptance, mindfulness and psychological flexibility  Identifying stress–symptom connections, attenuating pain through directly addressing stress and experiencing previously avoided emotions. Sharing and redefining shameful, secrets experiences.  Graded activation guided by participant goals  Participant develops a written plan for continued exercises to meet own goals. | ● (1) |  | ● |  | (1) Orientation session |
| **MULTICOMPONENT PAIN MANAGEMENT PROGRAMMES** | | |  |  |  |  |  |
| **9. Physical exercise + Skills training and activity management** | | |  |  |  |  |  |
| Nicholas et al. [23] | An outpatient, CBT-based pain self-management programme that included exercises, pain education, and pain coping strategies. | Physical exercise  A combination of stretching, aerobic, and strengthening exercises; and functional tasks included repetitions of step-ups and walking.  Skills training and activity management  Speciﬁc skills taught included: setting speciﬁc, functional (and realistic) goals meaningful to the participant, activity pacing, arousal reduction using a combination of cognitive challenging and dispassionate exposure, dealing with ﬂare-ups, and structured problem-solving. |  | ● (1) | ● |  |  |
| Walsh et al. [32] | Facilitating Activity and Self-management in Arthritic Pain (FASA) - modified and derived from the ESCAPE-knee programme (Hurley et al., 2007) with amendments made to account for the involvement of multiple joints, in addition to usual primary care for chronic pain. | Physical exercise  A circuit of strengthening, aerobic and co-ordination exercises.  Skills training and activity management  Activity-rest cycling, pain relief and managing changes in pain, goal-setting and action plans, exercise recommendations and healthy eating. Action plan for physical activities to achieve every week with weekly progress review. |  |  | ● |  |  |
| **10. Physical exercise + Skills training and activity management + Graded activation guided by participant goals** | | | | | |  |  |
| Rini et al. [5] | PainCOACH program - an internet-based, pain coping skills training, non-pharmacological therapy, which provides interactive training in a cognitive or behavioural pain coping skill. | Physical exercise  Sitting and standing mini-practices  Skills training and activity management  Interactive training in a cognitive or behavioural pain coping skill, including concept of pain coping skills, activity/rest cycling, therapeutic rationale, relaxation techniques, goal setting, concept of negative automatic thoughts, concept of coping thoughts, monitoring, problem solving.  Graded activation guided by participant goals  Motivational interviewing to motivate initiation and adherence. | ● (1) | ● (2) |  |  | (1) Baseline only  (2) Via online website |
| Tse et al. [6] | Integrated motivational interviewing and physical exercise programme including pain education and pain controlling and coping skills. |  |  |  | ● |  | (1) A combination of strengthening and stretching exercises, and balancing and aerobic exercises to practise in face-to-face sessions and at home supplemented with a demonstrational booklet.  (2) Practise the pain management methods. Developing a learning contract for the pain management plan.  (3) Motivational interviewing to motivate initiation and adherence. |
| **11. Physical exercise + Skills training and activity management + Education** | | | | | |  |  |
| Costantino and Romiti [16] | Back School program (stretching and selective muscle reinforcement techniques) using manual treatments for relieving low back pain. | Physical exercise  Global bilateral exercises programme comprising slow and prolonged inhaling, includes holding in certain positions and repetitions - combination of stretching and muscular strengthening associated with proper breathing.  Skills training and activity management  Psychological aspects of pain and stress management.  Education  Anatomy of spinal column, its functioning and ergonomic position, and basis of the pain-inducing mechanism. | ? (uncertain) |  | ? (uncertain) |  |  |
| da Silva et al. [17] | A back school program which consists of educational lessons, physical exercises, and relaxation (massage) sessions. | Physical exercise  “Healthy back” physical exercises, including stretching, postural exercises, and strength training.  Skills training and activity management  Dealing with pain, ergonomics in daily living activities, and proper posture. Practise activities simulating real situation of daily living environment.  Education  Basic anatomy, spinal kinesiology and biomechanics, physiopathology of back | ? (uncertain) |  | ? (uncertain) |  | disorders |
| Jessep et al. [4] | Enabling Self-management and Coping with Arthritic knee Pain through Exercise (ESCAPE-knee pain) - integrated patient education (simple self-management and pain coping strategies) and individualised progressive exercise programme, with reduced number of sessions and a review session, in additional to usual primary care for chronic pain. | Physical exercise  Progressive exercise circuit to improve quadriceps strength, dynamic control, balance, co-ordination and function. At the end of intervention session, tailored home exercise regimen was written and provided to each participant.  Skills training and activity management  Goal setting and action planning for regular exercise, pacing an activity-rest cycle, medication review action plan, healthy eating, managing pain exacerbation, relaxation techniques. Review session to reinforce and review participant's home exercise regimen.  Education  Pain gate education | ● (1) |  | ● |  | (1) Review session 4 months after end of group sessions |
| Li et al. [10] | A dyadic pain management program, single-group (single dyad) program, composed of physical exercise and health education, delivered face-to-face and via digital tools. | Physical exercise  A combination of strengthening and stretching exercise, and balancing, flexibility, and transfer training in face-to-face sessions and for practice at home.  Skills training and activity management  Caring-related coping skills, the negative eﬀects of chronic pain, pharmacological and non-pharmacological (music therapy, deep breathing, aromatherapy, heat and cold) intervention pain management strategies, multisensory therapy, and method to reduce stress.  Education  Pain education (basic knowledge of pain, including physical and psychological effects, focuses on the theoretical level). | ● (1) |  | ● (2) |  | (1) To each dyad, Weeks 1-4  (2) Weeks 5-8, via written materials and digital tools |
| **12. Physical exercise + Skills training and activity management + Education + Cognitive therapy methods** | | | | | | |  |
| Cheng et al. [20] | A group intervention combining exercise and cognitive behavioural strategies. | Physical exercise  Stretching, strengthening, and balance exercises.  Skills training and activity management  Effectiveness of physical exercise on dealing with chronic pain, relaxation and breathing exercise, stress management, goal setting, assertive communication skills, self-compassion, activity scheduling, managing sleep, and positive self-statement.  Education  Understanding of pain, understanding of the stress-pain-appraisal connection, maintenance cycle relationships between thought, emotion, behaviour, and bodily sensation, importance of sleep.  Cognitive therapy methods  Focuses on participants’ response to pain, to motivate modifying dysfunctional thoughts and to explore realistic alternatives. | ● (1) |  | ● |  | (1) Home visits at 2 and 5 months after intervention |
| Goode et al. [7] | A home-based telephone-supported physical activity programme in conjunction with telephone-delivered cognitive-behavioural therapy for pain, among older adults with chronic low back pain. | Physical exercise  Tailored selection of flexibility, aerobic, and strengthening and stretching exercises given by written instructions and video.  Skills training and activity management  Activity pacing, distraction, breathing relaxation, progressive muscle relaxation  Education  Introduction to chronic low back pain, function, and gate theory of pain, information for exercise or healthy eating.  Cognitive therapy methods  Overcoming pain-related barriers, managing pain associated with activity, cognitive restructuring involves identifying and reframing negative thought patterns, behaviours, and emotional responses relating to pain. |  | ● (1) |  |  | (1) Via telephone, written information, video |
| **13. Physical exercise + Encouraging physical activity + Skills training and activity management** | | | | | | |  |
| Bearne et al. [2] | Exercise‐based exercise and self‐management rehabilitation Programme for chronic hip pain, in addition to usual management by general practitioner. | Physical exercise  Combination of resistance, aerobic, functional and balance/coordination exercises.  Encouraging physical activity  Home exercise  Skills training and activity management  Coping strategies, self-care, pain control, joint protection, problem-solving, weight management, regular physical activities |  | ● (1) | ● |  | (1) (instructions for home exercise) |
| **14. Physical exercise + Encouraging physical activity + Skills training and activity management + Education** | | | | | | |  |
| Hurley et al. [3] | Enabling Self-management and Coping with Arthritic knee Pain through Exercise (ESCAPE-knee pain) - integrated patient education (simple self-management and pain coping strategies) and individualised progressive exercise programme, in additional to usual primary care for chronic pain. | Physical exercise  Combination of resistance, aerobic, functional and balance/coordination exercises; tailored to the participant's ability, progression rate, and identified areas of disability, the exercise specificity was varied and the complexity and intensity was increased gradually.  Encouraging physical activity  At the end of intervention session, specific advice and written instructions for simple home exercise programme was provided to each participant.  Skills training and activity management  Arthritis self-care advice - regular exercise, pain control, joint protection, medication, relaxation techniques, problem solving and planning to promote and adopt lifestyle changes for joint health.  Education  Pain perceptions and biopsychosocial model of pain. | ● (1) |  | ● (2) |  | (1) 146 participants in Individual-rehab arm  (2) 132 participants in Group-rehab arm |
| **15. Physical exercise + Encouraging physical activity + Skills training and activity management + Education + Cognitive therapy methods + Methods to enhance acceptance, mindfulness and psychological flexibility** | | | | | | |  |
| Weiner et al. [15] | A comprehensive structured evaluation and algorithm-guided treatment in Aging Back Clinics care, which includes physical examination to identify pain contributors, participant education and collaborative decision-making. | Physical exercise  (Available to be suggested by algorithm and agreed between clinician and participant) Physical therapy, aquatherapy, yoga, Tai Chi  Encouraging physical activity  (Available to be suggested by algorithm and agreed between clinician and participant)  Skills training and activity management  Devising treatment plan. (Available to be suggested by algorithm and agreed between clinician and participant) Sleep clinic  Education  Education about each individual's CLBP contributors, evaluation findings summary. (Available to be suggested by algorithm and agreed between clinician and participant) Sleep hygiene education  Cognitive therapy methods  (Available to be suggested by algorithm and agreed between clinician and participant) Cognitive behavioural therapy.  Methods to enhance acceptance, mindfulness and psychological flexibility  (Available to be suggested by algorithm and agreed between clinician and participant) Maintain spiritual support for coping, psychology referral, Mindfulness Online & Virtual Exercise for Chronic Pain (MOVE programme). | ● (1) |  |  |  | (1) (For evaluation, examination, devising treatment plan) |
| **16. Encouraging physical activity + Skills training and activity management + Graded activation guided by participant goals** | | | | | | |  |
| Ersek et al. [13] | Chronic pain self-management training group intervention for older adults. | Encouraging physical activity  Demonstrate and practise specific exercises. Discuss exercise in pain management, including types of exercise, tips for starting exercise program.  Skills training and activity management  Pain education, relaxation training and practice, physical exercise/activity, pacing activities, challenging negative thoughts, dealing with setbacks, pharmacological and non-pharmacological therapies.  Graded activation guided by participant goals  Develop individualised pain management goals: identify needs and goals, goal-setting, identify and problem-solve for obstacles, monitor and review progress. |  |  | ● |  |  |
| **17. Encouraging physical activity + Skills training and activity management + Education** | | | | | | |  |
| Kwok et al. [26] | A self-management program on pain, which adopted the constructions of evidence-based Arthritis Self-management Programme, conducted in a mobile setting. | Encouraging physical activity  Activity of daily living training, physical exercise (including stretching, strengthening, and aerobic exercises)  Skills training and activity management  Goal-setting, action planning, self-monitoring with reviews; practice on skills about problem solving, cognitive symptom management, communication with family and health professionals, social activities.  Education  Information on diseases, the five core self-management skills, different forms of counselling and therapy, social support, healthy eating. |  |  | ● |  |  |
| **18. Encouraging physical activity + Skills training and activity management + Education + Methods to enhance acceptance, mindfulness and psychological flexibility** | | | | | | |  |
| Fanning et al. [14] | MORPH intervention is a weight loss and sedentary behaviour telecoaching intervention, comprises of group telecoaching sessions, educational videos, and a package of mHealth tools. | Encouraging physical activity  Promote increase in physical activity levels and reducing sedentary behaviour  Skills training and activity management  Using mobile app and linked eHealth tools (Fitbit, scale) - to set goals for and monitor weight, dietary intake, physical activity level.  Education  Dietary, behavioural change, increasing physical activity, pain management, mindfulness-based relapse prevention (in group sessions)  Methods to enhance acceptance, mindfulness and psychological flexibility Practise brief mindfulness-based exercises to improve affect, reduce pain, and prevent overeating or extended sitting (in group sessions) | ● (1) | ● (2) | ● (3) | ● (4) | (1) Orientation only  (2) Via mobile phone app  (3) Weeks 1-3)  (4) (Weeks 4-12, via mobile phone app |
| **19. Encouraging physical activity + Skills training and activity management + Education + Graded activation guided by participant goals** | | | | | | |  |
| Janevic et al. [9] | “Positive STEPS” which blends positive activities with core chronic pain self-management skills and is tailored for cultural relevance to African American older adults. | Encouraging physical activity  Participants are encouraged to become active and using the activity tracker to monitor their activity level.  Skills training and activity management  Core pain self-management skills: Understanding Chronic Pain, Staying Active, and Doing What You Love.  Education  Information about recreation activities and resources in the neighbourhood.  Graded activation guided by participant goals  SMART Goal-setting with review and discussion about barriers. |  | ● (1) |  |  | (1) Via mailed materials, telephone, text messages, websites, and videos |
| CBT=cognitive behaviour therapy… | | | | | | | |

**Table 7. Participant engagement**

|  | **Expected input (what the participants are expected to do)** | **Use of intervention (e.g., attendance, reported compliance)** | **Retention (e.g., dropouts from intervention)** | **Satisfaction / comments about intervention (e.g., rating about satisfaction, usefulness of intervention)** |
| --- | --- | --- | --- | --- |
| **Andersson et al. [28]** | To attend weekly group session for 6 weeks, participate in the exercises during the sessions, and to complete homework assignments from each session. | Not reported. | Appeared no dropouts from intervention. | Not reported. |
| **Baird and Sands [29]** | To listen to the provided 10-to-15-minute audiotaped guided imagery script twice a day.   To complete a journal 3 times a week with information about symptoms and the number of times a day they used guided imagery. | Journal recordings revealed consistent use and compliance with the Intervention, % of participants: 88.8% Used guided imagery once a day 48.8% Used twice a day 10.7% Used 3-4 times a day | Dropped out of the study after the initial questionnaire before starting the intervention*: 12.5% (n=1/18) Reason not reported | 23.5% (n=4/17) Difficult at time scheduling intervention in their busy lives 11.8% (n=2/17) Occasionally fell asleep before completing the guided imagery when used at bedtime 11.8% (n=2/17) Had difﬁculty concentrating when in severe pain |
| **Bearne et al. [2]** | To complete supervised exercise circuit and participate in discussion in group session, twice a week for 5 weeks.  To perform a simple home exercise programme. | 81% Mean attendance at the rehabilitation sessions | Withdrew during rehabilitation*: 4.2% (n=1/24) Failed to begin rehabilitation 4.2% (n=1/24) Withdrew due to other commitments | Participants' informal feedback showed that the programme’s premise, content and delivery was understood, popular and well tolerated. |
| **Berman et al. [11]** | To use the online intervention at least once a week for 6 weeks: to access the website on own schedule, use the intervention at own pace, at a location of own choice.  To use the alternative modes of learning: online materials included audio, visual, and textual components included illustrative examples and worksheets to reﬂect on responses to pain and develop a plan of action. | Number of visits to the website over 6-week intervention period (n=41): median 22.5 visits At log off, 78% of the time participants indicated that they planned to use 1 or more exercises on their own while ofﬂine  Use of intervention (n=43): 95.3% used the self-care techniques learned from the intervention while offline Participants reported using techniques from all of the modules, most commonly mentioned: 74.4% Relaxation 69.8% Abdominal breathing techniques | Discontinued participation in the study*, reported reasons included: 7.3% (n=3/41) Personal health and/or family problems 2.4% (n=1/41) Difﬁculties with using a computer and the website 2.4% (n=1/41) Physically uncomfortable sitting at a computer 12.2% (n=5/41) No response to contact | Follow-up survey (n=43): 81.4% Believed intervention was helpful or very helpful 88.4% Found the website easy or very easy to navigate 95.3% Would recommend the intervention to others  All modules were found to be helpful or very helpful by a majority of participants who used them. Particularly useful were: 90.5% Relaxation module 85.4% Abdominal breathing 78.3% Appreciation and pleasure 70.0% Appreciative writing 66.7% Planning for change 62.1% Visual expression 57.5% Writing about difﬁcult experiences 95.1% Examples or illustrations 75% Response provided 81.3% Action plan worksheets Participants commented on the user-friendly nature and convenience of the website and the value of access to comprehensive, positive-tone, clear explanations and self-care techniques, which helped their focusing on pain experience and self-care.  Negative comments about intervention content:  4.7% (n=2/43) Disliked the optional self-reflection worksheets 2.3% (n=1/43) Disliked some of the graphics and voices in the audio modules 4.7% (n=2/43) Did not believe “they got much out of” the intervention 2.3% (n=1/43) Were bored and thought that it was “time-consuming” A few participants were initially sceptical about the mind-body techniques beneﬁts but eventually found them helpful. |
| **Broderick et al. [25]** | To practise the skills at home, and record the home practice. | 80.6% (n=104/129) Completed all 10 sessions 17.8% (n=23/129) Completed <10 sessions 1.6% (n=2/129) Did not schedule any session | Attrition at post-treatment (n=129)*: 12% Reasons not reported | Skills most helpful and practised most across time: Progressive muscular relaxation, mini practices of relaxation, and activity pacing.  Skills found most valuable: Pleasant imagery and distraction (rated most valuable by approximately 40% participants) |
| **Carmody et al. [30]** | To attend 12 telephone sessions over 20 weeks.  To complete task assignment from each session.  As the therapy progressed, to increase role in setting the session agenda and tailor homework. | 9.5 sessions on average attended by participants 66% (n=33/50) Attended ≥10 sessions | 32% (16/50) Loss to follow-up before end of intervention* | Helpfulness of intervention: Overall helpfulness rating (10-point scale): Mean 7.54 (SD 2.13) Participants rated intervention components helpful: 82.7% (range 69.2-94.1% across items) Participants indicated that they made use of specific intervention components/topics: 95.5% (range 87.9-100% across items) |
| **Cheng et al. [20]** | To participate in physical exercises and group discussion in the sessions.  To complete homework about pain diary (mood monitor), pain and stress relationship worksheet, thought record sheet, self-statement record sheet, mood thermometer worksheet, assertiveness worksheet, activity diary, keeping a log of thankful events and grounding exercise, and goal setting worksheet. | Attendance by participants (n=77): mean 83.8% (SD 1.14) | Discontinued intervention (n=12/89): 2.2% (n=2/89) Lack of interest 7.9% (n=7/89) Busy 3.4% (n=3/89) Reasons not reported | Not reported. |
| **Costantino and Romiti [16]** | To attend and participate in 2 sessions per week for 12 weeks. | Study authors stated a high participant compliance. | Dropout*: 3.6% (n=1/28) Did not receive entire intervention due to family problems | Not reported. |
| **da Silva et al. [17]** | To attend 10 sessions over 5 weeks.  To use the pamphlets to exercise at home. | Not reported. | Dropouts*: 27% (n=3/11) Health or personal reasons | Not reported. |
| **Ersek et al. [12]** | To attend weekly group session every week for 6 weeks and final session at Week 8.  To participate in group discussion and develop a specific plan for working towards activity goals.  To practise pain management skills regularly. | Attendance: 77.3% (n=17/22) Attended ≥80% (6/7) of the sessions 4.5% (n=1/22) Attended 2 sessions 4.5% (n=1/22) attended 4 sessions 4.5% (n=1/22) Attended 5 sessions 9.1% (n=2/22) Never attend any classes or provide a reason for dropping out of the study  Use of intervention: Overall mean rating for all syllabus topics (0=“I did not look at the section at all” to 5=“I read the section thoroughly”): 4.4 (SD 0.5, range=0-5)  Percentage of participants (n=19) using the strategy at least once weekly: 58% Strengthening/balance exercises 42% Stretching exercises 89% Aerobic exercise 42% Applications of heat  16% Applications of cold  74% Relaxation exercises  58% Countering negative thoughts about the pain  79% Engagement in pleasant activities  74% Pain medications  5% Complementary therapies (e.g., herbal remedies, chiropractic care, acupuncture) | Dropouts before 3-month follow-up*: 5% (n=1/22) Increased pain 10% (n=2/22) Reasons unknown | Overall usefulness of treatment rating (0=“not at all useful” to 5=“very useful”): Mean 4.3 (SD 0.5)  Perceived usefulness of the strategy (0–5 scale): Strengthening/balance exercises: 2.3 Stretching exercises: 2.1 Aerobic exercise: 3.7 Applications of heat: 2.4 Applications of cold: 1.6 Relaxation exercises: 3.9 Countering negative thoughts about the pain: 2.9 Engagement in pleasant activities: 3.4 Pain medications: 3.6 Complementary therapies (e.g., herbal remedies, chiropractic care, acupuncture): 0.4 |
| **Ersek et al. [13]** | To attend weekly group sessions for 7 weeks.  To develop a personalised pain management plan, practise pain management skills, and record progress. | Attendance (n=133): 82.7% (n=110) Attended ≥5 sessions 5.3% (n=7) Attended ≤1 session | Retention in study at end of intervention for follow-up*: 92.5% (n=123/133) | Not reported. |
| **Fanning et al. [14]** | To attend a pre-study orientation, face-to-face weekly group sessions for 3 weeks, followed by weekly group video-conference for 9 weeks.  To wear the Fitbit activity monitor, weigh, and maintain dietary logs, daily.  Using the intervention smartphone app, to monitor movement and receive feedback with the intervention smartphone app at least once daily and frequently throughout the day.  To view the intervention videos and listen to podcasts on the app. | Average number of accesses to intervention app per week: Highest in the second week: 18.40 times Lowest in the ninth week: 6.47 times Generally remaining ≥1 daily accesses throughout intervention | Retention in study at end of intervention for follow-up*: 80% (n=12/15) | Not reported. |
| **Goode et al. [7]** | To attend 13 telephone sessions over 12 weeks.  To follow the personalised exercise programme and recommendations, written instructions and videos to exercise.  To practise progressive muscle relaxation. | Among all participants who initiated the Physical activity + cognitive-behavioural therapy for pain programme, average number of intervention calls completed: 8.8 (SD 2.37)  Among participants who did not withdraw before the end of intervention, average number of intervention calls completed: 10.4 | Withdrawals: 20% (n=4/20) Reasons not reported | Not reported. |
| **Haas et al. [18]** | To attend group workshop sessions for 6 weeks, participate in group interaction and team tasks, and develop an individual action plan. | Attendance (n=60): 16% Attended all classes 68% Attended ≥3 classes.  The reasons for poor attendance (19 persons with <3 classes): 5% (n=3/60) Reported comorbid complaints including severe hip pain and hospitalization with a heart problem 3.3% (n=2/60) Did not like the class 1.7% (n=1/60) Refused to attend 8.3% (n=5/60) had no reason recorded 13.3% (n=8/60) Could/would not be contacted | Not reported. | Not reported. |
| **Hasegawa et al. [31]** | To attend and participate in 24 sessions over 12 weeks. | Not reported. | Not reported for the first phase before washout period. | Not reported. |
| **Hausmann et al. [8]** | To attend 6 telephone sessions weekly for 6 weeks.  To complete weekly at-home activities. | Adherence: 76.2% (n=16/21) Completed ≥5 of 6 weekly calls 61.9% (n=13/21) Completed ≥5 of 6 weekly activities | Dropouts*: 9.5% (n=2/21) Withdrew after baseline because the program did not match their expectations | Ratings of completed weekly activities (mean, 1=not at all; 7=extremely): Perceived benefit: 5.2 Perceived enjoyment: 5.4 Perceived difficulty: 1.8 |
| **Hurley et al. [3]** | To attend 12 session for 6 weeks (in group if in Group-rehabilitation arm).  To participate in discussion and physical exercise, and practise relaxation techniques.  To practise the home exercise regimen.  To develop a personalised action plan and review progress. | Of the participants followed up at 6 months: 85% (n=105/120) in individual-rehabilitation arm and 55% (n=59/107) in group-rehabilitation arm attended ≥10 of the 12 sessions | Lost to follow-up at end of intervention*: 11.0% (n=16/146) individual-rehabilitation arm 14.4% (n=19/132) group-rehabilitation arm | Not reported. |
| **Janevic et al. [9]** | To attend weekly telephone session, wear an activity tracker during waking hours and report step count daily, for 7 weeks.  To complete the program tasks including watching videos. | Number of participants completed each session: 92% (n=23/25) Session 1 and 7 88% (n=22/25) Session 2-4 84% (n=21/25) Session 5-6  Mean 5.7 of 7 telephone sessions completed 95.2% (n=20/21) Watched all programme videos | Dropouts*: 8% (n=2/25) Withdrew 4% (n=1/25) Lost to follow-up | Satisfaction (n=21): Participating in the programme increased my understanding of pain management: 61.9% Strongly agree 33.3% Agree 4.8% Neither agree nor disagree  Participating in the programme helped me reach my pain management goals: 47.6% Strongly agree 52.4% Agree  The community health worker explained the information in a way that was easy to understand: 85.7% Strongly agree 14.3% Agree  The information and materials in the workbook helped me better understand how to manage my chronic pain: 71.4% Strongly agree 28.6% Agree |
| **Jessep et al. [4]** | To attend 10 group sessions over 5 weeks and 1 review session 4 months after the end of intervention; to participate in exercise and group discussion; to practise relaxation techniques.  To continue the exercise with a tailored home exercise regimen. | 83% (24/29) Attended ≥8 sessions 13.8% (n=4/29) Attended 6-7 sessions 3.4% (n=1/29) Attended 4 sessions 89% (n=26/29) Attended the review session | Lost to follow-up at 6 weeks*: 3.4% (n=1/29) Withdrew 3.4% (n=1/29) Developed health problems 3.4% (n=1/29) Diagnosed with Polymyalgia Rheumatica | Not reported. |
| **Kwok et al. [26]** | To attend weekly group sessions for 6 weeks, participate in discussion and practice of skills.  To generate a short-term action plan and self-monitor and evaluate progress. | Not reported. | No dropouts during study period. | Not reported. |
| **Li et al. [10]** | To attend weekly sessions with an informal caregiver for 4 weeks, and continue the home-based programme for the following 4 weeks, to participate in physical exercises and practice of skills  To use messaging tools to communicate with study team and support performing intervention. | Number of WhatsApp messages (including video and voice), average per participant: Total number of WhatsApp messages: 127.1 Sent by researchers: 88.1  Sent by participants: 38.9 | Dropouts: 15.6% (n=5/32) dyads due to tight schedule, previous engagement, or could not be contacted | Most of the participants reported that they were satisﬁed with this DPMP program, and felt that it was worth spending time on.  Participants showed a willingness to recommend this program to others.  Particular themes of comments and feedback from participants: Perceived benefits: helping both older adults and their informal caregivers. Communication: opportunities to communicate with peers and the elderly. Boosted sense of self-worth. Feedback on the content: like the programme; to extend exercise time, more interactive games, alternate a face-to-face program with a digital education programme, reduce the complex introduction of professional knowledge and use more pictures; messages can remind me do the exercises, videos are useful for exercises. |
| **Morone et al. [21]** | To attend weekly group sessions for 8 weeks, to partake in meditation and discussion.  To meditate daily (6 days/week) for 45 minutes, to record in a diary.  To continue to meditate after the end of intervention period. | Average number of classes attended: 6.7 (range 5–8) Average number of days per week meditated: 4.3 (range 0–7) Average minutes per day meditated: 31.6 (range 0–52) | 68% (n=13/19) Completed intervention programme  Declined intervention: 10.5% (n=2/19) Too busy 5.3% (n= 1/19) Scheduling conflict 15.8% (n=3/19) Unexpected health or family obligations | Not reported. |
| **Morone et al. [22]** | To attend weekly group sessions for 8 weeks, to partake in meditation and discussion.  To meditate daily (6 days/week) for 45 minutes, to record in a diary. | Number of classes attended: average 7.5 (range 6-8) Meditation practice: average 5 days a week (range 1-7), average 31 minutes/session (range 22-48)  Practice of meditation four months after programme completion: 87.5% (n=14/16) Continued to meditate, in a week: n=1/14 once, 7/14 twice, 3/14 3-5 times, 3/14 daily 87.5% (n=14/16) Practised formal meditation 94% (15/16) Practised informal meditation, in a week: 1/15 once, 4/15 twice, 6/15 3-5 times, 4/15 daily Recommended the class to others: 13/16 Yes, 3/16 No Can concentrate better after learning mindfulness meditation: 87.5% (n=14/16) Yes, 6.3% (n=1/16) No, 6.3% (n=1/16) No answer | Overall completion rate: 80% (16/20) 10% (n=2/20) Did not attend any classes because the class was too early 5% (n=1/20) Dropped out after 1 class, no longer interested 5% (n=1/20) Dropped out after 3 classes, no longer interested | Not reported. |
| **Morone et al. [24]** | To attend weekly group sessions for 8 weeks, to partake in meditation and discussion.  To meditate daily (6 days/week) for 45 minutes, to record in a diary.  To return for 6-monthly booster sessions. | Attendance, mean: 6.6 Group sessions (range 0-8) 2.4 Booster sessions | Intervention was not delivered to: 1.4% (n=2/140) No longer interested in participating in the study 0.7% (n=1/140) Developed a serious illness 0.7% (n=1/140) Unexpected family obligations | Not reported. |
| **Nicholas et al. [23]** | To attend 8 group sessions over 4 weeks.  To perform the exercises and skills during the sessions and at home, and read the Manage Your Pain book as the manual.   To self-monitor and record attempts to practice the exercises and to apply the skills at home. | Not reported. | Completed follow-up at end of intervention*: 88% (n=43/49) Completed 12% (n=6/49) Not completed | Treatment credibility and satisfaction (mean, out of 10): Credibility: 5.62 (SD 1.83) Satisfaction: 9.24 (1.18)  “Whether or not they would recommend this treatment for a friend or relative who had a similar pain problem?”: 89% Yes |
| **Rini et al. [5]** | To complete 8 online modules in a self-directed manner in 8 weeks, and practise the skills and training.  To practise regularly, set up practice reminders, practice goals, review progress. | Completed all 8 modules: 91% (n=53/58) Completed some modules: 3.4% (n=2/58) Did not complete any module: 5.2% (n=3/58) Prompts to log in (n=58): 43.1% no prompt needed, 34.5% needed one prompt, 12.1% needed two prompts, 10.3% needed three or more prompts.  Use of resources in PainCOACH (online intervention) (n=53):  Used the workbook to take notes: 85.5% Completed worksheets: 79.6% Read other people’s stories in COACHchat: 81.8% Shared own stories: 25.5% Used COACHtrack to log practices: 54.5% Viewed own progress in practices and/or self-efficacy: 65.5% Changed own practice goals: 68.5% Managed automated practice reminders: 56.4% Recorded own self-efficacy: 51.9% Preference for completing PCST on a computer, baseline to post-intervention: increased from 50% to 62% | Not completed follow-up assessments*: 3.4% (n=2/58) At midpoint 1.7% (n=1/58) At end of intervention | Not reported. |
| **Tse et al. [6]** | To attend weekly group sessions for 8 weeks, to practise the physical exercises and self-management skills and participate in motivational interviewing discussion.  To develop a ‘pain contract’ (pain self-management plan).  To practise the exercises at home ≥4 times per week. | Overall average attendance: 81.7% per participant  Compliance with home exercise practice, i.e., ≥4 times per week (120 minutes in total) during 8 weeks intervention: 84.3% participants achieved | Lost to follow-up*: 3.2% (n=1/31) Could not be contacted | Not reported. |
| **Vitiello et al. [19] CBT-PI** | To attend weekly group sessions for 6 weeks.  To practise skills and techniques, set goals, and develop maintenance plan in sessions.  To complete homework. | Attendance (n=122): 93% Attended ≥4 of 6 sessions | Withdrew from study*: 8.2% (n=10/122) Withdrawals 0.8% (n=1/122) Died | Not reported. |
| **Vitiello et al. [19] CBT-P** | To attend weekly group sessions for 6 weeks.  To practise skills and techniques, set goals, and develop maintenance plan in sessions.  To complete homework. | Attendance (n=122): 92% Attended ≥4 of 6 sessions | Withdrew from study*: 4.9% (n=6/122) Withdrawals 1.6% (n=2/122) Moved out of area 1.6% (n=2/122) Died | Not reported. |
| **Walsh et al. [32]** | To attend 12 group session over 6 weeks.  To participate in discussion and physical exercise, and practise relaxation techniques.  To practise home exercise regimen.  To develop a personalised action plan and review progress. | Average number of sessions attended: 8 sessions 83% (n=137/166) participants attended ≥50% scheduled sessions | Lost to follow-up, at end of intervention*: 1.2% (n=2/170) Time commitments 1.8% (n=3/170) Unrelated illness 1.2% (n=2/170) Family illness 8.8% (n=15/170) No contact | Not reported. |
| **Weiner et al. [15]** | At baseline, to have a structured history and physical examination to identify pain contributors; to receive structured participant education; with the clinician and guided by condition-specific algorithms, collaboratively to devise a treatment plan.  To follow/receive recommended and agreed treatments, with the freedom to refuse any treatment. | 24% (n=6/25) participants refused/declined some of the recommended treatment. Participants' conditions/symptoms and recommended treatment(s) refused/declined: • Myofascial pain and maladaptive coping: CBT (participant declined) • Insomnia and myofascial pain: Sleep clinic (participant declined) • Myofascial pain, iliotibial band pain, and insomnia: Sleep clinic (participant declined) • Sacroiliac joint pain: discontinue methocarbamol (participant refused) • Myofascial pain, hip osteoarthritis, and insomnia: MOVE** referral (participant refused) • Myofascial pain and hip osteoarthritis: MOVE** referral (participant refused), reduce cyclobenzaprine dose (participant refused discontinuation) dose (participant refused discontinuation) | Lost to follow-up*: 4% (n=1/25) Reason not reported | Qualitative feedback from interviews with participants (n=23): Perceived most helpful treatments: the combination of all the treatments received, as well as physical therapy and independent exercises, acupuncture, back injections, transcutaneous electrical nerve stimulation (TENS), and medications. Perceived least helpful treatment: physical therapy, independent exercises, acupuncture, and epidural. Most participants said that all aspects of their pain and functioning had been adequately addressed since the initial visit with the study clinicians at baseline. A few participants commented that their upper leg or iliotibial (IT) band pain, knee pain, and spinal stenosis were not adequately addressed. Suggestions for improving the Department of Veterans Affairs (VA) management of chronic low back pain: more check-ins or follow-ups with more consistent/frequent treatment, listening to the patient, treatment closer to the patient’s home, consulting with private doctors, explaining management against eliminating pain, not pushing for surgery, more communication between departments, staying up-to-date on research, stopping prescription of pain medications, VA gym or gym access, and continuing programmes and research for pain. |
| **Yarns et al. [33] EAET** | To attend 1 individual session, followed by 8 group sessions for 8 weeks.  To practise skills in sessions.  To complete home exercises, include reading and completing worksheets, throughout therapy.  To develop a written plan for continued exercises to meet their goal. | Number of group sessions attended (out of 8), mean: 5.6 (SD 2.3) | Withdrawals*: 3.6% (n=1/28) Scheduling issues, between individual and beginning of group sessions 10.7% (n=3/28) Lost contact/no reply during group sessions | Satisfaction with therapist, 6 items (range 6 worst - 30 best), mean: 26.6 (SD 4.2) Satisfaction with therapy, 6 items (range 6 worst - 30 best), mean: 25.7 (SD 5.0) Global satisfaction (range 1 lowest -5 highest), mean: 1.8 (SD 0.7) |
| **Yarns et al. [33] CBT** | To attend 1 individual session, followed by 8 group sessions for 8 weeks.  To practise skills in sessions.  To review plan and plan for future. | Number of group sessions attended (out of 8), mean: 6.2 (2.0) | Withdrawals*: 4% (n=1/25) Lack interest, between individual and beginning of group sessions 4% (n=1/25) Lost contact/no reply during group sessions | Satisfaction with therapist, 6 items (range 6 worst - 30 best), mean: 26.7 (SD 3.2) Satisfaction with therapy, 6 items (range 6 worst - 30 best), mean: 25.5 (SD 3.8) Global satisfaction (range 1 lowest -5 highest), mean: 1.6 (SD 0.6) |
| SD=standard deviation; CBT-PI= cognitive-behavioural therapy for pain and insomnia; CBT-I=cognitive-behavioural therapy for insomnia; EAET= emotional awareness and expression therapy; CBT= cognitive behavioural therapy  *=information about retention or dropouts from the study, not specifically about retention in the intervention; **MOVE! is a weight management, health promotion programme designed to improve the lives of veterans. | | | | |

# References

[1] Tong A, Flemming K, McInnes E, Oliver S, Craig J. Enhancing transparency in reporting the synthesis of qualitative research: ENTREQ. *BMC Med Res Methodol* 2012;**12**(1):181. https://doi.org/10.1186/1471-2288-12-181

[2] Bearne LM, Walsh NE, Jessep S, Hurley MV. Feasibility of an exercise-based rehabilitation programme for chronic hip pain. *Musculoskelet Care* 2011;**9**(3):160-8. https://doi.org/10.1002/msc.209

[3] Hurley MV, Walsh NE, Mitchell HL, Pimm TJ, Patel A, Williamson E*, et al.* Clinical effectiveness of a rehabilitation program integrating exercise, self‐management, and active coping strategies for chronic knee pain: A cluster randomized trial. *Arthritis Rheum* 2007;**57**(7):1211-9. https://doi.org/10.1002/art.22995

[4] Jessep SA, Walsh NE, Ratcliffe J, Hurley MV. Long-term clinical benefits and costs of an integrated rehabilitation programme compared with outpatient physiotherapy for chronic knee pain. *Physiotherapy* 2009;**95**(2):94-102. https://doi.org/10.1016/j.physio.2009.01.005

[5] Rini C, Porter LS, Somers TJ, McKee DC, DeVellis RF, Smith M*, et al.* Automated Internet-based pain coping skills training to manage osteoarthritis pain: A randomized controlled trial. *Pain* 2015;**156**(5):837-48. https://doi.org/10.1097/j.pain.0000000000000121

[6] Tse MMY, Vong SKS, Tang SK. Motivational interviewing and exercise programme for community-dwelling older persons with chronic pain: a randomised controlled study. *J Clin Nurs* 2013;**22**(13-14):1843-56. https://doi.org/10.1111/j.1365-2702.2012.04317.x

[7] Goode AP, Taylor SS, Hastings SN, Stanwyck C, Coffman CJ, Allen KD. Effects of a home-based telephone- Supported physical activity program for older adult veterans with chronic low back pain. *Phys Ther* 2018;**98**(5):369-80. https://doi.org/10.1093/ptj/pzy026

[8] Hausmann LRM, Youk A, Kwoh CK, Ibrahim SA, Hannon MJ, Weiner DK*, et al.* Testing a positive psychological intervention for osteoarthritis. *Pain Med* 2017;**18**(10):1908-20. https://doi.org/10.1093/pm/pnx141

[9] Janevic M, Robinson-Lane SG, Courser R, Brines E, Hassett AL. A community health worker-led positive psychology intervention for African American older adults with chronic pain. *Gerontologist* 2022;**62**(9):1369–80. https://doi.org/10.1093/geront/gnac010

[10] Li Z, Tse M, Tang A. The effectiveness of a dyadic pain management program for community-dwelling older adults with chronic pain: A pilot randomized controlled trial. *Int J Environ Res Public Health* 2020;**17**(14):09. https://doi.org/10.3390/ijerph17144966

[11] Berman RLH, Iris MA, Bode R, Drengenberg C. The Effectiveness of an Online Mind-Body Intervention for Older Adults With Chronic Pain. *J Pain* 2009;**10**(1):68-79. https://doi.org/10.1016/j.jpain.2008.07.006

[12] Ersek M, Turner JA, McCurry SM, Gibbons L, Kraybill BM. Efficacy of a self-management group intervention for elderly persons with chronic pain. *Clin J Pain* 2003;**19**(3):156-67. https://doi.org/10.1097/00002508-200305000-00003

[13] Ersek M, Turner JA, Cain KC, Kemp CA. Results of a randomized controlled trial to examine the efficacy of a chronic pain self-management group for older adults [ISRCTN11899548]. *Pain* 2008;**138**(1):29-40. https://doi.org/10.1016/j.pain.2007.11.003

[14] Fanning J, Brooks AK, Ip E, Nicklas BJ, Rejeski JW, Nesbit B*, et al.* A mobile health behavior intervention to reduce pain and improve health in older adults with obesity and chronic pain: The MORPH pilot trial. *Front Digit Health* 2020;**2**. https://doi.org/10.3389/fdgth.2020.598456

[15] Weiner DK, Gentili A, Rossi M, Coffey-Vega K, Rodriguez KL, Hruska KL*, et al.* Aging back clinics-a geriatric syndrome approach to treating chronic low back pain in older adults: Results of a preliminary randomized controlled trial. *Pain Med* 2020;**21**(2):274-90. https://doi.org/10.1093/pm/pnz179

[16] Costantino C, Romiti D. Effectiveness of Back School program versus hydrotherapy in elderly patients with chronic non-specific low back pain: a randomized clinical trial. *Acta Biomed* 2014;**85**(3):52-61.

[17] da Silva TMJC, da Silva NN, de Souza Rocha SH, Déborah Marques de O, Monte-Silva KK, Angélica da Silva T*, et al.* Back school program for back pain: education or physical exercise? *Conscientiae Saúde* 2014;**13**(4):506-15. https://doi.org/10.5585/conssaude.v13n4.5191

[18] Haas M, Groupp E, Muench J, Kraemer D, Brummel-Smith K, Sharma R*, et al.* Chronic disease self-management program for low back pain in the elderly. *J Manipulative Physiol Ther* 2005;**28**(4):228-37. https://doi.org/10.1016/j.jmpt.2005.03.010

[19] Vitiello MV, McCurry SM, Shortreed SM, Balderson BH, Baker LD, Keefe FJ*, et al.* Cognitive-behavioral treatment for comorbid insomnia and osteoarthritis pain in primary care: The Lifestyles randomized controlled trial. *J Am Geriatr Soc* 2013;**61**(6):947-56. https://doi.org/10.1111/jgs.12275

[20] Cheng ST, Chen PP, Chow YF, Law ACB, Lee JSW, Leung EMF*, et al.* An exercise cum cognitive-behavioral intervention for older adults with chronic pain: A cluster-randomized controlled trial. *J Consult Clin Psychol* 2022;**90**(3):221-33. https://doi.org/10.1037/ccp0000698

[21] Morone NE, Greco CM, Weiner DK. Mindfulness meditation for the treatment of chronic low back pain in older adults: A randomized controlled pilot study. *Pain* 2008;**134**(3):310-9. https://doi.org/10.1016/j.pain.2007.04.038

[22] Morone NE, Rollman BL, Moore CG, Li Q, Weiner DK. A mind–body program for older adults with chronic low back pain: Results of a pilot study. *Pain Med* 2009;**10**(8):1395-407. https://doi.org/10.1111/j.1526-4637.2009.00746.x

[23] Nicholas MK, Asghari A, Blyth FM, Wood BM, Murray R, McCabe R*, et al.* Self-management intervention for chronic pain in older adults: A randomised controlled trial. *Pain* 2013;**154**(6):824-35. https://doi.org/10.1016/j.pain.2013.02.009

[24] Morone NE, Greco CM, Moore CG, Rollman BL, Lane B, Morrow LA*, et al.* A mind-body program for older adults with chronic low back pain: A randomized clinical trial. *JAMA Intern Med* 2016;**176**(3):329-37. https://doi.org/10.1001/jamainternmed.2015.8033

[25] Broderick JE, Keefe FJ, Bruckenthal P, Junghaenel DU, Schneider S, Schwartz JE*, et al.* Nurse practitioners can effectively deliver pain coping skills training to osteoarthritis patients with chronic pain: A randomized, controlled trial. *Pain* 2014;**155**(9):1743-54. https://doi.org/10.1016/j.pain.2014.05.024

[26] Kwok EYT, Au RKC, Li-Tsang CWP. The effect of a self-management program on the quality-of-life of community-dwelling older adults with chronic musculoskeletal knee pain: A pilot randomized controlled trial. *Clin Gerontol* 2016;**39**(5):428-48. https://doi.org/10.1080/07317115.2016.1171818

[27] The British Pain Society. *Participant Information for Pain Management Programmes* no. 978-0-9561386-5-1; 2013.

[28] Andersson G, Johansson C, Nordlander A, Asmundson GJG. Chronic Pain in Older Adults: A Controlled Pilot Trial of a Brief Cognitive-Behavioural Group Treatment. *Behav Cogn Psychother* 2012;**40**(2):239-44. https://doi.org/10.1017/S1352465811000646

[29] Baird CL, Sands L. A pilot study of the effectiveness of guided imagery with progressive muscle relaxation to reduce chronic pain and mobility difficulties of osteoarthritis. *Pain Manag Nurs* 2004;**5**(3):97-104. https://doi.org/10.1016/j.pmn.2004.01.003

[30] Carmody TP, Duncan CL, Huggins J, Solkowitz SN, Lee SK, Reyes N*, et al.* Telephone-Delivered Cognitive-Behavioral Therapy for Pain Management Among Older Military Veterans: A Randomized Trial. *Psychol Serv* 2013;**10**(3):265-75. https://doi.org/10.1037/a0030944

[31] Hasegawa T, Nishi K, Nakashima A, Moriuchi T, Iso N, Koseki H*, et al.* Effects of attentional bias modification on chronic low back pain in older outpatients: A randomized crossover trial (pilot study). *Medicine (Baltimore)* 2021;**100**(45):e27738. https://doi.org/10.1097/MD.0000000000027738

[32] Walsh N, Jones L, Phillips S, Thomas R, Odondi L, Palmer S*, et al.* Facilitating Activity and Self-management for people with Arthritic knee, hip or lower back pain (FASA): A cluster randomised controlled trial. *Musculoskelet Sci Pract* 2020;**50**:102271. https://doi.org/10.1016/j.msksp.2020.102271

[33] Yarns BC, Lumley MA, Cassidy JT, Steers WN, Osato S, Schubiner H*, et al.* Emotional awareness and expression therapy achieves greater pain reduction than cognitive behavioral therapy in older adults with chronic musculoskeletal pain: A preliminary randomized comparison trial. *Pain Med* 2020;**21**(11):2811-22. https://doi.org/10.1093/pm/pnaa145

[34] Bruckenthal P, Broderick J. Nurse Practitioners Can Effectively Deliver Coping Skills Training to Patients with Osteoarthritis. *Pain Manag Nurs* 2016;**17**(2):96. https://doi.org/10.1016/j.pmn.2016.02.016

[35] Cheng S-T, Chan KL, Lau RWL, Mok MHT, Chen PP, Chow YF*, et al.* A multicomponent intervention for the management of chronic pain in older adults: study protocol for a randomized controlled trial. *Trials* 2017;**18**(1):528. https://doi.org/10.1186/s13063-017-2270-3

[36] Ersek M, Turner JA, Cain KC, Kemp CA. Chronic pain self-management for older adults: a randomized controlled trial [ISRCTN11899548]. *BMC Geriatr* 2004;**4**(1):7-. https://doi.org/10.1186/1471-2318-4-7

[37] Kemp CA, Ersek M, Turner JA. A descriptive study of older adults with persistent pain: use and perceived effectiveness of pain management strategies [ISRCTN11899548]. *BMC Geriatr* 2005;**5**(1):12-. https://doi.org/10.1186/1471-2318-5-12

[38] Fanning J, Brooks AK, Hsieh KL, Kershner K, Furlipa J, Nicklas BJ*, et al.* The Effects of a Pain Management-Focused Mobile Health Behavior Intervention on Older Adults' Self-efficacy, Satisfaction with Functioning, and Quality of Life: a Randomized Pilot Trial. *Int J Behav Med* 2022;**29**(2):240-6. https://doi.org/10.1007/s12529-021-10003-3

[39] Fanning J, Brooks AK, Ip E, Nicklas BJ, Rejeski WJ. A Mobile Health Intervention to Reduce Pain and Improve Health (MORPH) in Older Adults With Obesity: Protocol for the MORPH Trial. *JMIR Res Protoc* 2018;**7**(5):e128. https://doi.org/10.2196/resprot.9712

[40] Groupp E, Haas M, Fairweather A, Ganger B, Attwood M. Recruiting Seniors With Chronic Low Back Pain for a Randomized Controlled Trial of a Self-Management Program. *J Manipulative Physiol Ther* 2005;**28**(2):97-102. https://doi.org/10.1016/j.jmpt.2005.01.004

[41] Hasegawa T. *Effects using attentional bias modification for the elderly people with chronic low back pain at outpatient*. 2019. URL: https://center6.umin.ac.jp/cgi-open-bin/ctr_e/ctr_view.cgi?recptno=R000033624 (Accessed 20 Jun 2023).

[42] Hurley M. *Effective and cost-effective rehabilitation for knee pain in a community population ISRCTN94658828*. 2010. URL: https://doi.org/10.1186/ISRCTN94658828 (Accessed 20 Jun 2023).

[43] Hurley MV, Walsh NE, Mitchell H, Nicholas J, Patel A. Long‐term outcomes and costs of an integrated rehabilitation program for chronic knee pain: A pragmatic, cluster randomized, controlled trial. *Arthritis care & research (2010)* 2012;**64**(2):238-47. https://doi.org/10.1002/acr.20642

[44] Tse M. *Dyadic Pain Management Program for Older Adults and Informal Caregivers With Chronic Pain*. 2020. URL: https://clinicaltrials.gov/ct2/show/NCT04106271 (Accessed 20 Jun 2023).

[45] Morone NE, Greco CM, Rollman BL, Moore CG, Lane B, Morrow L*, et al.* The design and methods of the aging successfully with pain study. *Contemporary clinical trials* 2012;**33**(2):417-25. https://doi.org/10.1016/j.cct.2011.11.012

[46] Nicholas MK, Asghari A, Blyth FM, Wood BM, Murray R, McCabe R*, et al.* Long-term outcomes from training in self-management of chronic pain in an elderly population: a randomized controlled trial. *Pain* 2017;**158**(1).

[47] Rini C. *Internet-Based Osteoarthritis Pain Coping Skills Intervention (PainCOACH) NCT01638871*. 2017. URL: https://clinicaltrials.gov/ct2/show/NCT01638871 (Accessed 20 Jun 2023).

[48] Von Korff M, Vitiello MV, McCurry SM, Balderson BH, Moore AL, Baker LD*, et al.* Group interventions for co-morbid insomnia and osteoarthritis pain in primary care: the lifestyles cluster randomized trial design. *Contemp Clin Trials* 2012;**33**(4):759-68. https://doi.org/10.1016/j.cct.2012.03.010

[49] Walsh N, Cramp F, Palmer S, Pollock J, Hampson L, Gooberman-Hill R*, et al.* Exercise and self-management for people with chronic knee, hip or lower back pain: a cluster randomised controlled trial of clinical and cost-effectiveness. Study protocol. *Physiotherapy* 2013;**99**(4):352-7. https://doi.org/10.1016/j.physio.2012.09.002

[50] Ackerman IN, Buchbinder R, Osborne RH. Challenges in Evaluating an Arthritis Self-management Program for People with Hip and Knee Osteoarthritis in Real-world Clinical Settings. *The Journal of Rheumatology* 2012;**39**(5):1047. https://doi.org/10.3899/jrheum.111358

[51] Alp A, Kanat E, Yurtkuran M. Efficacy of a self-management program for osteoporotic subjects. *American Journal of Physical Medicine & Rehabilitation* 2007;**86**(8):633-40. https://doi.org/10.1097/PHM.0b013e31806dd428

[52] Baker K, LaValley MP, Brown C, Felson DT, Ledingham A, Keysor JJ. Efficacy of Computer-based Telephone Counseling on Long-term Adherence to Strength Training in Elders with Knee Osteoarthritis: A Randomized Trial. *Arthritis Care Res (Hoboken)* 2019.

[53] Basler H-D, Bertalanffy H, Quint S, Wilke A, Wolf U. TTM-based counselling in physiotherapy does not contribute to an increase of adherence to activity recommendations in older adults with chronic low back pain – A randomised controlled trial. *Eur J Pain* 2007;**11**(1):31-7. https://doi.org/10.1016/j.ejpain.2005.12.009

[54] Bezalel T, Carmeli E, Katz-Leurer M. The effect of a group education programme on pain and function through knowledge acquisition and home-based exercise among patients with knee osteoarthritis: A parallel randomised single-blind clinical trial. *Physiotherapy* 2010;**96**(2):137-43. https://doi.org/10.1016/j.physio.2009.09.009

[55] Bilterys T, Kregel J, Nijs J, Meeus M, Danneels L, Cagnie B*, et al.* Influence of education level on the effectiveness of pain neuroscience education: A secondary analysis of a randomized controlled trial. *Musculoskelet Sci Pract* 2022;**57**:102494. https://doi.org/10.1016/j.msksp.2021.102494

[56] Birch S, Stilling M, Mechlenburg I, Hansen TB. No effect of cognitive behavioral patient education for patients with pain catastrophizing before total knee arthroplasty: a randomized controlled trial. *Acta Orthopaedica* 2020;**91**(1):98-103. https://doi.org/10.1080/17453674.2019.1694312

[57] Bobek DB, Aksentijevic JA, Mijacika ML. The significance of the oa school at the time of the covid-19 pandemia. *Osteoporosis International* 2022;**32**(suppl.1):S404-S5. https://doi.org/10.1007/s00198-021-06125-9

[58] Brown CA, Jones AKP. Psychobiological Correlates of Improved Mental Health in Patients With Musculoskeletal Pain After a Mindfulness-based Pain Management Program. *Clin J Pain* 2013;**29**(3).

[59] Burns JW, Van Dyke BP, Newman AK, Morais CA, Thorn BE. Cognitive behavioral therapy (CBT) and pain education for people with chronic pain: Tests of treatment mechanisms. *J Consult Clin Psychol* 2020;**88**(11):1008‐18. https://doi.org/10.1037/ccp0000612

[60] Buszewicz M, Rait G, Griffin M, Nazareth I, Patel A, Atkinson A*, et al.* Self management of arthritis in primary care: randomised controlled trial. *BMJ* 2006;**333**(7574):879. https://doi.org/10.1136/bmj.38965.375718.80

[61] Cederbom S, Denison E, Bergland A. A behavioral medicine intervention for community-dwelling older adults with chronic musculoskeletal pain: Protocol for a randomized controlled trial. *J Pain Res* 2017;**10**:845-53. https://doi.org/10.2147/JPR.S129648

[62] Cederbom S, Leveille SG, Bergland A. Effects of a behavioral medicine intervention on pain, health, and behavior among community-dwelling older adults: a randomized controlled trial. *Clin Interv Aging* 2019;**14**:1207-20. https://doi.org/10.2147/CIA.S208102

[63] Coleman S, Briffa NK, Carroll G, Inderjeeth C, Cook N, McQuade J. A randomised controlled trial of a self-management education program for osteoarthritis of the knee delivered by health care professionals. *Arthritis Res Ther* 2012;**14**(1):R21. https://doi.org/10.1186/ar3703

[64] Corrêa Dias R, Domingues Dias JM, Ramos LR. Impact of an exercise and walking protocol on quality of life for elderly people with OA of the knee. *Physiother Res Int* 2003;**8**(3):121-30.

[65] Coupé VMH, Veenhof C, van Tulder MW, Dekker J, Bijlsma JWJ, Van den Ende CHM. The cost effectiveness of behavioural graded activity in patients with osteoarthritis of hip and/or knee. *Ann Rheum Dis* 2007;**66**(2):215-21. https://doi.org/10.1136/ard.2006.054478

[66] Foo CN, Arumugam M, Lekhraj R, Lye MS, Mohd-Sidik S, Jamil Osman Z. Effectiveness of Health-Led Cognitive Behavioral-Based Group Therapy on Pain, Functional Disability and Psychological Outcomes among Knee Osteoarthritis Patients in Malaysia. *Int J Environ Res Public Health* 2020;**17**(17):26. https://doi.org/10.3390/ijerph17176179

[67] Fries JF, Carey C, McShane DJ. Patient education in arthritis: randomized controlled trial of a mail-delivered program. *J Rheumatol* 1997;**24**(7):1378-83.

[68] Hausmann LRM, Youk A, Kwoh CK, Gallagher RM, Weiner DK, Vina ER*, et al.* Effect of a Positive Psychological Intervention on Pain and Functional Difficulty Among Adults With Osteoarthritis: A Randomized Clinical Trial. *JAMA Network Open* 2018;**1**(5):e182533-e. https://doi.org/10.1001/jamanetworkopen.2018.2533

[69] Hay EM, Foster NE, Thomas E, Peat G, Phelan M, Yates HE*, et al.* Effectiveness of community physiotherapy and enhanced pharmacy review for knee pain in people aged over 55 presenting to primary care: pragmatic randomised trial. *BMJ* 2006;**333**(7576):995. https://doi.org/10.1136/bmj.38977.590752.0B

[70] Holm PM, Petersen KK, Wernbom M, Schroder HM, Arendt-Nielsen L, Skou ST. Strength training in addition to neuromuscular exercise and education in individuals with knee osteoarthritis-the effects on pain and sensitization. *Eur J Pain* 2021;**25**(9):1898-911. https://doi.org/10.1002/ejp.1796

[71] Hruschak V, Rosen D, Tierney M, Eack SM, Wasan AD, Cochran G. Integrated Psychosocial Group Treatment: A Randomized Pilot Trial of a Harm Reduction and Preventive Approach for Patients with Chronic Pain at Risk of Opioid Misuse. *Pain Med* 2021;**22(9)**:2007-18. https://doi.org/10.1093/pm/pnaa461

[72] Hughes SL, Seymour RB, Campbell RT, Huber G, Pollak N, Sharma L*, et al.* Long-Term Impact of Fit and Strong! on Older Adults With Osteoarthritis. *Gerontologist* 2006;**46**(6):801-14. https://doi.org/10.1093/geront/46.6.801

[73] Hunt MA, Keefe FJ, Bryant C, Metcalf BR, Ahamed Y, Nicholas MK*, et al.* A physiotherapist-delivered, combined exercise and pain coping skills training intervention for individuals with knee osteoarthritis: A pilot study. *Knee* 2012;**20**(2):106-12. https://doi.org/10.1016/j.knee.2012.07.008

[74] Irvine AB, Russell H, Manocchia M, Mino DE, Cox Glassen T, Morgan R*, et al.* Mobile-Web App to Self-Manage Low Back Pain: Randomized Controlled Trial. *J Med Internet Res* 2015;**17**(1):e1. https://doi.org/10.2196/jmir.3130

[75] Jensen MP, Mendoza ME, Ehde DM, Patterson DR, Molton IR, Dillworth TM*, et al.* Effects of hypnosis, cognitive therapy, hypnotic cognitive therapy, and pain education in adults with chronic pain: a randomized clinical trial. *Pain* 2020;**161**(10):2284‐98. https://doi.org/10.1097/j.pain.0000000000001943

[76] Jinnouchi H, Matsudaira K, Kitamura A, Kakihana H, Oka H, Hayama-Terada M*, et al.* Effects of brief self-exercise education on the management of chronic low back pain: A community-based, randomized, parallel-group pragmatic trial. *Mod Rheumatol* 2021;**31**(4):890-8. https://doi.org/10.1080/14397595.2020.1823603

[77] Lin EHB, Katon W, Von Korff M, Tang L, Williams JJW, Kroenke K*, et al.* Effect of Improving Depression Care on Pain and Functional Outcomes Among Older Adults With Arthritis: A Randomized Controlled Trial. *JAMA* 2003;**290**(18):2428-9. https://doi.org/10.1001/jama.290.18.2428

[78] Macfarlane GJ, Beasley M, Scott N, McNamee P, McBeth J, Prescott G*, et al.* Maintaining musculoskeletal health: A randomised controlled trial of cognitive behaviour therapy among people at high risk of developing chronic widespread pain. *Rheumatology (Oxford)* 2020;**59**(suppl.2):ii7. https://doi.org/10.1093/rheumatology/keaa110.014

[79] McCurry SM, Zhu W, Von Korff M, Wellman R, Morin CM, Thakral M*, et al.* Effect of Telephone Cognitive Behavioral Therapy for Insomnia in Older Adults With Osteoarthritis Pain: a Randomized Clinical Trial. *JAMA Intern Med* 2021;**181**(4):530‐8. https://doi.org/10.1001/jamainternmed.2020.9049

[80] Messier SP, Loeser RF, Miller GD, Morgan TM, Rejeski WJ, Sevick MA*, et al.* Exercise and dietary weight loss in overweight and obese older adults with knee osteoarthritis: The arthritis, diet, and activity promotion trial. *Arthritis Rheum* 2004;**50**(5):1501-10. https://doi.org/10.1002/art.20256

[81] Miyamoto GC, Fagundes FRC, de Melo do Espirito Santo C, de Luna Teixeira FM, Tonini TV, Prado FT*, et al.* Education With Therapeutic Alliance Did Not Improve Symptoms in Patients With Chronic Low Back Pain and Low Risk of Poor Prognosis Compared to Education Without Therapeutic Alliance: A Randomized Controlled Trial. *J Orthop Sports Phys Ther* 2021;**51**(8):392-400. https://doi.org/10.2519/jospt.2021.9636

[82] Murphy SL, Strasburg DM, Lyden AK, Smith DM, Koliba JF, Dadabhoy DP*, et al.* Effects of activity strategy training on pain and physical activity in older adults with knee or hip osteoarthritis: A pilot study. *Arthritis Rheum* 2008;**59**(10):1480-7. https://doi.org/10.1002/art.24105

[83] Murphy SL, Lyden AK, Smith DM, Dong Q, Koliba JF. Effects of a tailored activity pacing intervention on pain and fatigue for adults with osteoarthritis. *Am J Occup Ther* 2010;**64**(6):869-76. https://doi.org/10.5014/ajot.2010.09198

[84] Nunes ACL. Pain education and exercise program supported by cell phone for older adults with low back pain. *https://trialsearchwhoint/Trial2aspx?TrialID=RBR-653xcn* 2020.

[85] Østerås N, Hagen KB, Grotle M, Sand-Svartrud A-L, Mowinckel P, Aas E*, et al.* Exercise programme with telephone follow-up for people with hand osteoarthritis - Protocol for a randomised controlled trial. *BMC Musculoskelet Disord* 2014;**15**(1):82-. https://doi.org/10.1186/1471-2474-15-82

[86] Petrozzi MJ, Spencer G, Mackey MG. A process evaluation of the Mind Your Back trial examining psychologically informed physical treatments for chronic low back pain. *Chiropr Man Therap* 2021;**29**(1):32. https://doi.org/10.1186/s12998-021-00389-y

[87] Pimm TJ, Williams LJ, Reay M, Pickering S, Lota R, Coote L*, et al.* An evaluation of a digital pain management programme: clinical effectiveness and cost savings. *British Journal of Pain* 2020;**14**(4):238‐49. https://doi.org/10.1177/2049463719865286

[88] Pratscher S, Mickle AM, Marks JG, Rocha H, Bartsch F, Schmidt J*, et al.* Optimizing Chronic Pain Treatment with Enhanced Neuroplastic Responsiveness: A Pilot Randomized Controlled Trial. *Nutrients* 2021;**13**(5):05. https://doi.org/10.3390/nu13051556

[89] Schulz C, Evans R, Maiers M, Schulz K, Leininger B, Bronfort G. Spinal manipulative therapy and exercise for older adults with chronic low back pain: A randomized clinical trial. *Chiropr Man Therap* 2019;**27**(1):21-. https://doi.org/10.1186/s12998-019-0243-1

[90] Skovbo MH, Agerbo K, Jakobsen A, Clausen SA, Langagergaard V, Rolving N. Description of content, structure and theoretical model of a group-based pain management programme in the treatment of patients with persistent non-specific low back pain and psychological risk factors in a secondary sector setting. *Clin Rehabil* 2021;**35**(8):1077‐88. https://doi.org/10.1177/0269215521995185

[91] Suso-Ribera C, Castilla D, Zaragoza I, Mesas A, Server A, Medel J*, et al.* Telemonitoring in chronic pain management using smartphone apps: A randomized controlled trial comparing usual assessment against app-based monitoring with and without clinical alarms. *Int J Environ Res Public Health* 2020;**17**(18):1-23. https://doi.org/10.3390/ijerph17186568

[92] Swerissen H, Belfrage J, Weeks A, Jordan L, Walker C, Furler J*, et al.* A randomised control trial of a self-management program for people with a chronic illness from Vietnamese, Chinese, Italian and Greek backgrounds. *Patient Educ Couns* 2006;**64**(1):360-8. https://doi.org/10.1016/j.pec.2006.04.003

[93] Tak E, Staats P, Van Hespen A, Hopman-Rock M. The effects of an exercise program for older adults with osteoarthritis of the hip. *The Journal of Rheumatology* 2005;**32**(6):1106.

[94] Tang NKY, Moore C, Parsons H, Sandhu HK, Patel S, Ellard DR*, et al.* Implementing a hybrid cognitive-behavioural therapy for pain-related insomnia in primary care: lessons learnt from a mixed-methods feasibility study. *BMJ Open* 2020;**10**(3):e034764. https://doi.org/10.1136/bmjopen-2019-034764

[95] Tse MMY, Ng SSM, Lee PH, Bai X, Lo R, Tang SK*, et al.* Effectiveness of a Peer-Led Pain Management Program in Relieving Chronic Pain and Enhancing Pain Self-Efficacy Among Older Adults: A Clustered Randomized Controlled Trial. *Front Med* 2021;**8**:709141. https://doi.org/10.3389/fmed.2021.709141

[96] Turner BJ, Rodriguez N, Bobadilla R, Hernandez AE, Yin Z. Chronic Pain Self-Management Program for Low-Income Patients: Themes from a Qualitative Inquiry. *Pain Med* 2020;**21**(2):e1-e8. https://doi.org/10.1093/pm/pny192

[97] Vandermost M, Bagraith KS, Kennedy H, Doherty D, Kilner S, Sterling M*, et al.* Improvement in pain interference and function by an allied health pain management program: Results of a randomized trial. *Eur J Pain* 2021;**25(10)**:2226-41. https://doi.org/10.1002/ejp.1836

[98] Vitiello MV, Rybarczyk B, Von Korff M, Stepanski EJ. Cognitive behavioral therapy for insomnia improves sleep and decreases pain in older adults with co-morbid insomnia and osteoarthritis. *J Clin Sleep Med* 2009;**5**(4):355-62. https://doi.org/10.5664/jcsm.27547

[99] Vitiello MV, Zhu W, Von Korff M, Wellman R, Morin CM, Yeung K*, et al.* Long-term improvements in sleep, pain, depression, and fatigue in older adults with comorbid osteoarthritis pain and insomnia. *Sleep* 2022;**45**(2):14. https://doi.org/10.1093/sleep/zsab231

[100] Yip YB, Sit JW, Fung KK, Wong DY, Chong SY, Chung LH*, et al.* Impact of an Arthritis Self-Management Programme with an added exercise component for osteoarthritic knee sufferers on improving pain, functional outcomes, and use of health care services: An experimental study. *Patient Educ Couns* 2007;**65**(1):113-21. https://doi.org/10.1016/j.pec.2006.06.019

[101] Yip Y-B, Sit JW, Wong DYS, Chong SYC, Chung L-H. A 1-year follow-up of an experimental study of a self-management arthritis programme with an added exercise component of clients with osteoarthritis of the knee. *Psychol Health Med* 2008;**13**(4):402-14. https://doi.org/10.1080/13548500701584030

[ 102] Thorn BE. *Cognitive Therapy for Chronic Pain: A Step-By-Step Guide*. New York, NY: Guilford Publications; 2017.

[103] Lorig KR, Holman HR. Self-management education: history, definition, outcomes, and mechanisms. *Ann Behav Med* 2003;**26**(1):1-7.

[104] Bandura A. *Self-Efficacy: The Exercise of Control*. New York, NY: W H Freeman; 1997.

[105] Ritterband LM, Thorndike FP, Cox DJ, Kovatchev BP, Gonder-Frederick LA. A behavior change model for internet interventions. *Ann Behav Med* 2009;**38**(1):18-27. https://doi.org/10.1007/s12160-009-9133-4

[106] Gatchel RJ, Peng YB, Peters ML, Fuchs PN, Turk DC. The biopsychosocial approach to chronic pain: scientific advances and future directions. *Psychol Bull* 2007;**133**(4):581-624. https://doi.org/10.1037/0033-2909.133.4.581

[107] Fredrickson BL. The role of positive emotions in positive psychology: The broaden-and-build theory of positive emotions. *Am Psychol* 2001;**56**(3):218. https://doi.org/10.1037/0003-066X.56.3.218

[108] DiLauro M, Pereira A, Carr J, Chiu M, Wesson V. Spousal caregivers and persons with dementia: Increasing participation in shared leisure activities among hospital-based dementia support program participants. *Dementia* 2017;**16**(1):9-28. https://doi.org/10.1177/1471301215570680

[109] Kabat-Zinn J. Mindfulness-Based Interventions in Context: Past, Present, and Future. *Clin Psychol (New York)* 2003;**10**(2):144-56. https://doi.org/10.1093/clipsy.bpg016

[110] Knowles MS. *The Adult Learner: A Neglected Species*. 3rd edn. Houston, TX: Gulf; 1984.

[111] Dworkin SF, Von Korff MR, LeResche L. Epidemiologic studies of chronic pain: A dynamic-ecologic perspective. *Ann Behav Med* 1992;**14**(1):3-11. https://doi.org/10.1093/abm/14.1.3
